# Supplementary material for: A single Na+-Pi cotransporter in Toxoplasma plays key roles in phosphate import and control of parasite osmoregulation
Source: PLoS Pathog. 2020 Dec 31;16(12):e1009067. doi: 10.1371/journal.ppat.1009067 (PMC7817038; doi:10.1371/journal.ppat.1009067)
Supplement: S1 Fig — (PDF) [file ppat.1009067.s001.pdf]

```
ScPHO89    -------------MALHQFDYIFAIAMLFAFLDAFNIGANDVANSFASSISSRSLKYWQA    47
TgPiT      MEAVAELSAPSLAGAPGEYTWIVAVAGVTCFLTAFAIGANDVANTFSSSVGSRAIPLWAA    60
PfPiT      ------------MVTGPDMLWLVITSGIACFFMAFVTGANDIANTFSTSIGSKAISIKKA    48
                         :  :  ::.  : : .*: **  ****:**:*::*:.*:::    *

ScPHO89    MVLAGLCEFLGAVLAGARVSGTIKNNIIDSSIFTNDPAVLMLTMTSALIGSSCWLTFATA    107
TgPiT      IGMSAVLETVGATLLGGAVTDSIRSKIIDFEVFRETPSILMTGMLCALVGAGLWLFLANH    120
PfPiT      LIVAFFFEALGASLLGGTVTDSIRSKIINFQVFYDTPEFLMLGMCCALMGATVWLAVATR    108
           : :: . * :** * *. *:.:*:.:**: .:* : * .**  * .**:*:  ** .*.

ScPHO89    IGMPVSTTHSIVGGTIGAGIAAGGANGVVWGWSGVSQIIASWFIAPILAGAIAAIVFSIS    167
TgPiT      LGLPVSTTHSIIGALLGFGLASGNVRAVK--WTQVAFIVGSWVAAPLAASAAGATIFVCM    178
PfPiT      AGLPVSTTHSIIGALLGFGLATGNMKSIK--WEKINNIVISWLAAPILAGTCSAIAFTVL    166
            *:********:*. :* *:*:*. ..:   *  :  *: **. **: *.: .*  *

ScPHO89    RFSVLEVKSLERSIKNALLLVGVLVFATFSILTM---LIVWK--------GSPNLHL---    213
TgPiT      RRLILRSRQPLRRAKRFLWIFIYLITLTFSVFLVFKNFFELNVSCDQMVAGGRVEHFEPC    238
PfPiT      RMLILRKKNSFEIIKKMYWFLIFLITLPFSVFLIFHNPIVINTQCKM-KKDGKVIVSSPC    225
           *  :*. :.  .  *.   :.  *:   **:: :    :  :        ..

ScPHO89    --DDLSE----TET----AVSIVLTGAASIVYFIFFYPFYRRKVLDQDWT----LKLIDI    259
TgPiT      RISRWADAHSGTALGIAVALSVALTFVISC--LVYRFAFYRVESYRRRQKRSSRTEPRDA    296
```

```
ScPHO89    IHAQVNDRDMLSGDLKGM-YERSKFYDNRVEYIYSVLQAITAATMSFAHGANDVANATGP    428
TgPiT      -----HAEGSTEDDLVARLQTGAEVFDTETELFFSACQVVSACMGCIAHSANDTANAIGP    701
PfPiT      -----NGKGG---DPKNMENVIIENFDPQTEIVFSSLQIISAILGVVAQSANDTANAIGP    485
                : ..    *         : :* ..* .:*  * ::*    .*:.***.*** **

ScPHO89    LSAVYVIWKTNTIGAKSEVPVWVLAYGGVALVIGCWTYGYNIIKNLGNKMILQSPSRGFS    488
TgPiT      FAAILTVYQSGSADSEIGSPWYILLFGGLSMSLGLALLGYRVIKTVGVKLVKITPARGFS    761
PfPiT      FAAVFNTYNNGIR-GKIKVQWYILLFGGLSMSLGLSIMGYRVIKTVGMKLIKITPARGFT    544
           ::*:   ::..   .:     ::* :**::: :*    **.:**.:* *::  :*:***:

ScPHO89    IELAVAITTVMATQLGIPTSTTQIAVGGIVAVGLCNKD----------------------    526
TgPiT      MELGAAWTVLIFSAIGIPLSTTHCAVGSTVGVGLMEPKHPRRETGDGP------VAEGEE    815
PfPiT      IELISGLVVLFFSICGIPLSSTHCAVSSVIGLGLVEAKMNADNKRHARKSMDKDIIQVDK    604
           :**  . ..:: :  *** *:*: **.. :.:** : .

ScPHO89    ------------------LKSVNWRMVAWCYSGWFLTLPIAGLIAGIINGIILNAPRFGV    568
TgPiT      ------PKKRAVQCPVINTASVNWKLFGGVFVSWIITIAFSALVTAALFSFAAYSP----    865
PfPiT      DKSFTLTEKIKYPFSFLNTSCVNLRLFRTVFLSWILTVVFSATVTAGIYSFAAYSPSYIM    664
                               .** ::.   : .*::*: ::. ::. : .:   :*

ScPHO89    EYQMT     573
TgPiT      VS---     865
PfPiT      KMQTV     669
```

S1 Fig. Alignments of N-terminal and C-terminal sequences of TgPiT with ScPHO89 and PfPiT, showing the conserved glutamic residues (in green) in TgPiT for P_i import.
