# Supplementary figures and images for: A single Na+-Pi cotransporter in Toxoplasma plays key roles in phosphate import and control of parasite osmoregulation
Source: PLoS Pathog. 2020 Dec 31;16(12):e1009067. doi: 10.1371/journal.ppat.1009067 (PMC7817038; doi:10.1371/journal.ppat.1009067)

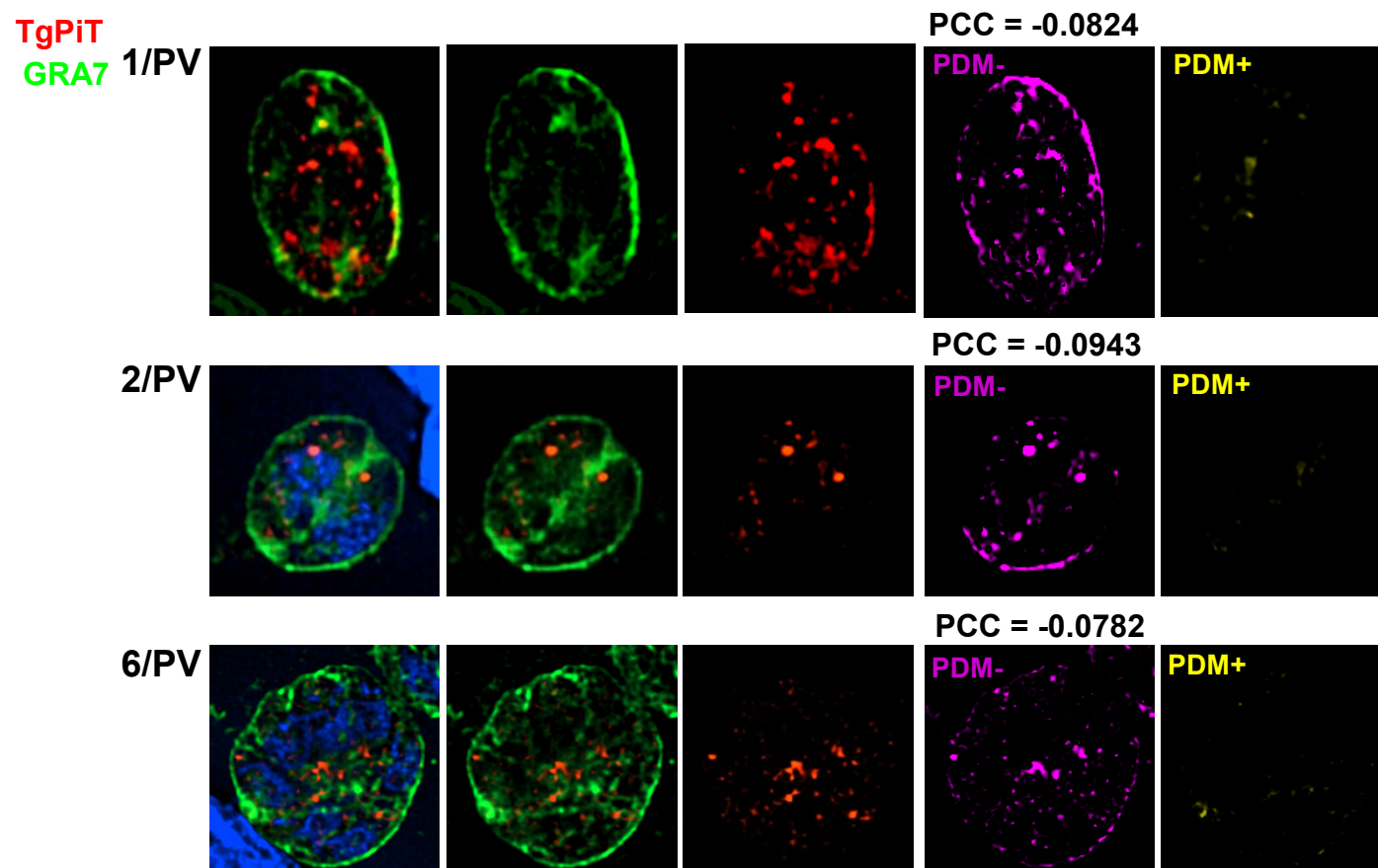

**S2 Fig. TgPiT localization in *Toxoplasma***

Supplement: S2 Fig — Fibroblasts were infected with T. gondii for 24 h before immunostaining using antibodies against TgPiT and GRA7 (dense granules and PV membrane). The Pearson’s Correlation Coefficient (PCC) and positive and negative product of the difference from the mean (PDM) channels were calculated. The negative PDM values (purple voxels) indicate no association of TgPiT with dense granules or the PV membrane. (PDF) [file ppat.1009067.s002.pdf]

**A**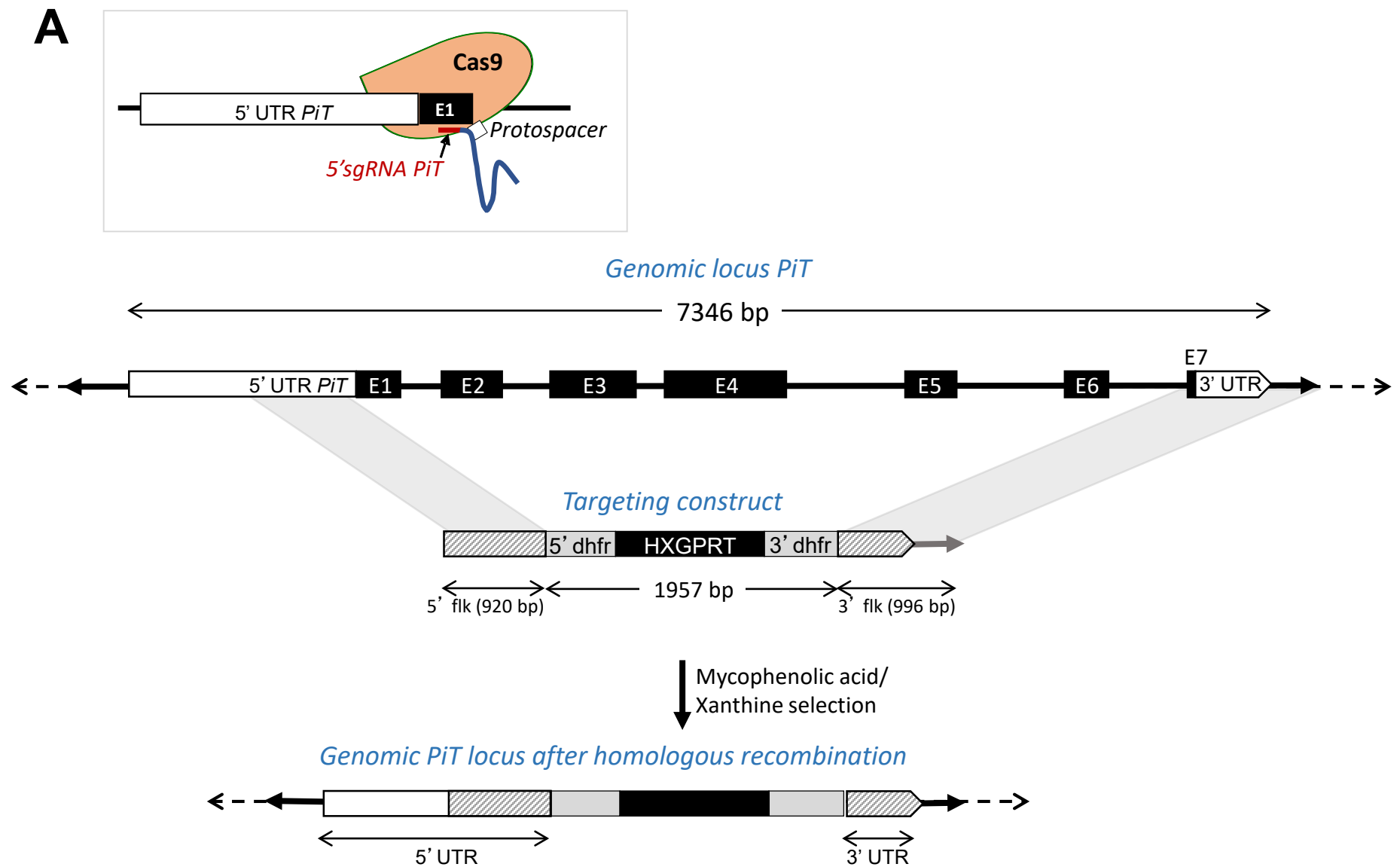**B**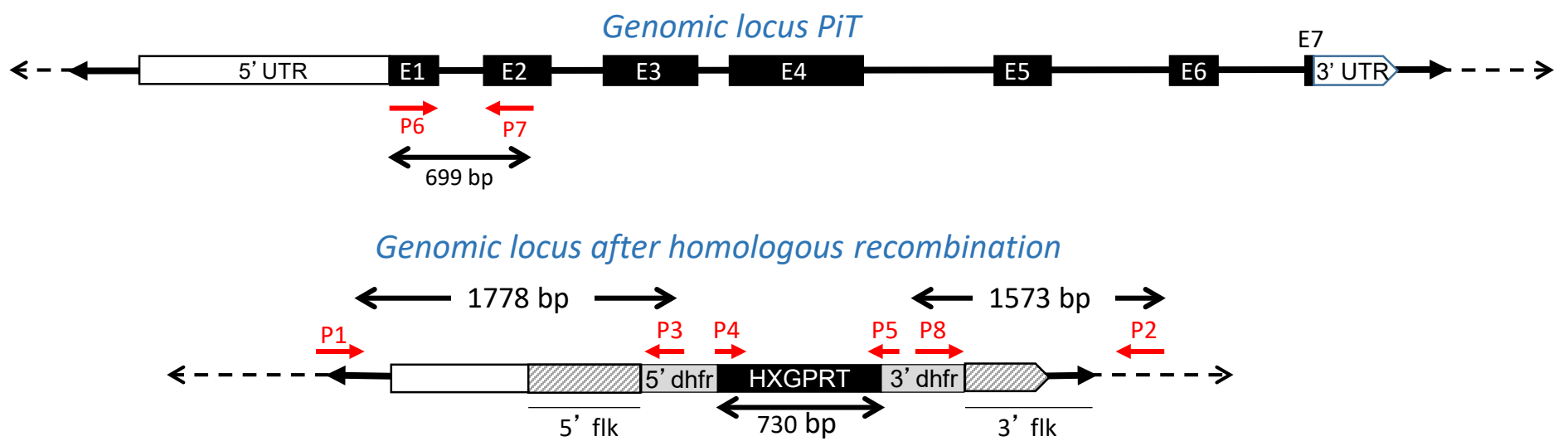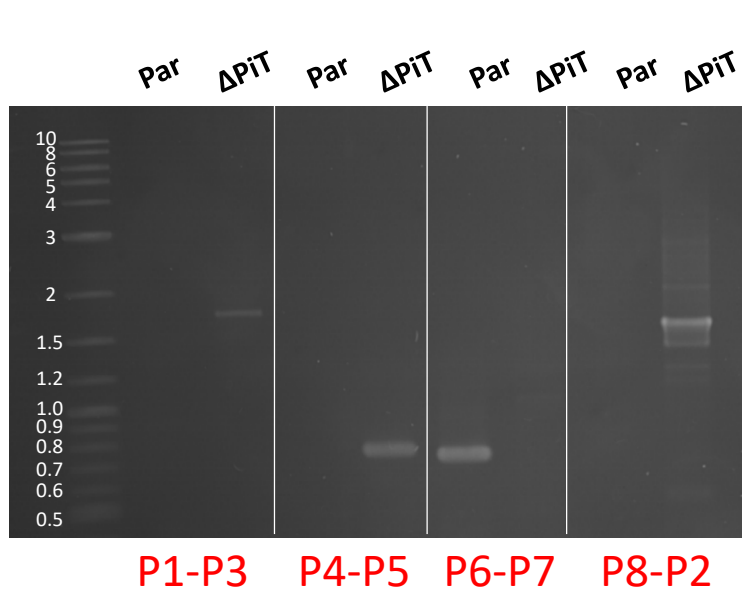**C**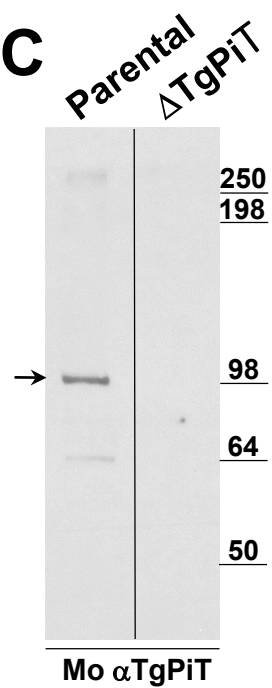**D**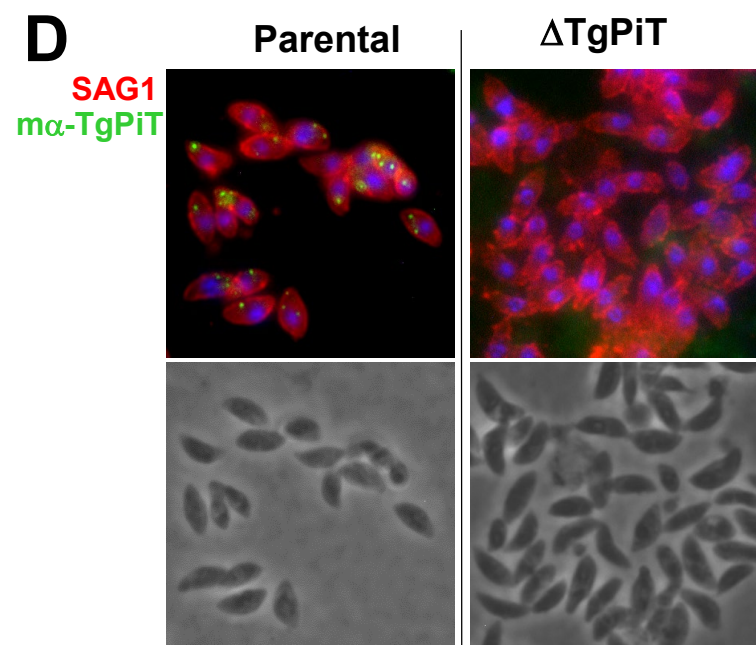**S3 Fig. Strategy for *PiT* deletion and verification**

Supplement: S3 Fig — A. Schematic depiction for PiT ablation from the Toxoplasma genome. B. Confirmation of the double homologous recombination by screening PCR using the primers shown in red (in B), by staining with anti-TgPiT antibodies on immunoblots of parasite lysates (in C; arrow showing a band at ~96 kD) and fixed extracellular parasites (in D), comparing the parental and knockout strains. (PDF) [file ppat.1009067.s003.pdf]

**A**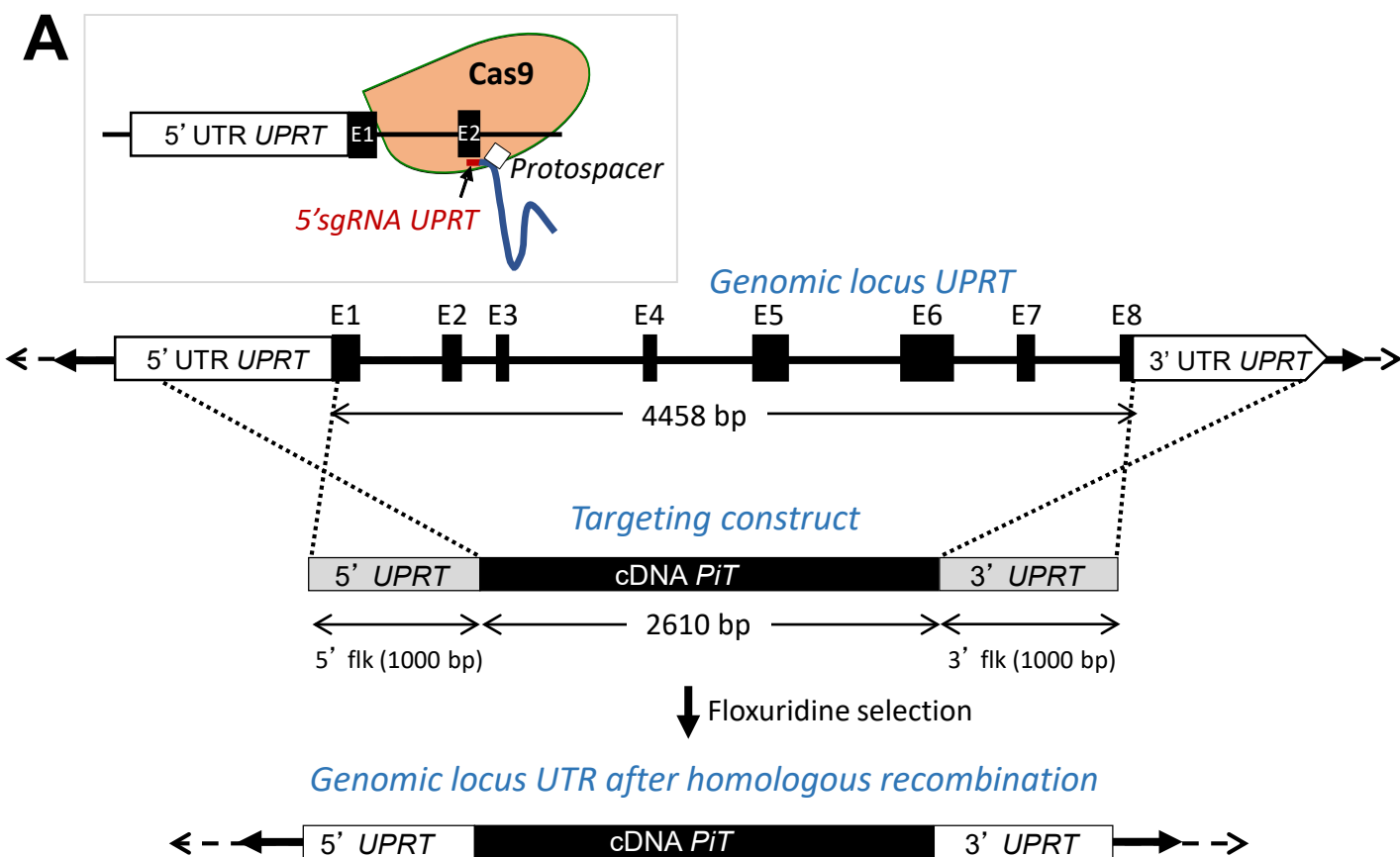**B**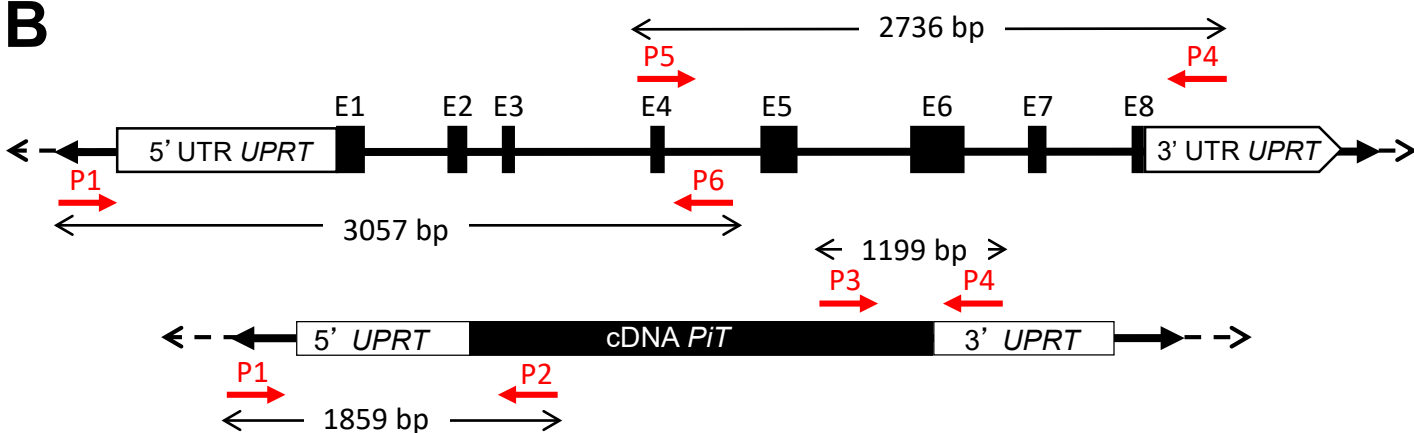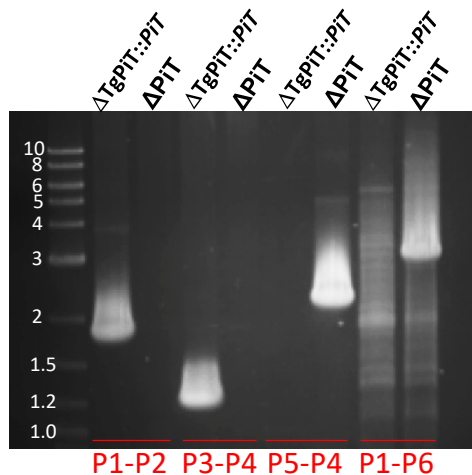**C**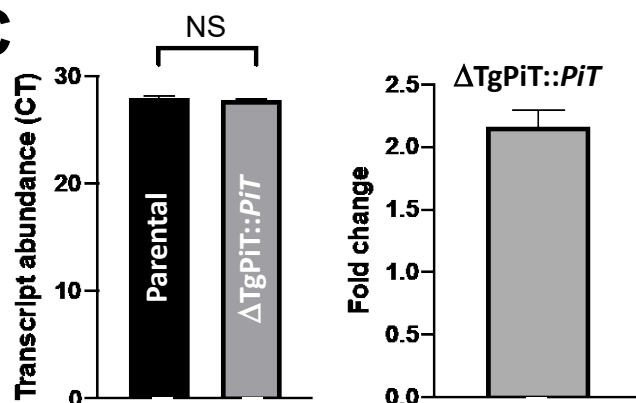

**S4 Fig. Strategy for complementation of  $\Delta$ TgPiT parasites and verification**

Supplement: S4 Fig — A. Schematic depiction for PiT reinsertion in TgPiT parasites. B. Confirmation of the double homologous recombination by screening PCR using the primers shown in red. C. Transcriptional profiles of TgPiT in the parental and complemented (TgPiT::PiT) strains using total RNA subjected to first-strand cDNA synthesis using oligo(dT) primers. Left: Absolute abundance of PiT transcripts in the parental and TgPiT::PiT strains. Right: Transcript abundance Log2 fold-change calculated by normalization with the housekeeping gene transcript, Tgα-actin. Data are means ± SD (n = 3 independent assays). NS (p = 0.2; Mann-Withney U test). (PDF) [file ppat.1009067.s004.pdf]

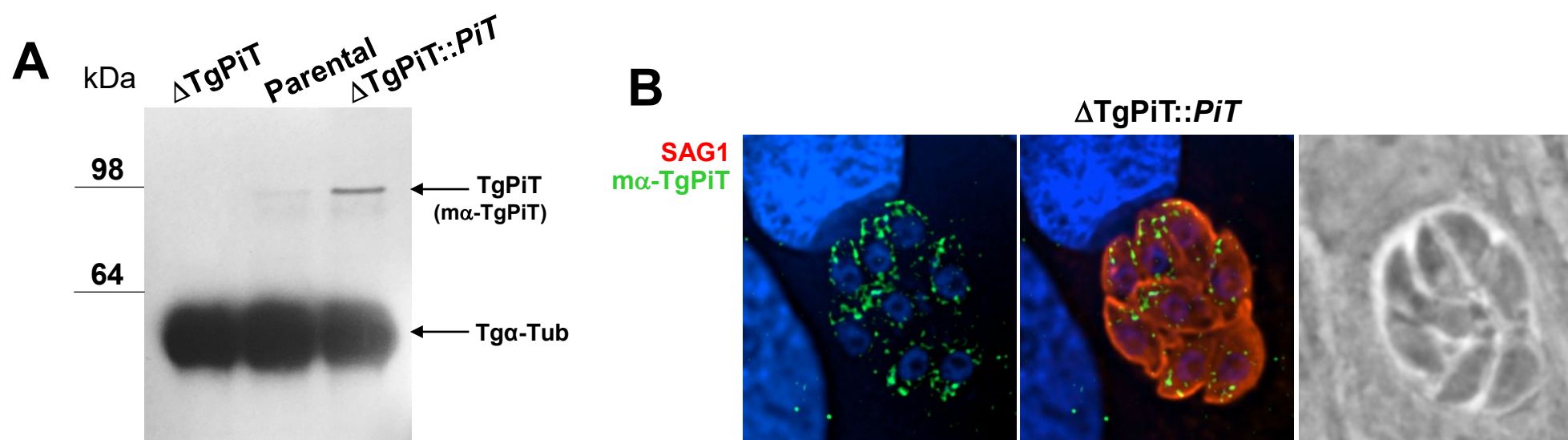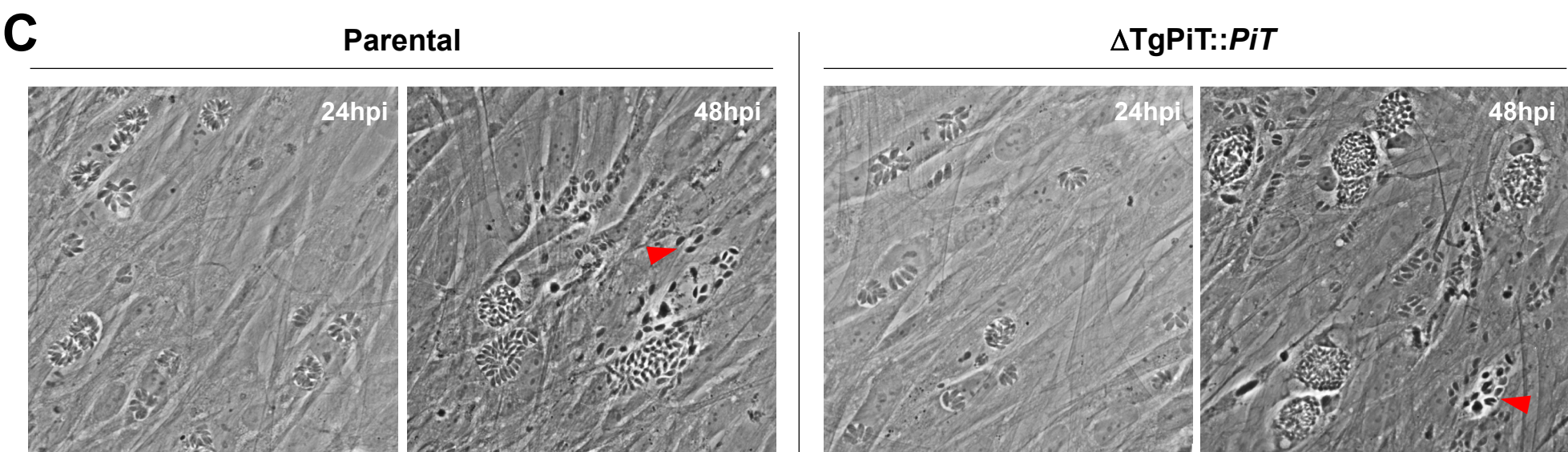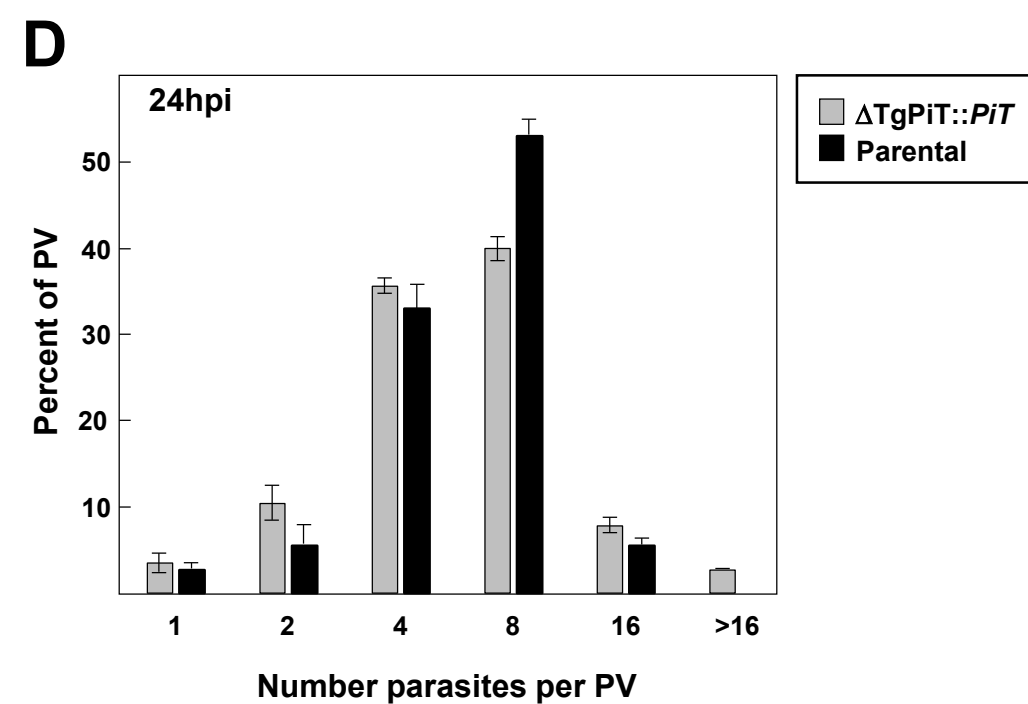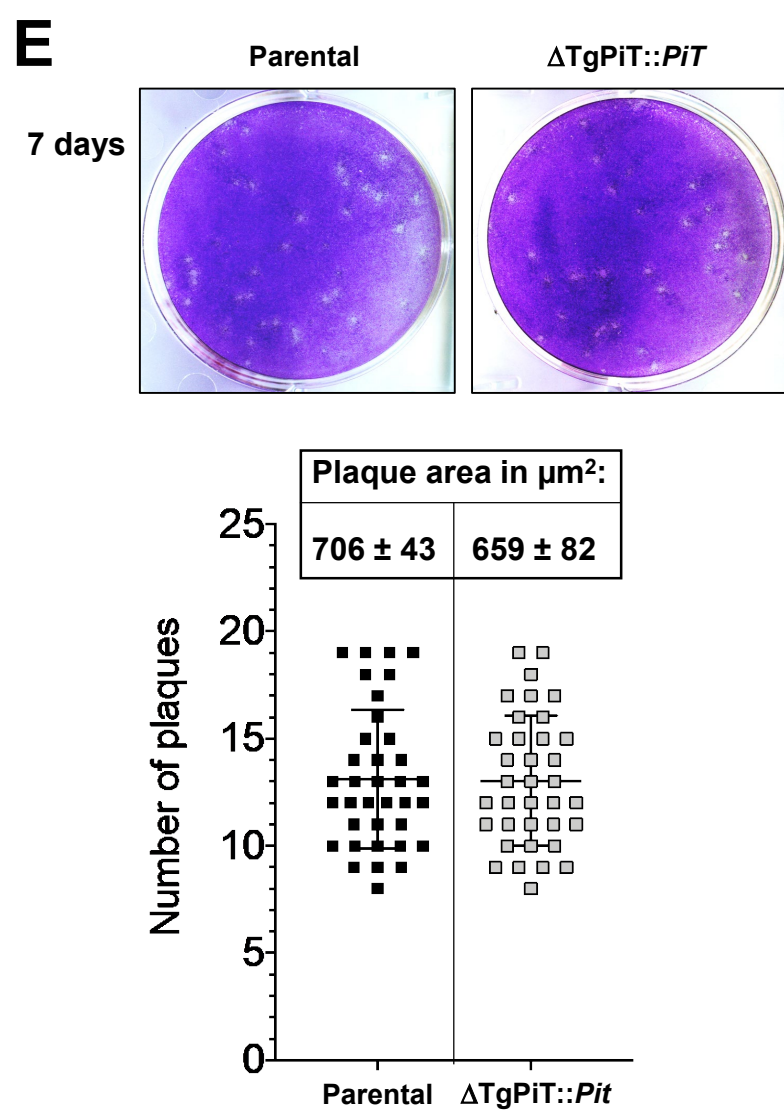

S5 Fig. Phenotype of TgPiT::*PiT* parasites

Supplement: S5 Fig — A. Western blot of parasite lysates from the parental, ΔTgPiT and ΔTgPiT::PiT strains probed with anti-TgPiT antibody showing TgPiT expressed in complemented parasites. Loading control: Toxoplasma α-tubulin (Tgα-Tub; 51 kDa) probed with mouse anti-α-tubulin antibody. B. IFA showing expression of TgPiT in ΔTgPiT::PiT parasites using anti-TgPiT antibodies. C-D. Microscopic observation of PV size in parental and ΔTgPiT::PiT parasites at 24 h and 48 h p.i. and parasite enumeration per PV showing no difference between the 2 strains. Arrowheads point to egressing parasites. E. Plaque assays for 7 days of parental and ΔTgPiT::PiT parasites infecting fibroblasts and dotplots to quantify lysis area, showing growth restoration to normal levels for the complemented strain. Differences in replication (D) and growth (E) between the two strains were not statistically different (unpaired Student’s t-test). (PDF) [file ppat.1009067.s005.pdf]
