# Supplementary material for: A single Na+-Pi cotransporter in Toxoplasma plays key roles in phosphate import and control of parasite osmoregulation
Source: PLoS Pathog. 2020 Dec 31;16(12):e1009067. doi: 10.1371/journal.ppat.1009067 (PMC7817038; doi:10.1371/journal.ppat.1009067)
Supplement: S6 Fig — Panels a-c: Volume distribution of parasites for each biological replicate consisting of three technical replicates. Data are mean ± SEM. Extracellular parasites were counted after gating on the basis of 6 different volumes, from 10 μm3 to 40 μm3, in 5 μm3 increments. The raw number of parasites counted at each gate was normalized to the raw number of parasites at the 10 μm3 gate, representing 100% of the parasite population, for each strain. After normalization, a linear regression analysis was performed on each curve, and the resulting equations was averaged and used to determine the mean volume for each parasite line. (PDF) [file ppat.1009067.s006.pdf]

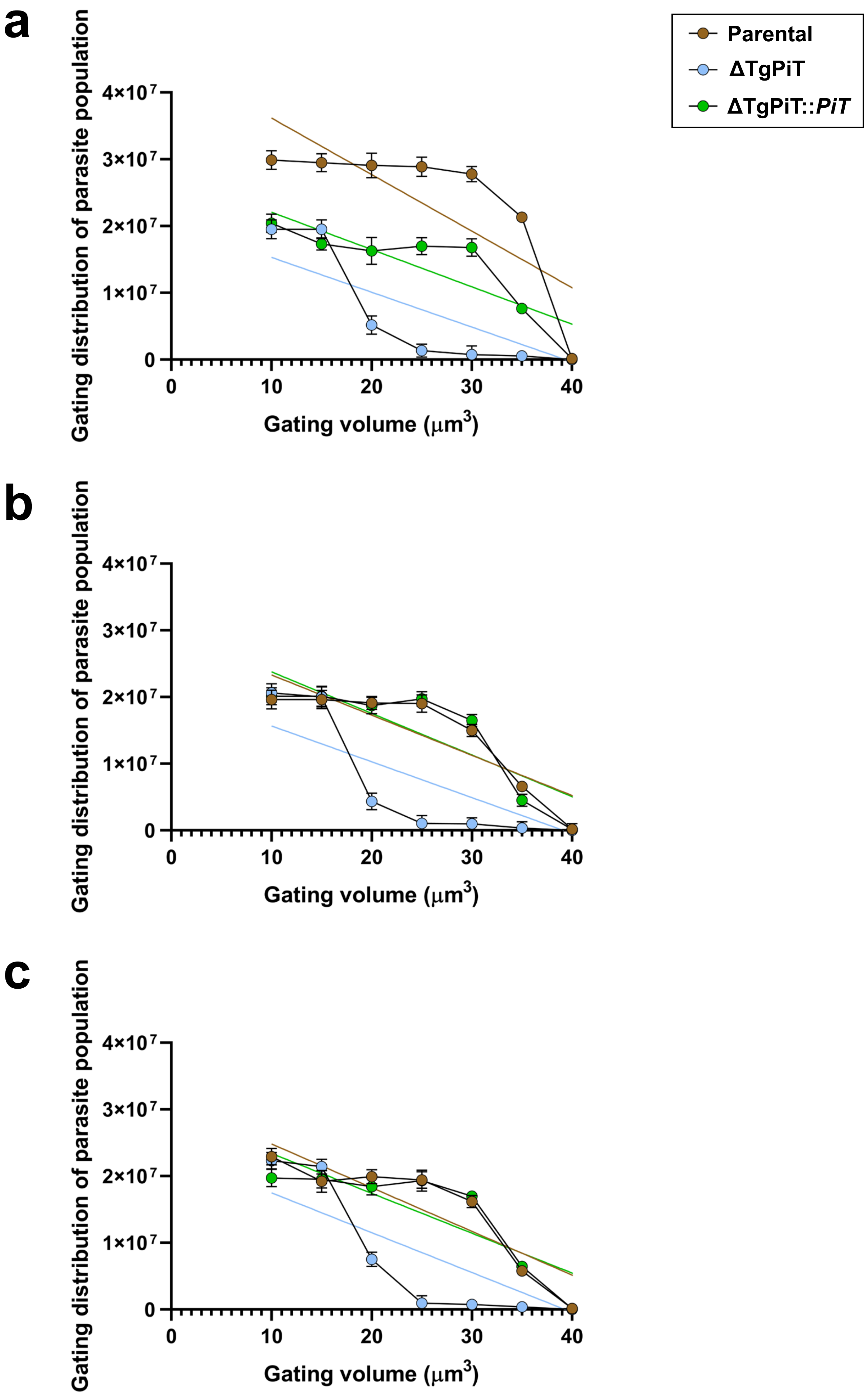

S6 Fig. Generation of the standard curves used to determine the cell volume for parental, ΔTgPiT and ΔTgPiT::PiT parasites using a Coulter counter
