# Supplementary material for: A single Na+-Pi cotransporter in Toxoplasma plays key roles in phosphate import and control of parasite osmoregulation
Source: PLoS Pathog. 2020 Dec 31;16(12):e1009067. doi: 10.1371/journal.ppat.1009067 (PMC7817038; doi:10.1371/journal.ppat.1009067)
Supplement: S1 Table — (PDF) [file ppat.1009067.s007.pdf]

Name	Sequence 5' to 3' (restriction sites in bold)
FTgPiTUTR	TTT TGC CCT TCT GCG TCT TCT CTT ACC GAC ACA GT
RTgPiTUTR	ACG CTC ACA CGG CCT AAA ACC TCA CAC CCT TAA AT
FTgPiTpTUB	TAT **AGA TCT** ATG GAG GCG GTT GCT GAG
RTgPiTpTUB	TAT **CCT AGG** GGA GAC CAT GCG AGG GGA GTA GGC CGC
FPiTantiPep1	CCG **GGT ACC** CCT TCT ACC GCG TGG AGA GCT A
RPiTantiPep1	**CCT AGG** TTA TGC ATC TTC AGC TAA ACT TCG AG
FPiTantiPep2	ATA **GGA TCC** CGC GTG GAG AGC TAC CGG CGT
RPiTantiPep2	ATT **AAG CTT** TTA GCC CCC GGG TCG CGC GTG TGA
FqPCRTgPiT1	TTC AAG GAC ATC CAC GCT G
RqPCRTgPiT1	GTC TCC GTG TCA AAT ACC TCG
FqPCRTgPiT2	AAC ATG AGC TGC GAC CAG
RqPCRTgPiT2	AGG CAG GAA ATG ACG AAG G
Fα-actin	CCC GAT GGC AAC ATC ATC AC
Rα-actin	AAT GCC CTC GTA CAT AGT
Q5(F)129	AGC AGT TCG GGT TTT AGA GCT AGA AAT AGC
Q5(R)129	GAA CGT ATT CAA CTT GAC ATC CCC ATT TAC
F5'PiTKpnI	ATA **GGT ACC** CTT CCT TTC TCT CAC TTC GTC TTC TTC CTC
R5'PiTXhoI	GCG **CTG GAG** TAA CTA GGT ATA TAC AAA TAA ATT GGT AAT
F3'PiTBamHI	ATA **GGA TCC** GAG TTG TCA TTC AAC TCT CCA CGC TCA CCA
R3'PiTXbaI	GCG **TCT AGA** TTC GAT TTA AGA TGC ACC AGT AAA AGA
5'UPRTfwd	TGC CTG CAG GTT TTT CTG TTT TTC CTG C
5'UPRTrev	CCG CCT CCA TTT TAG AAG CCC TGT GGA AAG
PiTcDNAfwd	GGC TTC TAA AAT GGA GGC GGT TGCTGA G
PiTcDNArev	AAC TAG AGA CTT AGG AGA CCA TGC GAG G
3'UTRfwd	GGT CTC CTA AGT CTC TAG TTT TTT TGA CAG ACC G
3'UTRrev	CAG TGA ATT CTC GTC GCG ACG TCA ACT G
Puc19fwd	GTC GCG ACG AGA ATT CAC TGG CCG TCG TTT TAC
Puc19rev	AGC AGA AAA ACC TGC AGG CAT GCA AGC TTG
P1 for ΔTgPiT (Fig.S3)	AAC TGC TCA TTT TTC TGC AGA AAA A
P2 for ΔTgPiT (Fig.S3)	TGG TCG CTA GAC TGC ACA CGC GAG TTC CTA GAT CT
P3 for ΔTgPiT (Fig.S3)	ACG GAA AGT GCT TAC ATC GAA CAC GGT TAT CAA AC
P4 for ΔTgPiT (Fig.S3)	CGT TGT CAG ATC CAG CAA AAT GGC GTC CA
P5 for ΔTgPiT (Fig.S3)	CTG TGG GCT ATG CAG GGT TTA CTT CTC GA
P6 for ΔTgPiT (Fig.S3)	CAC CAC CTA AGT GTA AAC CTT GTT TAG GTC GAT A
P7 for ΔTgPiT (Fig.S3)&P2 in ΔTgPiT::*PiT* (Fig.S4)	CTT GCC AAT GTT GTA AGC GGC AAA AGG ACG
P8 for ΔTgPiT (Fig.S3)	ATG GAG GCG GTT GCT GAG CTC TCT GCG CCC T
P1 for ΔTgPiT::*PiT* (Fig.S4)	CCA GGA ACC GACGATGAACGC GAC TTG CGT CCA CT
P3 for ΔTgPiT::*PiT* (Fig.S4)	GAT CAA CAC GGC GTC GGT CAA CTG GAA GCT
P4 for ΔTgPiT::*PiT* (Fig.S4)	GCA ACA CTG GTG GAT AGC CGG ATG AAC CAG
P5 for ΔTgPiT::*PiT* (Fig.S4)	TCT ACG CCG ACC GCC TGA TTC GCC TCC TCA T
P6 for ΔTgPiT::*PiT* (Fig.S4)	CAG TAC AAG AGA AGC GGA TCT GCT TTT GAG ACT

S1 Table: List of primers used in this study
