# Supplementary material for: A single Na+-Pi cotransporter in Toxoplasma plays key roles in phosphate import and control of parasite osmoregulation
Source: PLoS Pathog. 2020 Dec 31;16(12):e1009067. doi: 10.1371/journal.ppat.1009067 (PMC7817038; doi:10.1371/journal.ppat.1009067)
Supplement: S2 Table — (PDF) [file ppat.1009067.s008.pdf]

| Gene_id | readcount_DeltaTgPiT | readcount_Parental | logFC | pval | padj | Annotation Blast swiss prot |
|---|---|---|---|---|---|---|
| TGGT1_216290A-t26_1 | 336.3203738 | 0.972197767 | 8.4344 | 3.76E-56 | 5.27E-53 | -//- |
| TGGT1_216290B-t26_1 | 616.7199557 | 6.840451961 | 6.4944 | 1.14E-80 | 4.78E-77 | -//- |
| TGGT1_216335-t26_1 | 3376.925715 | 49.15317091 | 6.1023 | 6.36E-96 | 5.34E-92 | -//- |
| TGGT1_221840-t26_1 | 45.13253831 | 1.046650121 | 5.4303 | 0.0004472 | 0.014072 | -//- |
| TGGT1_221310-t26_1 | 852.0608753 | 27.13845078 | 4.9725 | 4.01E-80 | 1.12E-76 | sp\|Q8H0S9\|PSA_ARATH Puromycin-sensitive aminopeptidase OS=Arabidopsis thaliana GN=MPA1 PE=2 SV=1//0 |
| TGGT1_243720-t26_1 | 43.08944634 | 1.45829665 | 4.885 | 3.10E-07 | 3.35E-05 | sp\|Q54H86\|PEX11_DICDI Peroxisomal membrane protein 11 homolog OS=Dictyostelium discoideum GN=pex11 PE=3 SV=1//4.36269e-26 |
| TGGT1_237585-t26_1 | 19.18173244 | 1.046650121 | 4.1959 | 0.0013243 | 0.031256 | -//- |
| TGGT1_233153-t26_1 | 68.03330962 | 3.774953953 | 4.1717 | 0.0006212 | 0.018314 | -//- |
| TGGT1_245530-t26_1 | 759.2211462 | 47.36199725 | 4.0027 | 2.69E-51 | 2.83E-48 | -//- |
| TGGT1_257720-t26_1 | 66.99716024 | 4.186600483 | 4.0002 | 5.44E-09 | 9.32E-07 | sp\|Q9XIE6\|ALA3_ARATH Phospholipid-transporting ATPase 3 OS=Arabidopsis thaliana GN=ALA3 PE=1 SV=2//4.2699e-16 |
| TGGT1_305100-t26_1 | 22.55596087 | 1.45829665 | 3.9512 | 0.0011512 | 0.028616 | -//- |
| TGGT1_304920-t26_1 | 40.07720883 | 2.728303832 | 3.8767 | 0.000438 | 0.013835 | -//- |
| TGGT1_215900-t26_1 | 35.68572953 | 2.504946771 | 3.8325 | 3.94E-05 | 0.0020678 | -//- |
| TGGT1_220560-t26_1 | 37.38401105 | 2.728303832 | 3.7763 | 0.0012584 | 0.030295 | -//- |
| TGGT1_280570-t26_1 | 440.518231 | 34.75849388 | 3.6638 | 0.0003487 | 0.011444 | -//- |
| TGGT1_204300-t26_1 | 25.92331711 | 2.093300241 | 3.6304 | 0.0006852 | 0.019783 | -//- |
| TGGT1_223258-t26_1 | 51.52759448 | 4.186600483 | 3.6215 | 0.0004962 | 0.01527 | -//- |
| TGGT1_239365-t26_1 | 32.9976859 | 2.728303832 | 3.5963 | 0.0001277 | 0.0052355 | -//- |
| TGGT1_203135-t26_1 | 36.36332409 | 3.065498008 | 3.5683 | 7.53E-05 | 0.0034879 | sp\|Q39565\|DYHB_CHLRE Dynein beta chain, flagellar outer arm OS=Chlamydomonas reinhardtii GN=ODA4 PE=3 SV=1//0 |
| TGGT1_295995-t26_1 | 46.45164844 | 4.037695775 | 3.5241 | 5.17E-06 | 0.000402 | -//- |
| TGGT1_293790-t26_1 | 186.5374478 | 16.3347554 | 3.5134 | 1.62E-14 | 5.93E-12 | -//- |
| TGGT1_280500-t26_1 | 36.7029804 | 3.214402716 | 3.5133 | 7.49E-05 | 0.0034879 | sp\|P55189\|YBAR_BACSU Putative sulfate transporter YbaR OS=Bacillus subtilis (strain 168) GN=ybaR PE=3 SV=2//1.36417e-25 |
| TGGT1_268800-t26_1 | 94.915464 | 8.561490433 | 3.4707 | 1.10E-10 | 2.58E-08 | -//- |
| TGGT1_248430-t26_1 | 54.19845765 | 5.307702957 | 3.3521 | 1.51E-06 | 0.0001361 | -//- |
| TGGT1_210478-t26_1 | 56.88478323 | 5.644897133 | 3.333 | 8.78E-07 | 8.38E-05 | -//- |
| TGGT1_219610-t26_1 | 34.67363281 | 3.477144538 | 3.3179 | 0.0001903 | 0.0070125 | -//- |
| TGGT1_284615-t26_1 | 24.57328213 | 2.653851479 | 3.2109 | 0.0017551 | 0.038806 | -//- |
| TGGT1_256700-t26_1 | 28.62338707 | 3.139950362 | 3.1884 | 0.0012446 | 0.030112 | -//- |
| TGGT1_252640-t26_1 | 328.515151 | 37.1539207 | 3.1444 | 4.09E-16 | 2.02E-13 | sp\|Q58623\|Y1226_METJA Putative cation-transporting ATPase MJ1226 OS=Methanocaldococcus jannaschii (strain ATCC 43067 / DSM 2661 / JAL-1 / JCM 10045 / NBRC 100440) GN=MJ1226 PE=3 SV=1//6.9619e-126 |
| TGGT1_225290-t26_1 | 229.587379 | 26.57358238 | 3.111 | 2.03E-19 | 1.22E-16 | sp\|Q27893\|NTP1_TOXGO Nucleoside-triphosphatase 1 OS=Toxoplasma gondii GN=NTP3 PE=1 SV=1//1.08395e-142 |
| TGGT1_268970-t26_1 | 70.03516846 | 8.522105673 | 3.0388 | 4.96E-07 | 5.03E-05 | -//- |
| TGGT1_233610-t26_1 | 48.1585202 | 5.868254194 | 3.0368 | 0.0008225 | 0.022221 | -//- |
| TGGT1_207210-t26_1 | 104.0294887 | 12.78315851 | 3.0247 | 8.28E-10 | 1.58E-07 | -//- |
| TGGT1_208020-t26_1 | 175.4267184 | 22.01903729 | 2.994 | 2.35E-11 | 6.32E-09 | -//- |
| TGGT1_295945-t26_1 | 69.02822588 | 8.859299848 | 2.9619 | 1.28E-06 | 0.0001185 | -//- |
| TGGT1_216140-t26_1 | 1482.689559 | 192.3036117 | 2.9468 | 1.16E-51 | 1.39E-48 | sp\|Q01484\|ANK2_HUMAN Ankyrin-2 OS=Homo sapiens GN=ANK2 PE=1 SV=4//1.72523e-07 |
| TGGT1_207130-t26_1 | 105.0518937 | 13.79474104 | 2.9289 | 4.53E-09 | 7.94E-07 | -//- |
| TGGT1_235950-t26_1 | 36.35129776 | 4.821604074 | 2.9144 | 0.0002369 | 0.008491 | sp\|Q45670\|THES_BACSJ Thermophilic serine proteinase OS=Bacillus sp. (strain AK1) PE=1 SV=1//5.91753e-30 |
| TGGT1_202020-t26_1 | 484.5922853 | 64.66031609 | 2.9058 | 8.43E-05 | 0.0037676 | sp\|Q9TRY0\|FKBP4_BOVIN Peptidyl-prolyl cis-trans isomerase FKBP4 OS=Bos taurus GN=FKBP4 PE=1 SV=4//1.36915e-06 |
| TGGT1_274170-t26_1 | 42.76525246 | 5.719349487 | 2.9025 | 0.0001812 | 0.0067355 | -//- |
| TGGT1_280580-t26_1 | 367.4347461 | 49.1882385 | 2.9011 | 0.0022883 | 0.045303 | -//- |
| TGGT1_219218-t26_1 | 120.5214594 | 16.63256482 | 2.8572 | 1.83E-10 | 3.88E-08 | -//- |
| TGGT1_220025-t26_1 | 58.92272106 | 8.224296257 | 2.8409 | 1.27E-05 | 0.0008321 | -//- |
| TGGT1_223262-t26_1 | 123.9077142 | 17.49524264 | 2.8242 | 6.30E-10 | 1.23E-07 | sp\|Q8IWG1\|WDR63_HUMAN WD repeat-containing protein 63 OS=Homo sapiens GN=WDR63 PE=2 SV=1//1.81223e-47 |
| TGGT1_207160-t26_1 | 186.1273516 | 26.57358238 | 2.8082 | 2.71E-08 | 3.92E-06 | -//- |
| TGGT1_248425-t26_1 | 66.31956568 | 9.608140554 | 2.7871 | 3.76E-06 | 0.0003101 | -//- |
| TGGT1_240200-t26_1 | 77.11640943 | 11.54821892 | 2.7394 | 0.0003527 | 0.011529 | -//- |
| TGGT1_268860-t26_1 | 106.3967745 | 16.03694599 | 2.73 | 1.56E-08 | 2.47E-06 | sp\|Q9UAE6\|ENO1_TOXGO Enolase 1 OS=Toxoplasma gondii GN=ENO1 PE=1 SV=1//0 |
| TGGT1_262970-t26_1 | 2103.927612 | 317.3410749 | 2.729 | 2.43E-56 | 4.08E-53 | -//- |
| TGGT1_305280-t26_1 | 48.48958626 | 7.326550844 | 2.7265 | 0.0001275 | 0.0052355 | -//- |
| TGGT1_253760-t26_1 | 89.89105937 | 13.79474104 | 2.7041 | 1.82E-07 | 2.18E-05 | -//- |
| TGGT1_222340-t26_1 | 30.98036464 | 4.896056428 | 2.6617 | 0.0024226 | 0.0469 | sp\|Q0V8R7\|NSUN4_BOVIN 5-methylcytosine rRNA methyltransferase NSUN4 OS=Bos taurus GN=NSUN4 PE=2 SV=2//3.23866e-19 |
| TGGT1_313050-t26_1 | 59.60718781 | 9.419851086 | 2.6617 | 0.0007467 | 0.020865 | sp\|Q9Y140\|DHRS7_DROME Dehydrogenase/reductase SDR family protein 7-like OS=Drosophila melanogaster GN=CG7601 PE=2 SV=1//2.64291e-15 |
| TGGT1_312435-t26_1 | 41.07040703 | 6.542642546 | 2.6502 | 0.0004367 | 0.013835 | -//- |
| TGGT1_243470-t26_1 | 50.50690753 | 8.224296257 | 2.6185 | 0.0001444 | 0.0056681 | -//- |
| TGGT1_232420-t26_1 | 53.19323311 | 8.710395141 | 2.6104 | 7.53E-05 | 0.0034879 | -//- |
| TGGT1_245770B-t26_1 | 48.82924257 | 8.224296257 | 2.5698 | 0.0003992 | 0.012851 | -//- |
| TGGT1_204290-t26_1 | 33.66669023 | 5.719349487 | 2.5574 | 0.0018417 | 0.039787 | -//- |
| TGGT1_224170-t26_1 | 92.24975498 | 15.7348194 | 2.5516 | 2.05E-07 | 2.33E-05 | -//- |
| TGGT1_291040-t26_1 | 47.13611519 | 8.149843903 | 2.532 | 0.0003102 | 0.010384 | sp\|Q27797\|LDH_TOXGO L-lactate dehydrogenase OS=Toxoplasma gondii PE=1 SV=1//0 |
| TGGT1_238915-t26_1 | 28.2699864 | 4.896056428 | 2.5296 | 0.0026106 | 0.049514 | -//- |
| TGGT1_207150-t26_1 | 217.1180245 | 38.45899548 | 2.4971 | 2.02E-14 | 7.06E-12 | -//- |
| TGGT1_316635-t26_1 | 125.9301896 | 22.76356083 | 2.4678 | 1.13E-08 | 1.83E-06 | -//- |
| TGGT1_203800-t26_1 | 33.99260215 | 6.20544837 | 2.4536 | 0.0015731 | 0.035339 | -//- |
| TGGT1_290970-t26_1 | 2576.472666 | 477.1197634 | 2.433 | 8.00E-38 | 6.72E-35 | sp\|B0K590\|BIOF_THEPX 8-amino-7-oxononanoate synthase OS=Thermoanaerobacter sp. (strain X514) GN=Teth514_0779 PE=3 SV=1//2.62341e-60 |
| TGGT1_245980-t26_1 | 391.7245504 | 72.78372673 | 2.4282 | 0.0004915 | 0.015239 | -//- |
| TGGT1_365480-t26_1 | 171.9683057 | 32.40676898 | 2.4078 | 0.0005834 | 0.017444 | -//- |
| TGGT1_293780-t26_1 | 1252.091801 | 238.5666226 | 2.3919 | 1.80E-15 | 7.97E-13 | -//- |
| TGGT1_290645-t26_1 | 39.05823991 | 7.475455552 | 2.3854 | 0.0014494 | 0.033734 | -//- |
| TGGT1_245770A-t26_1 | 258.9192855 | 50.64653515 | 2.354 | 3.34E-12 | 9.68E-10 | -//- |
| TGGT1_250000-t26_1 | 75.40266549 | 14.84139116 | 2.345 | 9.65E-06 | 0.0006759 | -//- |
| TGGT1_232170-t26_1 | 37.36511254 | 7.401003198 | 2.3359 | 0.0015425 | 0.035027 | sp\|O28078\|Y2205_ARCFU Uncharacterized protein AF_2205 OS=Archaeoglobus fulgidus (strain ATCC 49558 / VC-16 / DSM 4304 / JCM 9628 / NBRC 100126) GN=AF_2205 PE=4 SV=1//1.3111e-56 |
| TGGT1_227630-t26_1 | 871.105164 | 173.0430984 | 2.3317 | 2.94E-12 | 8.81E-10 | -//- |
| TGGT1_238440-t26_1 | 79.11826827 | 15.73913657 | 2.3297 | 1.16E-05 | 0.0007742 | -//- |
| TGGT1_286460-t26_1 | 161.9143423 | 32.37601855 | 2.3222 | 6.10E-10 | 1.22E-07 | -//- |
| TGGT1_285445-t26_1 | 60.92286185 | 12.18753968 | 2.3216 | 6.53E-05 | 0.0030991 | -//- |
| TGGT1_236880-t26_1 | 58.555576 | 11.77589315 | 2.314 | 0.002026 | 0.042134 | -//- |
| TGGT1_462970-t26_1 | 36.35301581 | 7.326550844 | 2.3109 | 0.0018762 | 0.039909 | -//- |
| TGGT1_304930-t26_1 | 202.7086608 | 41.00764417 | 2.3054 | 1.64E-06 | 0.0001453 | -//- |
| TGGT1_265320-t26_1 | 114.1229671 | 23.5517863 | 2.2767 | 2.03E-07 | 2.33E-05 | sp\|Q54PH5\|CWC2_DICDI Pre-mRNA-splicing factor cwc2 OS=Dictyostelium discoideum GN=cwc2 PE=3 SV=1//2.29242e-47 |
| TGGT1_202430-t26_1 | 329.5976876 | 68.1067102 | 2.2748 | 4.50E-16 | 2.10E-13 | -//- |
| TGGT1_265255-t26_1 | 58.24856259 | 12.22260727 | 2.2527 | 0.0001825 | 0.006756 | -//- |
| TGGT1_295720-t26_1 | 95.63257364 | 20.37245118 | 2.2309 | 3.66E-05 | 0.0019574 | sp\|Q9VWP4\|SUOX_DROME Probable sulfite oxidase, mitochondrial OS=Drosophila melanogaster GN=CG7280 PE=2 SV=1//1.15456e-98 |
| TGGT1_316560-t26_1 | 93.25669756 | 20.07464176 | 2.2158 | 4.01E-06 | 0.0003239 | -//- |
| TGGT1_215955-t26_1 | 45.78264412 | 9.905949969 | 2.2084 | 0.0008773 | 0.023436 | -//- |
| TGGT1_219450-t26_1 | 106.3658497 | 23.02630265 | 2.2077 | 3.88E-07 | 4.12E-05 | sp\|Q00664\|LIS1_EMENI Nuclear distribution protein nudF OS=Emericella nidulans (strain FGSC A4 / ATCC 38163 / CBS 112.46 / NRRL 194 / M139) GN=nudF PE=1 SV=1//8.45696e-14 |
| TGGT1_212275-t26_1 | 147.4259484 | 32.03882438 | 2.2021 | 5.85E-08 | 7.80E-06 | -//- |
| TGGT1_411080-t26_1 | 317.7200253 | 69.22781267 | 2.1983 | 3.09E-06 | 0.00026 | -//- |
| TGGT1_213700-t26_1 | 89.89449546 | 19.62361047 | 2.1956 | 5.51E-06 | 0.0004206 | -//- |
| TGGT1_295960-t26_1 | 1315.41631 | 289.532553 | 2.1837 | 1.13E-19 | 7.27E-17 | -//- |
| TGGT1_259020-t26_1 | 113.790183 | 25.1196029 | 2.1795 | 4.18E-07 | 4.39E-05 | sp\|Q84Q77\|HS17A_ORYSJ 17.9 kDa class I heat shock protein OS=Oryza sativa subsp. japonica GN=HSP17.9A PE=2 SV=1//8.60982e-17 |
| TGGT1_264150-t26_1 | 106.0347836 | 24.03788518 | 2.1412 | 1.21E-06 | 0.0001129 | -//- |
| TGGT1_268985-t26_1 | 74.39056877 | 16.96975899 | 2.1322 | 2.62E-05 | 0.0014928 | -//- |
| TGGT1_256800-t26_1 | 43.09975462 | 9.905949969 | 2.1213 | 0.0025705 | 0.048973 | -//- |
| TGGT1_305610-t26_1 | 244.1066978 | 56.18622951 | 2.1192 | 2.41E-11 | 6.32E-09 | -//- |
| TGGT1_209755A-t26_1 | 93.58776363 | 21.63814119 | 2.1127 | 1.93E-05 | 0.0011912 | -//- |
| TGGT1_209755B-t26_1 | 156.5434092 | 37.937829 | 2.0449 | 1.68E-08 | 2.60E-06 | sp\|Q27409\|FP1_MYTGA Adhesive plaque matrix protein OS=Mytilus galloprovincialis GN=FP1 PE=2 SV=1//2.72008e-06 |
| TGGT1_295020-t26_1 | 120.8679879 | 29.41572333 | 2.0388 | 7.07E-07 | 6.91E-05 | sp\|Q0EEE2\|PTHD3_MOUSE Patched domain-containing protein 3 OS=Mus musculus GN=Ptchd3 PE=1 SV=1//1.12177e-08 |
| TGGT1_207470-t26_1 | 111.779734 | 27.28303832 | 2.0346 | 5.24E-06 | 0.0004039 | -//- |
| TGGT1_293560-t26_1 | 68.33688694 | 16.70701717 | 2.0322 | 9.93E-05 | 0.0043017 | -//- |
| TGGT1_269680-t26_1 | 53.18464288 | 13.01083274 | 2.0313 | 0.0008866 | 0.023575 | sp\|Q9V9A7\|MCCB_DROME Probable methylcrotonoyl-CoA carboxylase beta chain, mitochondrial OS=Drosophila melanogaster GN=l(2)04524 PE=2 SV=1//0 |
| TGGT1_208380-t26_1 | 160.2349593 | 39.36537522 | 2.0252 | 2.62E-08 | 3.87E-06 | -//- |
| TGGT1_270670-t26_1 | 324.5698469 | 80.10596041 | 2.0185 | 3.62E-13 | 1.17E-10 | -//- |
| TGGT1_295950-t26_1 | 2018.726973 | 500.1369015 | 2.0131 | 9.98E-25 | 6.99E-22 | -//- |
| TGGT1_207350-t26_1 | 49.47934838 | 12.44596433 | 1.9911 | 0.0008185 | 0.022184 | -//- |
| TGGT1_293480-t26_1 | 2781.526278 | 701.3300332 | 1.9877 | 7.79E-35 | 5.95E-32 | sp\|Q39054\|CNX1_ARATH Molybdopterin biosynthesis protein CNX1 OS=Arabidopsis thaliana GN=CNX1 PE=1 SV=2//3.64665e-10 |
| TGGT1_364790-t26_1 | 43.0842922 | 10.87814774 | 1.9857 | 0.0024344 | 0.04702 | -//- |
| TGGT1_268190-t26_1 | 48.47927798 | 12.26199203 | 1.9832 | 0.0020538 | 0.042502 | -//- |
| TGGT1_269075-t26_1 | 104.7191095 | 26.91077655 | 1.9603 | 8.33E-05 | 0.0037434 | -//- |
| TGGT1_301000-t26_1 | 75.76981055 | 19.47470576 | 1.96 | 0.0026463 | 0.049964 | sp\|Q9Y7J3\|CARME_SCHPO Carnosine N-methyltransferase OS=Schizosaccharomyces pombe (strain 972 / ATCC 24843) GN=SPBC1778.07 PE=3 SV=1//1.29354e-43 |
| TGGT1_267130-t26_1 | 158.5521402 | 41.15654888 | 1.9458 | 5.44E-08 | 7.50E-06 | sp\|P13664\|P30_TOXGO Major surface antigen p30 OS=Toxoplasma gondii PE=1 SV=1//1.83234e-14 |
| TGGT1_225105-t26_1 | 354.4212876 | 92.43808763 | 1.9389 | 4.97E-07 | 5.03E-05 | -//- |
| TGGT1_270260-t26_1 | 253.4847847 | 66.20601659 | 1.9369 | 8.40E-11 | 2.14E-08 | -//- |
| TGGT1_219110-t26_1 | 98.94151629 | 25.8684436 | 1.9354 | 0.0020457 | 0.042439 | -//- |
| TGGT1_258910-t26_1 | 53.5139909 | 14.20638757 | 1.9134 | 0.0010147 | 0.02613 | -//- |
| TGGT1_321490-t26_1 | 215.7353466 | 57.6051414 | 1.905 | 8.77E-05 | 0.0038697 | sp\|P13664\|P30_TOXGO Major surface antigen p30 OS=Toxoplasma gondii PE=1 SV=1//4.85945e-17 |
| TGGT1_252395-t26_1 | 81.13215344 | 21.71691071 | 1.9015 | 7.75E-05 | 0.0035385 | -//- |
| TGGT1_205050-t26_1 | 91.88432797 | 25.1196029 | 1.871 | 2.05E-05 | 0.0012319 | -//- |
| TGGT1_267875-t26_1 | 77.76823329 | 21.37971654 | 1.8629 | 0.0001356 | 0.0054356 | sp\|P0DL09\|DRC1_CHLRE Dynein regulatory complex protein 1 OS=Chlamydomonas reinhardtii GN=DRC1 PE=1 SV=1//4.93913e-44 |
| TGGT1_321480-t26_1 | 164.6607995 | 45.26869701 | 1.8629 | 8.78E-05 | 0.0038697 | sp\|P13664\|P30_TOXGO Major surface antigen p30 OS=Toxoplasma gondii PE=1 SV=1//1.90152e-19 |
| TGGT1_266035-t26_1 | 76.41476222 | 21.08622429 | 1.8576 | 0.0002431 | 0.008547 | -//- |
| TGGT1_309030-t26_1 | 76.74926438 | 21.19574423 | 1.8564 | 0.0001623 | 0.0062279 | -//- |
| TGGT1_208730-t26_1 | 6062.769763 | 1683.378082 | 1.8486 | 1.62E-14 | 5.93E-12 | sp\|O00834\|MIC1_TOXGO Micronemal protein 1 OS=Toxoplasma gondii GN=MIC1 PE=1 SV=1//2.00961e-13 |
| TGGT1_268220-t26_1 | 175.4146921 | 48.96919861 | 1.8408 | 1.39E-07 | 1.74E-05 | -//- |
| TGGT1_288520-t26_1 | 104.3760172 | 29.30620338 | 1.8325 | 3.07E-05 | 0.0016629 | sp\|E1C6Q1\|PCH2_CHICK Pachytene checkpoint protein 2 homolog OS=Gallus gallus GN=TRIP13 PE=3 SV=1//1.09615e-105 |
| TGGT1_244590-t26_1 | 54.54498614 | 15.47639475 | 1.8174 | 0.0026235 | 0.049646 | sp\|Q9SEX2\|KTNA1_ARATH Katanin p60 ATPase-containing subunit A1 OS=Arabidopsis thaliana GN=AAA1 PE=1 SV=1//1.99647e-105 |
| TGGT1_200340-t26_1 | 114.8039978 | 32.7045784 | 1.8116 | 8.19E-06 | 0.0005935 | -//- |
| TGGT1_233210-t26_1 | 74.72850702 | 21.34464894 | 1.8078 | 0.0002365 | 0.008491 | -//- |
| TGGT1_409260-t26_1 | 366.0159892 | 105.1861785 | 1.799 | 2.15E-05 | 0.0012831 | -//- |
| TGGT1_292935-t26_1 | 160.5986682 | 47.58967148 | 1.7547 | 1.44E-06 | 0.0001317 | sp\|Q8BI79\|CCD40_MOUSE Coiled-coil domain-containing protein 40 OS=Mus musculus GN=Ccdc40 PE=1 SV=1//2.17375e-41 |
| TGGT1_225070-t26_1 | 51.16044943 | 15.21365293 | 1.7497 | 0.0022481 | 0.045146 | -//- |
| TGGT1_220420-t26_1 | 91.24109435 | 27.13845078 | 1.7493 | 0.0001468 | 0.0057379 | sp\|O60658\|PDE8A_HUMAN High affinity cAMP-specific and IBMX-insensitive 3',5'-cyclic phosphodiesterase 8A OS=Homo sapiens GN=PDE8A PE=1 SV=2//9.41128e-35 |
| TGGT1_203720-t26_1 | 456.5107671 | 137.8600065 | 1.7274 | 2.73E-13 | 9.18E-11 | -//- |
| TGGT1_297350-t26_1 | 103.6829602 | 31.51334073 | 1.7181 | 5.07E-05 | 0.00255 | -//- |
| TGGT1_220460-t26_1 | 73.70610202 | 22.57958853 | 1.7068 | 0.0008176 | 0.022184 | sp\|Q54DB1\|CM2A2_DICDI Charged multivesicular body protein 2a homolog 2 OS=Dictyostelium discoideum GN=chmp2a2 PE=3 SV=1//3.253e-15 |
| TGGT1_228175-t26_1 | 114.1384296 | 35.10000522 | 1.7012 | 4.08E-05 | 0.0021144 | -//- |
| TGGT1_201840-t26_1 | 1276.135241 | 393.8652183 | 1.696 | 0.0005179 | 0.015708 | sp\|Q9DEX3\|CATD_CLUHA Cathepsin D OS=Clupea harengus GN=ctsd PE=1 SV=1//1.84555e-65 |
| TGGT1_235650-t26_1 | 56.89165542 | 17.8675044 | 1.6709 | 0.0025279 | 0.048492 | sp\|Q75A82\|ANT1_ASHGO Peroxisomal adenine nucleotide transporter 1 OS=Ashbya gossypii (strain ATCC 10895 / CBS 109.51 / FGSC 9923 / NRRL Y-1056) GN=ANT1 PE=3 SV=1//1.37012e-20 |
| TGGT1_259270-t26_1 | 102.3535417 | 32.18772908 | 1.669 | 0.0001637 | 0.0062509 | -//- |
| TGGT1_310160-t26_1 | 122.8715648 | 38.80050682 | 1.663 | 1.63E-05 | 0.00103 | sp\|Q8CJG1\|AGO1_MOUSE Protein argonaute-1 OS=Mus musculus GN=Ago1 PE=1 SV=2//1.24542e-52 |

<table>
<tr><td>TGGT1_221220-t26_1</td><td>104.0346428</td><td>32.85780027</td><td>1.6628</td><td>0.0001086</td><td>0.004585</td><td>sp|Q3V0B4|CC108_MOUSE Coiled-coil domain-containing protein 108 OS=Mus musculus GN=Ccdc108 PE=1 SV=1//1.48037e-22</td></tr>
<tr><td>TGGT1_216710-t26_1</td><td>93.59119973</td><td>29.75723467</td><td>1.6531</td><td>0.000242</td><td>0.0085419</td><td>-//-</td></tr>
<tr><td>TGGT1_285940-t26_1</td><td>68.32142452</td><td>22.09348965</td><td>1.6287</td><td>0.0009669</td><td>0.025152</td><td>sp|Q5W6B9|HAP2A_ORYSJ Protein HAPLESS 2-A OS=Oryza sativa subsp. japonica GN=HAP2A PE=2 SV=1//1.81477e-11</td></tr>
<tr><td>TGGT1_266670-t26_1</td><td>61.59358422</td><td>20.00018941</td><td>1.6228</td><td>0.0018489</td><td>0.039787</td><td>-//-</td></tr>
<tr><td>TGGT1_281400-t26_1</td><td>766.2577179</td><td>248.9759401</td><td>1.6218</td><td>5.93E-13</td><td>1.85E-10</td><td>sp|Q8W4M5|PFPB1_ARATH Pyrophosphate--fructose 6-phosphate 1-phosphotransferase subunit beta 1 OS=Arabidopsis thaliana GN=PFP-BETA1 PE=1 SV=1//7.93018e-48</td></tr>
<tr><td>TGGT1_293258-t26_1</td><td>112.7901126</td><td>36.82104369</td><td>1.615</td><td>0.0001013</td><td>0.0043637</td><td>-//-</td></tr>
<tr><td>TGGT1_293290-t26_1</td><td>202.6141682</td><td>66.28046894</td><td>1.6121</td><td>0.0002629</td><td>0.0091669</td><td>-//-</td></tr>
<tr><td>TGGT1_218500-t26_1</td><td>105.0055064</td><td>34.35116451</td><td>1.612</td><td>0.0011238</td><td>0.028019</td><td>-//-</td></tr>
<tr><td>TGGT1_257670-t26_1</td><td>106.7312767</td><td>35.69562405</td><td>1.5802</td><td>0.0001443</td><td>0.0056681</td><td>-//-</td></tr>
<tr><td>TGGT1_264485-t26_1</td><td>384.7618547</td><td>130.9715355</td><td>1.5547</td><td>3.55E-09</td><td>6.48E-07</td><td>-//-</td></tr>
<tr><td>TGGT1_213020-t26_1</td><td>734.8414864</td><td>250.2281484</td><td>1.5542</td><td>3.84E-15</td><td>1.61E-12</td><td>-//-</td></tr>
<tr><td>TGGT1_312930-t26_1</td><td>170.0334507</td><td>58.05617268</td><td>1.5503</td><td>1.02E-05</td><td>0.0006898</td><td>sp|Q83A83|METC_COXBU Cystathionine beta-lyase OS=Coxiella burnetii (strain RSA 493 / Nine Mile phase I) GN=metC PE=1 SV=1//1.7987e-100</td></tr>
<tr><td>TGGT1_233920-t26_1</td><td>421.1354871</td><td>144.0740892</td><td>1.5475</td><td>9.27E-11</td><td>2.23E-08</td><td>sp|Q5NCP0|RNF43_MOUSE E3 ubiquitin-protein ligase RNF43 OS=Mus musculus GN=Rnf43 PE=2 SV=1//5.77659e-08</td></tr>
<tr><td>TGGT1_223480-t26_1</td><td>223.197477</td><td>76.55868068</td><td>1.5437</td><td>3.05E-07</td><td>3.35E-05</td><td>sp|Q9UPZ6|THS7A_HUMAN Thrombospondin type-1 domain-containing protein 7A OS=Homo sapiens GN=THSD7A PE=1 SV=4//1.60688e-14</td></tr>
<tr><td>TGGT1_227370-t26_1</td><td>115.8143765</td><td>39.73763699</td><td>1.5432</td><td>0.0001372</td><td>0.0054361</td><td>sp|P0A5F6|Y1866_MYCBO Putative serine esterase Mb1866c OS=Mycobacterium bovis (strain ATCC BAA-935 / AF2122/97) GN=Mb1866c PE=3 SV=1//9.50486e-08</td></tr>
<tr><td>TGGT1_210400-t26_1</td><td>236.3135013</td><td>81.27076481</td><td>1.5399</td><td>1.43E-07</td><td>1.77E-05</td><td>-//-</td></tr>
<tr><td>TGGT1_288640-t26_1</td><td>2346.647679</td><td>812.0467415</td><td>1.531</td><td>9.22E-06</td><td>0.0006597</td><td>sp|P73245|Y1917_SYNY3 Oxygen-independent coproporphyrinogen-III oxidase-like protein sll1917 OS=Synechocystis sp. (strain PCC 6803 / Kazusa) GN=sll1917 PE=2 SV=1//4.43595e-19</td></tr>
<tr><td>TGGT1_269050-t26_1</td><td>471.7145526</td><td>163.7548834</td><td>1.5264</td><td>3.01E-06</td><td>0.0002551</td><td>sp|Q0ANS1|KAD_MARMM Adenylate kinase OS=Maricaulis maris (strain MCS10) GN=adk PE=3 SV=1//4.42818e-07</td></tr>
<tr><td>TGGT1_208740-t26_1</td><td>6201.903431</td><td>2153.627173</td><td>1.5259</td><td>4.43E-08</td><td>6.20E-06</td><td>sp|O00834|MIC1_TOXGO Micronemal protein 1 OS=Toxoplasma gondii GN=MIC1 PE=1 SV=1//8.77474e-09</td></tr>
<tr><td>TGGT1_253460-t26_1</td><td>154.529524</td><td>53.8695722</td><td>1.5203</td><td>2.04E-05</td><td>0.0012319</td><td>-//-</td></tr>
<tr><td>TGGT1_215180-t26_1</td><td>60.91598966</td><td>21.27019659</td><td>1.518</td><td>0.0025813</td><td>0.049067</td><td>-//-</td></tr>
<tr><td>TGGT1_228160-t26_1</td><td>145.0620987</td><td>51.06681602</td><td>1.5062</td><td>0.0008923</td><td>0.023651</td><td>sp|P29288|PPA5  RAT Tartrate-resistant acid phosphatase type 5 OS=Rattus norvegicus GN=Acp5 PE=1 SV=1//1.89029e-16</td></tr>
<tr><td>TGGT1_254430-t26_1</td><td>89.23236332</td><td>32.14834432</td><td>1.4728</td><td>0.0016962</td><td>0.037903</td><td>sp|P03951|FA11_HUMAN Coagulation factor XI OS=Homo sapiens GN=F11 PE=1 SV=1//7.80133e-09</td></tr>
<tr><td>TGGT1_293280-t26_1</td><td>2513.65343</td><td>908.2905335</td><td>1.4686</td><td>6.99E-07</td><td>6.91E-05</td><td>sp|O80513|CCU41_ARATH Cyclin-U4-1 OS=Arabidopsis thaliana GN=CYCU4-1 PE=1 SV=1//3.50618e-12</td></tr>
<tr><td>TGGT1_223725-t26_1</td><td>323.5800848</td><td>117.1066592</td><td>1.4663</td><td>0.0001665</td><td>0.0063019</td><td>-//-</td></tr>
<tr><td>TGGT1_213255-t26_1</td><td>467.9422543</td><td>172.0579491</td><td>1.4434</td><td>1.45E-10</td><td>3.29E-08</td><td>-//-</td></tr>
<tr><td>TGGT1_307590-t26_1</td><td>99.6465996</td><td>36.71152374</td><td>1.4406</td><td>0.0007179</td><td>0.020477</td><td>-//-</td></tr>
<tr><td>TGGT1_281460-t26_1</td><td>124.887168</td><td>46.23657761</td><td>1.4335</td><td>8.33E-05</td><td>0.0037434</td><td>-//-</td></tr>
<tr><td>TGGT1_310480-t26_1</td><td>228.3026298</td><td>84.55098554</td><td>1.4331</td><td>0.0021311</td><td>0.043274</td><td>sp|Q9DAK2|PACRG_MOUSE Parkin coregulated gene protein homolog OS=Mus musculus GN=Pacrg PE=1 SV=1//2.76461e-78</td></tr>
<tr><td>TGGT1_215970-t26_1</td><td>798.4317753</td><td>298.6454301</td><td>1.4187</td><td>1.69E-10</td><td>3.73E-08</td><td>-//-</td></tr>
<tr><td>TGGT1_220585-t26_1</td><td>88.52556197</td><td>33.19499444</td><td>1.4151</td><td>0.0009357</td><td>0.024492</td><td>-//-</td></tr>
<tr><td>TGGT1_287960-t26_1</td><td>221.2110806</td><td>83.43420024</td><td>1.4067</td><td>4.55E-06</td><td>0.0003603</td><td>-//-</td></tr>
<tr><td>TGGT1_203290-t26_1</td><td>2497.79662</td><td>952.9555076</td><td>1.3902</td><td>2.31E-19</td><td>1.29E-16</td><td>-//-</td></tr>
<tr><td>TGGT1_249840-t26_1</td><td>340.6998267</td><td>131.3259983</td><td>1.3753</td><td>7.96E-08</td><td>1.03E-05</td><td>sp|Q9P2D7|DYH1_HUMAN Dynein heavy chain 1, axonemal OS=Homo sapiens GN=DNAH1 PE=2 SV=4//0</td></tr>
<tr><td>TGGT1_294400-t26_1</td><td>392.4330699</td><td>154.2648971</td><td>1.347</td><td>0.0014892</td><td>0.03428</td><td>sp|B6KV60|RON2_TOXGO Rhoptry neck protein 2 OS=Toxoplasma gondii GN=TGME49_100100 PE=1 SV=2//5.00644e-32</td></tr>
<tr><td>TGGT1_253330-t26_1</td><td>697.3870354</td><td>275.628292</td><td>1.3392</td><td>0.0007119</td><td>0.020415</td><td>-//-</td></tr>
<tr><td>TGGT1_205658-t26_1</td><td>82.12878774</td><td>32.78334792</td><td>1.3249</td><td>0.0022528</td><td>0.045146</td><td>sp|Q8CGD2|CRLD1_MOUSE Cysteine-rich secretory protein LCCL domain-containing 1 OS=Mus musculus GN=Crispld1 PE=2 SV=1//3.46047e-07</td></tr>
<tr><td>TGGT1_294805-t26_1</td><td>87.85483959</td><td>35.21384233</td><td>1.319</td><td>0.0019923</td><td>0.041537</td><td>-//-</td></tr>
<tr><td>TGGT1_206550-t26_1</td><td>391.1792455</td><td>156.8529306</td><td>1.3184</td><td>3.00E-08</td><td>4.27E-06</td><td>sp|Q4G0P3|HYDIN_HUMAN Hydrocephalus-inducing protein homolog OS=Homo sapiens GN=HYDIN PE=1 SV=3//7.6059e-34</td></tr>
<tr><td>TGGT1_319520-t26_1</td><td>310.0934793</td><td>125.3352727</td><td>1.3069</td><td>7.88E-07</td><td>7.61E-05</td><td>-//-</td></tr>
<tr><td>TGGT1_248240-t26_1</td><td>169.6697417</td><td>68.70664619</td><td>1.3042</td><td>6.26E-05</td><td>0.0030247</td><td>-//-</td></tr>
<tr><td>TGGT1_281930-t26_1</td><td>205.9987049</td><td>84.29256089</td><td>1.2892</td><td>7.90E-06</td><td>0.0005769</td><td>-//-</td></tr>
<tr><td>TGGT1_312330-t26_1</td><td>2124.730831</td><td>874.7809912</td><td>1.2803</td><td>1.85E-10</td><td>3.88E-08</td><td>-//-</td></tr>
<tr><td>TGGT1_272730-t26_1</td><td>183.4685147</td><td>77.30752139</td><td>1.2469</td><td>7.66E-05</td><td>0.0035152</td><td>-//-</td></tr>
<tr><td>TGGT1_203890-t26_1</td><td>92.56192253</td><td>39.17708575</td><td>1.2404</td><td>0.002359</td><td>0.046093</td><td>-//-</td></tr>
<tr><td>TGGT1_308965-t26_1</td><td>166.2869231</td><td>70.72981125</td><td>1.2333</td><td>0.000136</td><td>0.0054356</td><td>-//-</td></tr>
<tr><td>TGGT1_272370-t26_1</td><td>6010.120117</td><td>2569.366916</td><td>1.226</td><td>3.94E-09</td><td>7.04E-07</td><td>-//-</td></tr>
<tr><td>TGGT1_275870-t26_1</td><td>102.3380793</td><td>43.81040036</td><td>1.224</td><td>0.0021323</td><td>0.043274</td><td>sp|Q9D6T1|TBE_MOUSE Tubulin epsilon chain OS=Mus musculus GN=Tube1 PE=2 SV=1//4.57952e-35</td></tr>
<tr><td>TGGT1_220720-t26_1</td><td>269.6731781</td><td>116.213231</td><td>1.2144</td><td>7.16E-06</td><td>0.0005324</td><td>-//-</td></tr>
<tr><td>TGGT1_300130-t26_1</td><td>282.1322244</td><td>121.9805996</td><td>1.2097</td><td>9.81E-06</td><td>0.0006811</td><td>sp|B6KAM0|AMA1_TOXGO Apical membrane antigen 1 OS=Toxoplasma gondii GN=AMA1 PE=1 SV=1//1.61724e-53</td></tr>
<tr><td>TGGT1_209040-t26_1</td><td>549.9524968</td><td>245.5640833</td><td>1.1632</td><td>0.0023659</td><td>0.046122</td><td>sp|Q9CR92|CCD96_MOUSE Coiled-coil domain-containing protein 96 OS=Mus musculus GN=Ccdc96 PE=2 SV=1//1.01051e-10</td></tr>
<tr><td>TGGT1_215340-t26_1</td><td>112.7609058</td><td>50.69023708</td><td>1.1535</td><td>0.0018192</td><td>0.039599</td><td>-//-</td></tr>
<tr><td>TGGT1_236670A-t26_1</td><td>281.4666562</td><td>126.7585017</td><td>1.1509</td><td>2.04E-05</td><td>0.0012319</td><td>-//-</td></tr>
<tr><td>TGGT1_205265-t26_1</td><td>532.5343316</td><td>242.3847482</td><td>1.1356</td><td>5.58E-08</td><td>7.56E-06</td><td>sp|Q00975|CAC1B_HUMAN Voltage-dependent N-type calcium channel subunit alpha-1B OS=Homo sapiens GN=CACNA1B PE=1 SV=1//1.20848e-12</td></tr>
<tr><td>TGGT1_293500-t26_1</td><td>249.774336</td><td>114.1943831</td><td>1.1291</td><td>2.00E-05</td><td>0.0012264</td><td>-//-</td></tr>
<tr><td>TGGT1_219650-t26_1</td><td>498.6654288</td><td>230.8235778</td><td>1.1113</td><td>0.0002821</td><td>0.009635</td><td>sp|Q9LYG9|MSL10_ARATH Mechanosensitive ion channel protein 10 OS=Arabidopsis thaliana GN=MSL10 PE=1 SV=1//3.57272e-17</td></tr>
<tr><td>TGGT1_294785-t26_1</td><td>151.5104143</td><td>70.12987525</td><td>1.1113</td><td>0.0015984</td><td>0.035813</td><td>-//-</td></tr>
<tr><td>TGGT1_306620-t26_1</td><td>1229.966036</td><td>569.7321285</td><td>1.1103</td><td>8.38E-09</td><td>1.38E-06</td><td>-//-</td></tr>
<tr><td>TGGT1_291170-t26_1</td><td>175.4026658</td><td>82.6896767</td><td>1.0849</td><td>0.00075</td><td>0.020865</td><td>sp|Q54I39|IST1L_DICDI IST1-like protein OS=Dictyostelium discoideum GN=DDB_G0289029 PE=3 SV=1//1.23829e-17</td></tr>
<tr><td>TGGT1_212160-t26_1</td><td>205.0037886</td><td>98.35004375</td><td>1.0597</td><td>0.0002258</td><td>0.0082129</td><td>-//-</td></tr>
<tr><td>TGGT1_286740-t26_1</td><td>183.4960035</td><td>88.48347854</td><td>1.0523</td><td>0.0010964</td><td>0.027746</td><td>sp|P07207|NOTCH_DROME Neurogenic locus Notch protein OS=Drosophila melanogaster GN=N PE=1 SV=3//2.39046e-06</td></tr>
<tr><td>TGGT1_255290-t26_1</td><td>221.8388518</td><td>106.9859865</td><td>1.0521</td><td>0.0001764</td><td>0.006587</td><td>-//-</td></tr>
<tr><td>TGGT1_245440-t26_1</td><td>277.7647978</td><td>134.1244373</td><td>1.0503</td><td>0.0001074</td><td>0.0045565</td><td>-//-</td></tr>
<tr><td>TGGT1_267460-t26_1</td><td>142.7205835</td><td>69.15336032</td><td>1.0453</td><td>0.0012697</td><td>0.030479</td><td>-//-</td></tr>
<tr><td>TGGT1_297910-t26_1</td><td>127.2321192</td><td>62.27352359</td><td>1.0308</td><td>0.0019679</td><td>0.041335</td><td>-//-</td></tr>
<tr><td>TGGT1_315123-t26_1</td><td>247.4396931</td><td>121.6083378</td><td>1.0248</td><td>0.0002392</td><td>0.0085165</td><td>-//-</td></tr>
<tr><td>TGGT1_254640-t26_1</td><td>183.1305765</td><td>90.05129513</td><td>1.0241</td><td>0.000719</td><td>0.020477</td><td>sp|Q3T0X9|DPOD4_BOVIN DNA polymerase delta subunit 4 OS=Bos taurus GN=POLD4 PE=1 SV=1//1.19527e-10</td></tr>
<tr><td>TGGT1_233490-t26_1</td><td>3188.210651</td><td>1589.295074</td><td>1.0044</td><td>2.62E-08</td><td>3.87E-06</td><td>-//-</td></tr>
<tr><td>TGGT1_250100-t26_1</td><td>2119.712782</td><td>1058.373147</td><td>1.002</td><td>8.93E-11</td><td>2.21E-08</td><td>-//-</td></tr>
<tr><td>TGGT1_363340-t26_1</td><td>306.0141676</td><td>153.108727</td><td>0.99904</td><td>5.82E-05</td><td>0.0028611</td><td>-//-</td></tr>
<tr><td>TGGT1_250220-t26_1</td><td>3793.505992</td><td>1903.646694</td><td>0.99477</td><td>6.93E-12</td><td>1.94E-09</td><td>-//-</td></tr>
<tr><td>TGGT1_215500-t26_1</td><td>258.5263698</td><td>130.6866775</td><td>0.9842</td><td>0.0001655</td><td>0.0062922</td><td>-//-</td></tr>
<tr><td>TGGT1_222160-t26_1</td><td>845.7929546</td><td>430.0113434</td><td>0.97593</td><td>0.0012827</td><td>0.030704</td><td>sp|Q62148|AL1A2_MOUSE Retinal dehydrogenase 2 OS=Mus musculus GN=Aldh1a2 PE=1 SV=2//1.70784e-150</td></tr>
<tr><td>TGGT1_200350-t26_1</td><td>1347.962666</td><td>685.731167</td><td>0.97507</td><td>5.98E-09</td><td>1.00E-06</td><td>sp|P42779|BPRV_DICNO Extracellular basic protease OS=Dichelobacter nodosus GN=bprV PE=1 SV=1//1.2144e-28</td></tr>
<tr><td>TGGT1_272440-t26_1</td><td>200.3207583</td><td>102.5673947</td><td>0.96574</td><td>0.001017</td><td>0.02613</td><td>sp|Q1PDV2|SRC28_ARATH Serine/arginine-rich SC35-like splicing factor SCL28 OS=Arabidopsis thaliana GN=SCL28 PE=1 SV=1//6.82459e-22</td></tr>
<tr><td>TGGT1_223750-t26_1</td><td>460.231524</td><td>235.8728561</td><td>0.96435</td><td>1.56E-05</td><td>0.0010004</td><td>-//-</td></tr>
<tr><td>TGGT1_212810-t26_1</td><td>181.0995108</td><td>94.16344326</td><td>0.94354</td><td>0.0013799</td><td>0.032386</td><td>-//-</td></tr>
<tr><td>TGGT1_245428-t26_1</td><td>942.253794</td><td>492.0172777</td><td>0.93741</td><td>1.94E-07</td><td>2.26E-05</td><td>-//-</td></tr>
<tr><td>TGGT1_232590-t26_1</td><td>1107.302355</td><td>583.846795</td><td>0.92339</td><td>1.92E-07</td><td>2.26E-05</td><td>sp|Q09768|GSH1_SCHPO Glutamate--cysteine ligase OS=Schizosaccharomyces pombe (strain 972 / ATCC 24843) GN=gcs1 PE=3 SV=1//1.94145e-85</td></tr>
<tr><td>TGGT1_304730-t26_1</td><td>402.9194641</td><td>214.9177376</td><td>0.90671</td><td>0.0014689</td><td>0.034002</td><td>sp|Q8GYX0|MOB1B_ARATH MOB kinase activator-like 1B OS=Arabidopsis thaliana GN=MOB1B PE=2 SV=1//1.07775e-25</td></tr>
<tr><td>TGGT1_200320-t26_1</td><td>500.27609</td><td>267.2416093</td><td>0.90458</td><td>2.75E-05</td><td>0.0015282</td><td>sp|Q26997|HGXR_TOXGO Hypoxanthine-guanine-xanthine phosphoribosyltransferase OS=Toxoplasma gondii GN=HPRT PE=1 SV=1//5.93678e-164</td></tr>
<tr><td>TGGT1_239810-t26_1</td><td>723.7805804</td><td>388.201992</td><td>0.89874</td><td>2.27E-06</td><td>0.0001948</td><td>-//-</td></tr>
<tr><td>TGGT1_218740-t26_1</td><td>488.8291404</td><td>263.2303468</td><td>0.893</td><td>2.78E-05</td><td>0.0015391</td><td>-//-</td></tr>
<tr><td>TGGT1_235460-t26_1</td><td>200.963992</td><td>108.5581203</td><td>0.88847</td><td>0.0018674</td><td>0.039854</td><td>-//-</td></tr>
<tr><td>TGGT1_266870-t26_1</td><td>630.5479355</td><td>341.1815061</td><td>0.88607</td><td>9.26E-06</td><td>0.0006597</td><td>-//-</td></tr>
<tr><td>TGGT1_224710-t26_1</td><td>210.7057878</td><td>114.5402116</td><td>0.87938</td><td>0.001903</td><td>0.040173</td><td>sp|P93484|VSR1_PEA Vacuolar-sorting receptor 1 OS=Pisum sativum GN=BP80 PE=1 SV=1//1.39162e-20</td></tr>
<tr><td>TGGT1_215910-t26_1</td><td>7953.027456</td><td>4337.198355</td><td>0.87474</td><td>2.71E-10</td><td>5.55E-08</td><td>-//-</td></tr>
<tr><td>TGGT1_257360-t26_1</td><td>369.9372406</td><td>202.2354647</td><td>0.87124</td><td>0.0001057</td><td>0.0045317</td><td>-//-</td></tr>
<tr><td>TGGT1_273350-t26_1</td><td>398.6585564</td><td>218.3031611</td><td>0.86882</td><td>0.0012953</td><td>0.03083</td><td>sp|B4NJW0|MOC2B_DROWI Molybdopterin synthase catalytic subunit OS=Drosophila willistoni GN=Mocs2 PE=3 SV=1//1.773e-14</td></tr>
<tr><td>TGGT1_239090-t26_1</td><td>597.527915</td><td>330.3427431</td><td>0.85504</td><td>1.76E-05</td><td>0.0011025</td><td>-//-</td></tr>
<tr><td>TGGT1_254960-t26_1</td><td>290.8378709</td><td>160.8248083</td><td>0.85473</td><td>0.0025592</td><td>0.04887</td><td>-//-</td></tr>
<tr><td>TGGT1_214220-t26_1</td><td>14512.67401</td><td>8060.70308</td><td>0.84834</td><td>1.64E-07</td><td>2.00E-05</td><td>-//-</td></tr>
<tr><td>TGGT1_269417-t26_1</td><td>813.2443631</td><td>453.6505335</td><td>0.84211</td><td>4.25E-06</td><td>0.0003401</td><td>-//-</td></tr>
<tr><td>TGGT1_248140-t26_1</td><td>240.3687604</td><td>136.0645157</td><td>0.82096</td><td>0.002114</td><td>0.043217</td><td>-//-</td></tr>
<tr><td>TGGT1_244710-t26_1</td><td>671.2202726</td><td>382.9115577</td><td>0.80978</td><td>5.21E-05</td><td>0.0026042</td><td>-//-</td></tr>
<tr><td>TGGT1_214575-t26_1</td><td>541.9862945</td><td>309.81707</td><td>0.80684</td><td>7.17E-05</td><td>0.0033829</td><td>-//-</td></tr>
<tr><td>TGGT1_266910-t26_1</td><td>740.5812826</td><td>424.274195</td><td>0.80366</td><td>1.29E-05</td><td>0.0008398</td><td>sp|P51137|MSK1_MEDSA Glycogen synthase kinase-3 homolog MsK-1 OS=Medicago sativa GN=MSK-1 PE=2 SV=1//5.81887e-34</td></tr>
<tr><td>TGGT1_289050-t26_1</td><td>5560.864114</td><td>3191.086253</td><td>0.80126</td><td>7.64E-08</td><td>1.00E-05</td><td>-//-</td></tr>
<tr><td>TGGT1_233925-t26_1</td><td>403.9934105</td><td>234.8040899</td><td>0.78287</td><td>0.000795</td><td>0.0219</td><td>-//-</td></tr>
<tr><td>TGGT1_234180-t26_1</td><td>732.8310373</td><td>427.8694938</td><td>0.77631</td><td>2.63E-05</td><td>0.0014928</td><td>-//-</td></tr>
<tr><td>TGGT1_225790-t26_1</td><td>669.5958671</td><td>395.06456</td><td>0.7612</td><td>5.96E-05</td><td>0.002912</td><td>sp|Q7Y0F2|NRX12_ORYSJ Probable nucleoredoxin 1-2 OS=Oryza sativa subsp. japonica GN=Os03g0405900 PE=2 SV=1//2.42864e-09</td></tr>
<tr><td>TGGT1_225540-t26_1</td><td>2505.026814</td><td>1486.776759</td><td>0.75264</td><td>3.18E-05</td><td>0.0017117</td><td>-//-</td></tr>
<tr><td>TGGT1_237510-t26_1</td><td>756.6631571</td><td>449.2055084</td><td>0.75228</td><td>0.0008763</td><td>0.023436</td><td>-//-</td></tr>
<tr><td>TGGT1_236620-t26_1</td><td>820.6704145</td><td>505.2557847</td><td>0.69979</td><td>8.17E-05</td><td>0.0037126</td><td>-//-</td></tr>
<tr><td>TGGT1_239020-t26_1</td><td>1638.979214</td><td>1020.811897</td><td>0.68308</td><td>6.32E-05</td><td>0.0030335</td><td>sp|P08183|MDR1_HUMAN Multidrug resistance protein 1 OS=Homo sapiens GN=ABCB1 PE=1 SV=3//1.28154e-88</td></tr>
<tr><td>TGGT1_220230A-t26_1</td><td>853.4246547</td><td>536.0947371</td><td>0.67078</td><td>0.0003044</td><td>0.010229</td><td>-//-</td></tr>
<tr><td>TGGT1_236890-t26_1</td><td>2101.693817</td><td>1321.533359</td><td>0.66934</td><td>0.0020815</td><td>0.042938</td><td>-//-</td></tr>
<tr><td>TGGT1_266880-t26_1</td><td>1139.956949</td><td>718.9126797</td><td>0.66509</td><td>0.0001539</td><td>0.005959</td><td>sp|Q8ZKH4|DUSA_SALTY tRNA-dihydrouridine(20/20a) synthase OS=Salmonella typhimurium (strain LT2 / SGSC1412 / ATCC 700720) GN=dusA PE=3 SV=1//3.86666e-78</td></tr>
<tr><td>TGGT1_208450-t26_1</td><td>4005.96624</td><td>2535.22237</td><td>0.66004</td><td>7.56E-05</td><td>0.0034879</td><td>sp|O96790|DPGN_DIPMA Serine protease inhibitor dipetalogastin (Fragment) OS=Dipetalogaster maximus PE=1 SV=2//6.92367e-06</td></tr>
<tr><td>TGGT1_236010-t26_1</td><td>2955.645395</td><td>1884.530238</td><td>0.64927</td><td>1.02E-05</td><td>0.0006898</td><td>-//-</td></tr>
<tr><td>TGGT1_283810-t26_1</td><td>1166.101377</td><td>749.9005368</td><td>0.63692</td><td>0.0001254</td><td>0.0051914</td><td>sp|Q93XK2|STSYN_PEA Stachyose synthase OS=Pisum sativum GN=STS1 PE=1 SV=1//1.19629e-09</td></tr>
<tr><td>TGGT1_293810-t26_1</td><td>636.9464278</td><td>413.1419397</td><td>0.62453</td><td>0.001469</td><td>0.034002</td><td>sp|Q8NSH8|PRPB1_CORGL Probable 2-methylisocitrate lyase 1 OS=Corynebacterium glutamicum (strain ATCC 13032 / DSM 20300 / JCM 1318 / LMG 3730 / NCIMB 10025) GN=prpB1 PE=3 SV=1//2.19565e-70</td></tr>
<tr><td>TGGT1_230170-t26_1</td><td>4119.849197</td><td>2693.786645</td><td>0.61296</td><td>0.0001297</td><td>0.0052909</td><td>-//-</td></tr>
<tr><td>TGGT1_273320-t26_1</td><td>2009.605559</td><td>1316.685321</td><td>0.61</td><td>4.65E-05</td><td>0.0023918</td><td>-//-</td></tr>
<tr><td>TGGT1_213445-t26_1</td><td>3766.730356</td><td>2478.891553</td><td>0.60362</td><td>3.81E-05</td><td>0.0020115</td><td>-//-</td></tr>
<tr><td>TGGT1_278940-t26_1</td><td>1446.650429</td><td>952.7925909</td><td>0.60248</td><td>0.000416</td><td>0.013241</td><td>sp|O14326|PUB3_SCHPO E3 ubiquitin-protein ligase pub3 OS=Schizosaccharomyces pombe (strain 972 / ATCC 24843) GN=pub3 PE=2 SV=1//5.15235e-82</td></tr>
<tr><td>TGGT1_263220-t26_1</td><td>614.359542</td><td>404.996413</td><td>0.60117</td><td>0.0018956</td><td>0.040117</td><td>sp|Q9NQU5|PAK6_HUMAN Serine/threonine-protein kinase PAK 6 OS=Homo sapiens GN=PAK6 PE=1 SV=1//5.19295e-11</td></tr>
<tr><td>TGGT1_263270-t26_1</td><td>1324.888372</td><td>873.4138853</td><td>0.60113</td><td>0.0002231</td><td>0.0081496</td><td>sp|Q9ESM6|GDPD2_MOUSE Glycerophosphoinositol inositolphosphodiesterase GDPD2 OS=Mus musculus GN=Gdpd2 PE=1 SV=1//4.53144e-50</td></tr>
<tr><td>TGGT1_219230-t26_1</td><td>1144.926376</td><td>761.1158787</td><td>0.58907</td><td>0.0004749</td><td>0.014833</td><td>sp|Q9Z3R3|AACS_RHIME Acetoacetyl-coenzyme A synthetase OS=Rhizobium meliloti (strain 1021) GN=acsA2 PE=3 SV=2//2.03058e-131</td></tr>
<tr><td>TGGT1_203060-t26_1</td><td>3012.364045</td><td>2006.83455</td><td>0.58597</td><td>0.0001168</td><td>0.004884</td><td>-//-</td></tr>
<tr><td>TGGT1_262630-t26_1</td><td>604.2385748</td><td>402.8944784</td><td>0.58472</td><td>0.0018496</td><td>0.039787</td><td>-//-</td></tr>
<tr><td>TGGT1_311100-t26_1</td><td>5409.314184</td><td>3610.907318</td><td>0.58308</td><td>0.0002375</td><td>0.008491</td><td>-//-</td></tr>
<tr><td>TGGT1_269425-t26_1</td><td>1103.732269</td><td>738.9436195</td><td>0.57885</td><td>0.00059</td><td>0.017579</td><td>-//-</td></tr>
<tr><td>TGGT1_203870-t26_1</td><td>797.2020036</td><td>534.5619881</td><td>0.57659</td><td>0.0021504</td><td>0.043537</td><td>sp|Q9ULR0|ISY1_HUMAN Pre-mRNA-splicing factor ISY1 homolog OS=Homo sapiens GN=ISY1 PE=1 SV=3//2.06471e-54</td></tr>
<tr><td>TGGT1_269430-t26_1</td><td>1115.635702</td><td>751.2315145</td><td>0.57054</td><td>0.000916</td><td>0.024052</td><td>sp|Q653T6|SPS1_ORYSJ Solanesyl-diphosphate synthase 1, mitochondrial OS=Oryza sativa subsp. japonica GN=SPS1 PE=1 SV=1//1.91225e-40</td></tr>
<tr><td>TGGT1_316430-t26_1</td><td>1144.015644</td><td>772.8308012</td><td>0.56588</td><td>0.0011196</td><td>0.027997</td><td>sp|O14356|TOR1_SCHPO Phosphatidylinositol 3-kinase tor1 OS=Schizosaccharomyces pombe (strain 972 / ATCC 24843) GN=tor1 PE=1 SV=1//2.17642e-06</td></tr>
<tr><td>TGGT1_213280-t26_1</td><td>3688.333151</td><td>2507.024318</td><td>0.55699</td><td>0.0003321</td><td>0.01103</td><td>-//-</td></tr>
<tr><td>TGGT1_204540-t26_1</td><td>1423.399693</td><td>968.5101416</td><td>0.5555</td><td>0.000901</td><td>0.023785</td><td>sp|Q9UJK0|TSR3_HUMAN Ribosome biogenesis protein TSR3 homolog OS=Homo sapiens GN=TSR3 PE=1 SV=1//2.0456e-25</td></tr>
<tr><td>TGGT1_263740-t26_1</td><td>1367.380972</td><td>943.7978681</td><td>0.53487</td><td>0.0007302</td><td>0.020588</td><td>sp|Q9JI39|ABCBA_MOUSE ATP-binding cassette sub-family B member 10, mitochondrial OS=Mus musculus GN=Abcb10 PE=1 SV=1//2.70364e-51</td></tr>
<tr><td>TGGT1_208570-t26_1</td><td>3363.038805</td><td>2328.664352</td><td>0.53026</td><td>0.0002902</td><td>0.0098701</td><td>sp|Q8N2K1|UB2J2_HUMAN Ubiquitin-conjugating enzyme E2 J2 OS=Homo sapiens GN=UBE2J2 PE=1 SV=3//3.25291e-48</td></tr>
<tr><td>TGGT1_203790-t26_1</td><td>3172.121904</td><td>2199.050758</td><td>0.52857</td><td>0.0002739</td><td>0.0094303</td><td>-//-</td></tr>
<tr><td>TGGT1_237550-t26_1</td><td>1539.153938</td><td>1068.256981</td><td>0.52688</td><td>0.0012287</td><td>0.030098</td><td>-//-</td></tr>
<tr><td>TGGT1_205680-t26_1</td><td>993.6662793</td><td>690.2107295</td><td>0.52572</td><td>0.0012342</td><td>0.030112</td><td>-//-</td></tr>
<tr><td>TGGT1_304670-t26_1</td><td>1343.411409</td><td>933.9836391</td><td>0.52443</td><td>0.0013393</td><td>0.03152</td><td>sp|Q4R747|LRC46_MACFA Leucine-rich repeat-containing protein 46 OS=Macaca fascicularis GN=LRRC46 PE=2 SV=1//6.71104e-07</td></tr>
<tr><td>TGGT1_216770-t26_1</td><td>2549.801314</td><td>1803.014</td><td>0.49997</td><td>0.0011083</td><td>0.027964</td><td>-//-</td></tr>
<tr><td>TGGT1_300055-t26_1</td><td>1707.763477</td><td>1222.964275</td><td>0.48173</td><td>0.0019761</td><td>0.041404</td><td>-//-</td></tr>
<tr><td>TGGT1_204050-t26_1</td><td>12721.38737</td><td>9124.079152</td><td>0.47951</td><td>0.0004133</td><td>0.013204</td><td>sp|I3R794|HLY_HALMT Halolysin OS=Haloferax mediterranei (strain ATCC 33500 / DSM 1411 / JCM 8866 / NBRC 14739 / NCIMB 2177 / R-4) GN=hly PE=1 SV=1//5.72593e-29</td></tr>
<tr><td>TGGT1_208370-t26_1</td><td>5762.545732</td><td>4169.813635</td><td>0.46672</td><td>0.0007981</td><td>0.021914</td><td>-//-</td></tr>
<tr><td>TGGT1_286090-t26_1</td><td>9065.382738</td><td>6574.291614</td><td>0.46353</td><td>0.0011186</td><td>0.027997</td><td>sp|P42678|SUI1_ANOGA Protein translation factor SUI1 homolog OS=Anopheles gambiae GN=AGAP006459 PE=3 SV=1//6.64889e-33</td></tr>
<tr><td>TGGT1_268980-t26_1</td><td>2197.846442</td><td>1606.169325</td><td>0.45247</td><td>0.0023082</td><td>0.045418</td><td>-//-</td></tr>
<tr><td>TGGT1_239420-t26_1</td><td>11916.54748</td><td>8847.030888</td><td>0.4297</td><td>0.0022805</td><td>0.045297</td><td>sp|P53683|CDPK2_ORYSJ Calcium-dependent protein kinase isoform 2 OS=Oryza sativa subsp. japonica GN=CPK2 PE=2 SV=2//3.80474e-07</td></tr>
</table>

**S2_Table. SwissProt**
**KOPiTvsPar.DEG_up sort**
