# Supplementary material for: A single Na+-Pi cotransporter in Toxoplasma plays key roles in phosphate import and control of parasite osmoregulation
Source: PLoS Pathog. 2020 Dec 31;16(12):e1009067. doi: 10.1371/journal.ppat.1009067 (PMC7817038; doi:10.1371/journal.ppat.1009067)
Supplement: S3 Table — (PDF) [file ppat.1009067.s009.pdf]

Gene_id	readcount_DeltaTgPiT	readcount_Parental	logFC	pval	padj	Annotation Blast swiss prot
TGGT1_226755-t26_1	96.27237117	1315.37593	-3.7722	3.16E-70	6.63E-67	sp|Q61409|PDE3B MOUSE cGMP-inhibited 3',5'-cyclic phosphodiesterase B OS=Mus musculus GN=Pde3b PE=1 SV=2//7.90126e-32
TGGT1_226450-t26_1	16.49884295	59.51015217	-1.8508	0.00074724	0.020865	-//-
TGGT1_256650-t26_1	73.38706228	210.525579	-1.5204	1.14E-06	0.00010806	-//-
TGGT1_220330-t26_1	133.3081357	375.8655476	-1.4955	2.24E-09	4.19E-07	-//-
TGGT1_239350-t26_1	529.5805077	1471.387768	-1.4743	5.57E-18	2.92E-15	-//-
TGGT1_254890-t26_1	69.02135369	181.6660897	-1.3962	1.05E-05	0.00070616	-//-
TGGT1_411400-t26_1	99.9604852	238.9469884	-1.2573	2.92E-05	0.0016033	-//-
TGGT1_329800-t26_1	242.108275	548.4710965	-1.1798	0.00025114	0.0087918	-//-
TGGT1_308090-t26_1	12770.00014	27390.95681	-1.1009	1.51E-14	5.93E-12	sp|Q5Y808|ROP4 TOXGO Rhoptry protein 4 OS=Toxoplasma gondii GN=ROP4 PE=1 SV=1//3.33818e-17
TGGT1_261022-t26_1	2909.590616	6027.365613	-1.0507	5.06E-06	0.00039697	sp|Q39610|DYHA CHLRE Dynein alpha chain, flagellar outer arm OS=Chlamydomonas reinhardtii GN=ODA11 PE=3 SV=2//1.59303e-40
TGGT1_261075-t26_1	227.8513005	464.410527	-1.0273	0.00090305	0.023785	-//-
TGGT1_276910-t26_1	180.1320833	357.770369	-0.98998	1.60E-05	0.0010196	sp|P33948|ERD2 PLAFA ER lumen protein-retaining receptor OS=Plasmodium falciparum GN=ERD2 PE=2 SV=1//1.08534e-66
TGGT1_310740-t26_1	434.8174329	855.9186427	-0.97706	0.00033893	0.011167	-//-
TGGT1_255245-t26_1	211.7866064	415.8222245	-0.97336	6.74E-06	0.00050551	-//-
TGGT1_269930-t26_1	482.025706	940.4259263	-0.9642	4.56E-07	4.73E-05	sp|P14543|NID1_HUMAN Nidogen-1 OS=Homo sapiens GN=NID1 PE=1 SV=3//8.35226e-10
TGGT1_238040B-t26_1	93.92913798	180.5449872	-0.94271	0.0020893	0.042938	sp|P08003|PDIA4_MOUSE Protein disulfide-isomerase A4 OS=Mus musculus GN=Pdia4 PE=1 SV=3//4.30646e-13
TGGT1_267140-t26_1	221.5232481	425.6537221	-0.94222	2.43E-05	0.0014094	sp|P13664|P30 TOXGO Major surface antigen p30 OS=Toxoplasma gondii PE=1 SV=1//5.08545e-14
TGGT1_275650-t26_1	1767.331667	3390.169876	-0.93978	3.09E-07	3.35E-05	-//-
TGGT1_273970-t26_1	184.7807527	340.4369825	-0.88157	0.00065581	0.019237	sp|Q67UQ7|MRS2B ORYSJ Magnesium transporter MRS2-B OS=Oryza sativa subsp. japonica GN=MRS2-B PE=2 SV=1//1.15116e-08
TGGT1_225990-t26_1	615.1058585	1128.704794	-0.87576	3.11E-07	3.35E-05	sp|P71019|FABD_BACSU Malonyl CoA-acyl carrier protein transacylase OS=Bacillus subtilis (strain 168) GN=fabD PE=3 SV=2//2.21901e-44
TGGT1_313520-t26_1	132.6305411	241.2550114	-0.86315	0.0020902	0.042938	-//-
TGGT1_411390-t26_1	227.1994766	411.1187747	-0.8556	0.00038582	0.012516	-//-
TGGT1_209090-t26_1	596.8262678	1077.125446	-0.8518	3.51E-06	0.00029213	sp|Q3SZV5|POMP_BOVIN Proteasome maturation protein OS=Bos taurus GN=POMP PE=2 SV=1//8.48467e-10
TGGT1_262590-t26_1	297.2758782	536.3445274	-0.85136	3.05E-05	0.0016629	-//-
TGGT1_262040-t26_1	662.1990225	1190.58447	-0.84633	0.00033831	0.011167	sp|Q9UBQ5|EIF3K HUMAN Eukaryotic translation initiation factor 3 subunit K OS=Homo sapiens GN=EIF3K PE=1 SV=1//6.00193e-24
TGGT1_217182-t26_1	1956.384837	3513.354665	-0.84466	1.09E-07	1.38E-05	sp|Q9LQV2|RDR1 ARATH RNA-dependent RNA polymerase 1 OS=Arabidopsis thaliana GN=RDR1 PE=2 SV=1//2.84956e-37
TGGT1_293840-t26_1	3718.789511	6638.524076	-0.83603	1.70E-08	2.60E-06	-//-
TGGT1_217700-t26_1	767.7326534	1366.876508	-0.83221	2.72E-05	0.0015282	-//-
TGGT1_219240-t26_1	220.8164468	392.53318	-0.82997	0.00057851	0.017359	sp|Q3SZ85|PTRD1 BOVIN Putative peptidyl-tRNA hydrolase PTRHD1 OS=Bos taurus GN=PTRHD1 PE=2 SV=2//4.12669e-28
TGGT1_244180-t26_1	159.2297348	282.2712899	-0.82597	0.0012472	0.030112	sp|O08746|MATN2 MOUSE Matrilin-2 OS=Mus musculus GN=Matn2 PE=2 SV=2//6.8749e-19
TGGT1_295080-t26_1	195.8983542	344.7201515	-0.81532	0.0015627	0.03523	-//-
TGGT1_261070-t26_1	949.8769038	1663.096291	-0.80806	1.34E-05	0.00086921	sp|Q5VQL3|PPT3 ORYSJ Phosphoenolpyruvate/phosphate translocator 3, chloroplastic OS=Oryza sativa subsp. japonica GN=PPT3 PE=2 SV=1//7.07365e-62
TGGT1_320110-t26_1	691.8035814	1193.500533	-0.78676	5.88E-06	0.00044526	sp|O82797|PCNA TOBAC Proliferating cell nuclear antigen OS=Nicotiana tabacum GN=PCNA PE=2 SV=1//5.30552e-31
TGGT1_309265-t26_1	547.0330338	940.2198379	-0.78137	1.86E-05	0.0011594	sp|Q8BYK4|RDH12 MOUSE Retinol dehydrogenase 12 OS=Mus musculus GN=Rdh12 PE=2 SV=1//4.77271e-20
TGGT1_203930-t26_1	1690.077297	2896.325296	-0.77713	1.77E-06	0.00015474	-//-
TGGT1_201670-t26_1	361.2504927	618.9991365	-0.77694	5.24E-05	0.0026042	sp|Q98PI9|DNAJ MYCPU Chaperone protein DnaJ OS=Mycoplasma pulmonis (strain UAB CTIP) GN=dnaJ PE=3 SV=2//1.87466e-07
TGGT1_255310-t26_1	723.9059978	1235.76955	-0.77154	1.63E-06	0.00014525	-//-
TGGT1_272290-t26_1	880.960351	1502.017375	-0.76975	7.33E-06	0.00054011	sp|Q1XDM1|ODPB PYRYE Pyruvate dehydrogenase E1 component subunit beta OS=Pyropia yezoensis GN=pdhB PE=3 SV=1//7.89359e-138
TGGT1_309160-t26_1	464.83037	791.5297028	-0.76794	0.0017841	0.039242	-//-
TGGT1_251500-t26_1	1959.893073	3336.181089	-0.76742	6.01E-07	6.02E-05	sp|Q9C5Z2|EIF3H_ARATH Eukaryotic translation initiation factor 3 subunit H OS=Arabidopsis thaliana GN=TIF3H1 PE=1 SV=2//4.6141e-46
TGGT1_224800-t26_1	204.6830308	348.1228436	-0.7662	0.0013088	0.031065	-//-
TGGT1_409990-t26_1	1009.180514	1705.81634	-0.75728	9.65E-06	0.00067587	-//-
TGGT1_202030-t26_1	744.0792104	1253.895479	-0.75289	3.90E-06	0.00031851	-//-
TGGT1_266320-t26_1	821.9431373	1371.246551	-0.73838	0.0018051	0.039396	-//-
TGGT1_232390-t26_1	216.4558923	358.3659878	-0.72736	0.0024488	0.047189	sp|P56175|RCL1_DROME Probable RNA 3'-terminal phosphate cyclase-like protein OS=Drosophila melanogaster GN=Rtc1 PE=2 SV=3//3.70312e-55
TGGT1_245670-t26_1	1086.336439	1793.879537	-0.72361	1.18E-05	0.00077892	sp|Q1XDM0|ODPA PYRYE Pyruvate dehydrogenase E1 component subunit alpha OS=Pyropia yezoensis GN=pdhA PE=3 SV=1//6.47356e-125
TGGT1_253880-t26_1	998.0783752	1644.335359	-0.72028	2.54E-05	0.0014636	sp|Q5ZJR8|ELOV6 CHICK Elongation of very long chain fatty acids protein 6 OS=Gallus gallus GN=ELOVL6 PE=2 SV=1//1.6422e-25
TGGT1_254900-t26_1	757.0526368	1242.12822	-0.71435	6.47E-05	0.0030905	sp|Q9LST6|PSB2_ORYSJ Proteasome subunit beta type-2 OS=Oryza sativa subsp. japonica GN=PBD1 PE=2 SV=2//1.88735e-55
TGGT1_263470-t26_1	351.4399749	576.5116098	-0.71407	0.00074958	0.020865	sp|Q9JKB1|UCHL3 MOUSE Ubiquitin carboxyl-terminal hydrolase isozyme L3 OS=Mus musculus GN=Uchl3 PE=1 SV=2//5.98536e-48
TGGT1_239010-t26_1	947.9008157	1547.67992	-0.7073	4.70E-05	0.0023957	-//-
TGGT1_315620-t26_1	374.6683762	610.6043497	-0.70462	0.00067868	0.019663	sp|Q9SDS7|VATC ARATH V-type proton ATPase subunit C OS=Arabidopsis thaliana GN=VHA-C PE=1 SV=1//2.54657e-49
TGGT1_210420-t26_1	616.6254631	1001.810339	-0.70014	0.0003217	0.010726	-//-
TGGT1_239530-t26_1	920.2075761	1491.389018	-0.69663	0.0015348	0.035027	sp|Q54GT6|SPYA DICDI Serine--pyruvate aminotransferase OS=Dictyostelium discoideum GN=agxt PE=3 SV=1//4.51097e-101
TGGT1_247460-t26_1	1953.714491	3160.273733	-0.69383	2.01E-06	0.00017441	sp|P31008|PCNA PLAFK Proliferating cell nuclear antigen OS=Plasmodium falciparum (isolate K1 / Thailand) GN=PCNA PE=3 SV=1//2.39208e-73
TGGT1_273370-t26_1	713.6269703	1153.16296	-0.69235	0.00013498	0.0054356	sp|Q8H852|COPG1 ORYSJ Coatomer subunit gamma-1 OS=Oryza sativa subsp. japonica GN=Os03g0227000 PE=2 SV=2//6.84942e-14
TGGT1_222270-t26_1	610.0058598	985.0331865	-0.69135	9.83E-05	0.0042812	-//-
TGGT1_313080-t26_1	1474.069298	2370.626925	-0.68546	2.31E-05	0.0013567	-//-
TGGT1_217750-t26_1	592.8637832	951.3391416	-0.68226	0.00010718	0.0045565	-//-
TGGT1_203370-t26_1	951.0247262	1515.111295	-0.67187	5.47E-05	0.0027027	-//-
TGGT1_252280-t26_1	614.6562472	978.3594381	-0.67058	0.0003982	0.012851	-//-
TGGT1_287230-t26_1	315.1333464	500.0360158	-0.66607	0.00096215	0.025105	sp|Q09764|YA7B SCHPO Uncharacterized protein C24H6.11c OS=Schizosaccharomyces pombe (strain 972 / ATCC 24843) GN=SPAC24H6.11c PE=3 SV=1//1.14791e-41
TGGT1_232520-t26_1	449.8018253	713.1846958	-0.66499	0.00029278	0.0098792	sp|Q9VEB3|RPF2 DROME Ribosome production factor 2 homolog OS=Drosophila melanogaster GN=Non3 PE=2 SV=3//2.00734e-32
TGGT1_319720-t26_1	298.9415168	473.8039448	-0.66443	0.0018689	0.039854	-//-
TGGT1_266300-t26_1	1290.246181	2044.720573	-0.66426	2.34E-05	0.0013663	-//-
TGGT1_228190-t26_1	1413.436269	2236.103793	-0.66178	6.08E-05	0.002952	sp|Q54C49|EIF3F_DICDI Eukaryotic translation initiation factor 3 subunit F OS=Dictyostelium discoideum GN=eif3f PE=3 SV=1//4.17272e-37
TGGT1_294640-t26_1	3736.567949	5907.676903	-0.66088	9.95E-06	0.00068499	sp|P50648|RIR1_PLAF4 Ribonucleoside-diphosphate reductase large subunit OS=Plasmodium falciparum (isolate Dd2) GN=RNR1 PE=2 SV=1//0
TGGT1_289800-t26_1	542.6157838	857.0483795	-0.65945	0.00059227	0.017584	sp|Q90515|TMEDA TAKRU Transmembrane emp24 domain-containing protein 10 OS=Takifugu rubripes GN=tmed10 PE=3 SV=1//4.46934e-17
TGGT1_258800-t26_1	322.5490895	509.1186728	-0.65848	0.00087863	0.023436	sp|Q8RXG3|M2K5 ARATH Mitogen-activated protein kinase kinase 5 OS=Arabidopsis thaliana GN=MKK5 PE=1 SV=2//1.49872e-10
TGGT1_301480-t26_1	221.8903932	350.0672392	-0.65778	0.0022916	0.045303	-//-
TGGT1_258060-t26_1	800.1644178	1260.569227	-0.65571	0.00017235	0.0064645	-//-
TGGT1_411270-t26_1	413.3714974	650.8453542	-0.65488	0.0012069	0.029778	-//-
TGGT1_231890-t26_1	657.3957289	1031.208263	-0.6495	0.00046568	0.014599	sp|B0JXE2|FABH MICAN 3-oxoacyl-[acyl-carrier-protein] synthase 3 OS=Microcystis aeruginosa (strain NIES-843) GN=fabH PE=3 SV=1//7.29815e-115
TGGT1_214770-t26_1	752.0746194	1178.510237	-0.64802	0.00015668	0.0060388	sp|P22125|RAB1_DIPOM Ras-related protein ORAB-1 OS=Diplobatis ommata PE=2 SV=1//1.90156e-107
TGGT1_228290-t26_1	1713.486093	2678.666304	-0.64458	3.96E-05	0.0020685	sp|Q9WTL7|LYPA2 MOUSE Acyl-protein thioesterase 2 OS=Mus musculus GN=Lypla2 PE=1 SV=1//1.50701e-36
TGGT1_283710-t26_1	1112.609721	1737.456999	-0.64303	0.0017622	0.03886	sp|Q6YWS8|ASCL2 ORYSJ ASC1-like protein 2 OS=Oryza sativa subsp. japonica GN=Os02g0728300 PE=3 SV=2//5.84521e-20
TGGT1_217740-t26_1	1646.807973	2571.118705	-0.64272	3.77E-05	0.0020034	sp|P51831|FABG BACSU 3-oxoacyl-[acyl-carrier-protein] reductase FabG OS=Bacillus subtilis (strain 168) GN=fabG PE=3 SV=3//6.60156e-57
TGGT1_224880-t26_1	1261.70818	1968.355029	-0.64161	0.0017517	0.038806	sp|Q8J1G4|KIP1 ASHGO Kinesin-like protein KIP1 OS=Ashbya gossypii (strain ATCC 10895 / CBS 109.51 / FGSC 9923 / NRRL Y-1056) GN=KIP1 PE=3 SV=1//1.10656e-54
TGGT1_362090-t26_1	371.3044561	579.0381424	-0.64106	0.001744	0.038765	-//-
TGGT1_238040A-t26_1	537.2757755	836.6365436	-0.63894	0.00053863	0.016279	sp|Q7ZVE6|KDEL1 DANRE KDEL motif-containing protein 1 OS=Danio rerio GN=kdelc1 PE=2 SV=1//9.19987e-92
TGGT1_218760-t26_1	498.8624872	775.5585748	-0.63659	0.0010767	0.027413	sp|P93798|SNAA VITVI Alpha-soluble NSF attachment protein OS=Vitis vinifera PE=2 SV=1//2.61356e-42
TGGT1_312530-t26_1	766.51319	1189.213047	-0.63362	0.00028048	0.0096189	sp|Q86U06|RBM23 HUMAN Probable RNA-binding protein 23 OS=Homo sapiens GN=RBM23 PE=1 SV=1//8.26409e-28
TGGT1_310240-t26_1	928.0569511	1438.928663	-0.63271	0.00024158	0.0085419	-//-
TGGT1_299210-t26_1	1649.778977	2550.396639	-0.62845	9.60E-05	0.0041991	sp|Q6PEI7|PYRG1 DANRE CTP synthase 1 OS=Danio rerio GN=ctps1 PE=1 SV=1//0
TGGT1_240280-t26_1	1093.145027	1687.129859	-0.62609	8.80E-05	0.0038697	sp|Q9C9G4|ENDO2 ARATH Endonuclease 2 OS=Arabidopsis thaliana GN=ENDO2 PE=1 SV=1//5.90673e-06
TGGT1_280600-t26_1	2259.81748	3484.481163	-0.62474	4.67E-05	0.0023918	sp|Q86AS6|SYHC DICDI Histidine--tRNA ligase, cytoplasmic OS=Dictyostelium discoideum GN=hisS PE=3 SV=1//6.3986e-126
TGGT1_218362-t26_1	701.5470953	1081.005072	-0.62376	0.00038526	0.012516	-//-
TGGT1_249480-t26_1	1086.643452	1674.258236	-0.62364	0.00014948	0.0058144	sp|Q5ZKQ3|RPAP3 CHICK RNA polymerase II-associated protein 3 OS=Gallus gallus GN=RPAP3 PE=2 SV=1//3.67048e-07
TGGT1_201700-t26_1	1002.39426	1540.502274	-0.61995	0.00050718	0.01544	sp|Q9SRI1|SC13A ARATH Protein transport protein SEC13 homolog A OS=Arabidopsis thaliana GN=SEC13A PE=1 SV=1//1.40925e-49
TGGT1_314750-t26_1	485.7825419	746.554498	-0.61994	0.00082666	0.022262	sp|Q9UT32|RL1DB SCHPO Putative ribosome biogenesis protein C8F11.04 OS=Schizosaccharomyces pombe (strain 972 / ATCC 24843) GN=SPAC8F11.04 PE=3 SV=1//3.26033e-14
TGGT1_279420-t26_1	2336.995739	3574.449902	-0.61307	5.00E-05	0.0025317	-//-
TGGT1_237250-t26_1	1160.251626	1772.683263	-0.6115	0.00047754	0.01486	-//-
TGGT1_224850-t26_1	4463.342385	6819.156997	-0.61147	2.19E-05	0.0012952	sp|Q13310|PABP4_HUMAN Polyadenylate-binding protein 4 OS=Homo sapiens GN=PABPC4 PE=1 SV=1//1.28906e-14
TGGT1_300285-t26_1	375.3768956	573.2794082	-0.6109	0.0018052	0.039396	-//-
TGGT1_308050-t26_1	440.3051932	672.3653411	-0.61074	0.0024826	0.047732	sp|O18883|TXND9_BOVIN Thioredoxin domain-containing protein 9 OS=Bos taurus GN=TXNDC9 PE=2 SV=2//1.93608e-29
TGGT1_253730-t26_1	1197.148745	1827.004397	-0.60988	0.0001354	0.0054356	sp|Q7SZC2|XPO2_DANRE Exportin-2 OS=Danio rerio GN=cse1l PE=2 SV=1//2.72215e-86
TGGT1_229200-t26_1	460.4955863	702.4640872	-0.60924	0.0023248	0.045532	-//-
TGGT1_270550-t26_1	510.6714277	778.8738631	-0.60899	0.0013982	0.032723	sp|A5GJS5|PROA_SYNPW Gamma-glutamyl phosphate reductase OS=Synechococcus sp. (strain WH7803) GN=proA PE=3 SV=1//1.4382e-124
TGGT1_290660-t26_1	3622.182121	5505.964498	-0.60414	0.0001124	0.0047219	sp|Q6ICX4|PTBP3_ARATH Polypyrimidine tract-binding protein homolog 3 OS=Arabidopsis thaliana GN=At1g43190 PE=2 SV=1//6.292e-17
TGGT1_263260-t26_1	2184.075675	3308.285693	-0.59906	2.75E-05	0.0015282	sp|Q8N370|LAT4 HUMAN Large neutral amino acids transporter small subunit 4 OS=Homo sapiens GN=SLC43A2 PE=1 SV=1//9.44614e-08
TGGT1_209140-t26_1	572.9305802	867.0417333	-0.59774	0.0013144	0.031109	sp|Q9V464|ASF1 DROME Histone chaperone asf1 OS=Drosophila melanogaster GN=asf1 PE=1 SV=1//2.17425e-33
TGGT1_247470-t26_1	1391.803583	2090.532553	-0.58691	0.00012306	0.0051186	sp|Q21276|YZVL CAEEL Uncharacterized NOP5 family protein K07C5.4 OS=Caenorhabditis elegans GN=K07C5.4 PE=3 SV=1//1.63302e-114
TGGT1_235920-t26_1	1269.602741	1904.163013	-0.58478	0.0001365	0.0054356	sp|P0C6F1|DYH2 MOUSE Dynein heavy chain 2, axonemal OS=Mus musculus GN=Dnah2 PE=1 SV=1//2.66569e-55
TGGT1_210960-t26_1	519.1456549	776.5879563	-0.58101	0.0012001	0.029745	sp|Q54E21|RFC2 DICDI Probable replication factor C subunit 2 OS=Dictyostelium discoideum GN=rfc2 PE=3 SV=1//1.5664e-119
TGGT1_217820-t26_1	1526.068838	2281.898504	-0.58041	0.00017086	0.0064375	sp|Q54KZ8|EIF3M DICDI Eukaryotic translation initiation factor 3 subunit M OS=Dictyostelium discoideum GN=eif3m PE=1 SV=1//5.86982e-40
TGGT1_269650-t26_1	1326.406776	1977.590378	-0.57622	0.00027117	0.0093761	sp|Q8ND56|LS14A HUMAN Protein LSM14 homolog A OS=Homo sapiens GN=LSM14A PE=1 SV=3//1.18644e-24
TGGT1_300280-t26_1	593.4623476	881.9095577	-0.57147	0.0022701	0.045267	sp|Q55A45|RSMB DICDI Small nuclear ribonucleoprotein-associated protein B OS=Dictyostelium discoideum GN=snrpb PE=3 SV=1//1.43779e-21
TGGT1_305590-t26_1	1816.964606	2689.01412	-0.56555	0.00069487	0.019994	sp|Q54NL1|ABCC9 DICDI ABC transporter C family member 9 OS=Dictyostelium discoideum GN=abcC9 PE=3 SV=1//1.03404e-08
TGGT1_282210-t26_1	846.9282338	1247.326403	-0.55853	0.0018515	0.039787	-//-
TGGT1_203830-t26_1	1114.597835	1641.094523	-0.55814	0.00080714	0.022074	sp|Q1ACD5|TRIM5 SAGOE Tripartite motif-containing protein 5 OS=Saguinus oedipus GN=TRIM5 PE=3 SV=1//6.84962e-10
TGGT1_309200-t26_1	1623.313791	2389.365212	-0.55769	0.00026771	0.0092947	-//-
TGGT1_253380-t26_1	3473.26062	5101.442823	-0.55461	0.00072744	0.020579	-//-
TGGT1_226830-t26_1	4129.832721	6062.056168	-0.55372	0.00040666	0.013041	sp|Q566I3|HYOU1 XENLA Hypoxia up-regulated protein 1 (Fragment) OS=Xenopus laevis GN=hyou1 PE=1 SV=2//3.06047e-50
TGGT1_209160-t26_1	1124.30527	1649.962457	-0.5534	0.0012093	0.029778	sp|Q8K1Q0|NMT1 RAT Glycylpeptide N-tetradecanoyltransferase 1 OS=Rattus norvegicus GN=Nmt1 PE=1 SV=1//1.22048e-160
TGGT1_246500-t26_1	746.2867179	1093.455354	-0.55109	0.0021226	0.043274	sp|Q8RXF1|SF3A1_ARATH Probable splicing factor 3A subunit 1 OS=Arabidopsis thaliana GN=At1g14650 PE=1 SV=2//2.04999e-09
TGGT1_293730-t26_1	1018.946363	1492.391436	-0.55055	0.001564	0.03523	sp|Q9SB58|ZDH19_ARATH Protein S-acyltransferase 8 OS=Arabidopsis thaliana GN=PAT08 PE=1 SV=2//4.43506e-11
TGGT1_269190-t26_1	6340.665133	9281.191038	-0.54968	0.00020482	0.0075148	sp|Q9C136|G3P1_MUCCL Glyceraldehyde-3-phosphate dehydrogenase 1 OS=Mucor circinelloides f. lusitanicus GN=GPD1 PE=2 SV=1//2.10622e-135
TGGT1_238180-t26_1	1148.672903	1672.970961	-0.54244	0.00057739	0.017359	sp|Q55C75|PSMD6_DICDI 26S proteasome non-ATPase regulatory subunit 6 OS=Dictyostelium discoideum GN=psmD6 PE=1 SV=1//4.63746e-103
TGGT1_250955-t26_1	1971.614393	2868.170946	-0.54075	0.0006571	0.019237	-//-
TGGT1_297870-t26_1	1528.211577	2221.57801	-0.53974	0.00022978	0.0083215	-//-
TGGT1_297245-t26_1	1065.413474	1547.78944	-0.5388	0.0017196	0.038323	-//-
TGGT1_258150-t26_1	1262.152637	1832.241434	-0.53772	0.00049507	0.01527	sp|P34120|PSA7_DICDI Proteasome subunit alpha type-7 OS=Dictyostelium discoideum GN=psmA7 PE=3 SV=1//9.63248e-85
TGGT1_203450-t26_1	1015.959896	1472.737075	-0.53566	0.0015406	0.035027	-//-
TGGT1_311680-t26_1	870.3679548	1260.770998	-0.53461	0.00076372	0.021177	sp|E1BWM5|FUND1_CHICK FUN14 domain-containing protein 1 OS=Gallus gallus GN=FUNDC1 PE=3 SV=1//3.90515e-08
TGGT1_233100-t26_1	996.7489568	1443.693614	-0.53446	0.0018924	0.040117	sp|Q54Q31|PHB2 DICDI Prohibitin-2 OS=Dictyostelium discoideum GN=phbB PE=3 SV=1//3.26578e-97
TGGT1_243710-t26_1	2235.108989	3233.841899	-0.5329	0.00029155	0.0098775	sp|P47207|TCPB CAEEL T-complex protein 1 subunit beta OS=Caenorhabditis elegans GN=cct-2 PE=1 SV=2//0
TGGT1_272740-t26_1	1419.035702	2050.986992	-0.53141	0.00050592	0.01544	sp|Q9NW08|RPC2 HUMAN DNA-directed RNA polymerase III subunit RPC2 OS=Homo sapiens GN=POLR3B PE=1 SV=2//0
TGGT1_226430-t26_1	1825.78416	2632.093062	-0.52769	0.00067115	0.019512	-//-
TGGT1_215060-t26_1	1019.327252	1465.857238	-0.52413	0.0019646	0.041335	sp|Q4P0I7|SAR1 USTMA Small COPII coat GTPase SAR1 OS=Ustilago maydis (strain 521 / FGSC 9021) GN=SAR1 PE=3 SV=1//8.40674e-89
TGGT1_287500-t26_1	1946.576554	2792.146914	-0.52044	0.00050407	0.01544	sp|Q6EE31|TCPQ CHICK T-complex protein 1 subunit theta OS=Gallus gallus GN=CCT8 PE=1 SV=3//3.32778e-142
TGGT1_315360-t26_1	1235.510492	1771.316687	-0.51971	0.0010058	0.026081	-//-
TGGT1_266366-t26_1	3688.54292	5286.010964	-0.51913	0.00101	0.026112	sp|Q9SQN2|FBT3 ARATH Probable folate-biopterin transporter 3 OS=Arabidopsis thaliana GN=At1g79710 PE=2 SV=1//1.67411e-14
TGGT1_290600-t26_1	1381.534864	1977.975591	-0.51775	0.0015414	0.035027	sp|Q8GTQ9|SUCA1 SOLLC Succinyl-CoA ligase [ADP-forming] subunit alpha-1, mitochondrial OS=Solanum lycopersicum GN=SCOA PE=1 SV=1//3.47574e-139
TGGT1_227650-t26_1	1086.36221	1554.384419	-0.51684	0.0014825	0.034221	sp|Q9FJJ5|EB1B ARATH Microtubule-associated protein RP/EB family member 1B OS=Arabidopsis thaliana GN=EB1B PE=1 SV=1//7.42101e-39
TGGT1_219520-t26_1	1564.778832	2234.548928	-0.51402	0.00072215	0.020498	sp|Q9URX7|ANM1 SCHPO Protein arginine N-methyltransferase 1 OS=Schizosaccharomyces pombe (strain 972 / ATCC 24843) GN=rmt1 PE=1 SV=2//7.81647e-145
TGGT1_269980-t26_1	4566.659402	6508.400146	-0.51116	0.00059695	0.01766	sp|Q54XK2|SC61A DICDI Protein transport protein Sec61 subunit alpha OS=Dictyostelium discoideum GN=sec61a PE=3 SV=1//0
TGGT1_275610-t26_1	2387.34974	3394.214085	-0.50767	0.0015388	0.035027	sp|O80673|CAMK1 ARATH CDPK-related kinase 1 OS=Arabidopsis thaliana GN=CRK1 PE=1 SV=1//1.44847e-16
TGGT1_256000-t26_1	1468.523641	2087.611642	-0.50749	0.0010485	0.026776	sp|P33948|ERD2 PLAFA ER lumen protein-retaining receptor OS=Plasmodium falciparum GN=ERD2 PE=2 SV=1//1.68595e-92

TGGT1 229180-t26 1	1968.036234	2792.63733	-0.50487	0.0010237	0.026224	sp|O00410|IPO5 HUMAN Importin-5 OS=Homo sapiens GN=IPO5 PE=1 SV=4//3.46538e-06
TGGT1 206610-t26 1	1614.841282	2286.272864	-0.50161	0.0015576	0.03523	sp|Q9SQI8|ODP24 ARATH Dihydrolipoyllysine-residue acetyltransferase component 4 of pyruvate dehydrogenase complex, chloroplastic OS=Arabidopsis thaliana GN=LTA2 PE=2 SV=1//1.48784e-13
TGGT1 310750-t26 1	2357.76408	3331.157714	-0.4986	0.0012396	0.030112	sp|Q9Y3B3|TMED7 HUMAN Transmembrane emp24 domain-containing protein 7 OS=Homo sapiens GN=TMED7 PE=1 SV=2//8.22477e-11
TGGT1 288920-t26 1	2134.928594	3016.120344	-0.49851	0.0012469	0.030112	sp|Q8T685|ABCGC DICDI ABC transporter G family member 12 OS=Dictyostelium discoideum GN=abcG12 PE=3 SV=1//1.01377e-49
TGGT1 262380-t26 1	1127.139348	1589.204414	-0.49564	0.0018677	0.039854	sp|Q748X8|EFTU GEOSL Elongation factor Tu OS=Geobacter sulfurreducens (strain ATCC 51573 / DSM 12127 / PCA) GN=tuf1 PE=3 SV=1//1.08697e-172
TGGT1 257550-t26 1	1752.865091	2460.813643	-0.48942	0.0017938	0.039352	-//-
TGGT1 203600-t26 1	1464.754779	2048.902856	-0.48419	0.0022981	0.045325	-//-
TGGT1 254820-t26 1	1324.718803	1850.652221	-0.48235	0.0023918	0.046518	-//-
TGGT1 243920-t26 1	2316.187883	3226.773242	-0.47834	0.0012121	0.029778	sp|P55862|MCM5A XENLA DNA replication licensing factor mcm5-A OS=Xenopus laevis GN=mcm5-a PE=1 SV=2//1.10936e-149
TGGT1 214970-t26 1	4346.055993	6049.141373	-0.47702	0.0011121	0.027975	sp|P97310|MCM2 MOUSE DNA replication licensing factor MCM2 OS=Mus musculus GN=Mcm2 PE=1 SV=3//3.39335e-159
TGGT1 270510-t26 1	6619.576552	9195.966194	-0.47426	0.00077432	0.021401	sp|Q6MEC9|SYN PARUW Asparagine--tRNA ligase OS=Protochlamydia amoebophila (strain UWE25) GN=asnS PE=3 SV=1//3.35879e-172
TGGT1 363030-t26 1	13361.94874	18530.50723	-0.47177	0.00066371	0.019363	sp|Q5Y808|ROP4 TOXGO Rhoptry protein 4 OS=Toxoplasma gondii GN=ROP4 PE=1 SV=1//1.96698e-81
TGGT1 273460-t26 1	3643.800895	5049.942245	-0.47082	0.00080917	0.022074	sp|Q6P878|EIF3L XENTR Eukaryotic translation initiation factor 3 subunit L OS=Xenopus tropicalis GN=eif3l PE=2 SV=1//1.61084e-96
TGGT1 243200-t26 1	6124.62741	8480.15355	-0.46947	0.0023144	0.045433	-//-
TGGT1_218240-t26_1	3878.562833	5349.939708	-0.464	0.0025364	0.048543	-//-
TGGT1_213570-t26_1	2179.030654	2996.12985	-0.45941	0.0010934	0.027746	-//-
TGGT1_411430-t26_1	3125.001252	4290.479996	-0.45728	0.0022568	0.045146	sp|Q5Y808|ROP4_TOXGO Rhoptry protein 4 OS=Toxoplasma gondii GN=ROP4 PE=1 SV=1//2.47048e-13
TGGT1_251690-t26_1	3025.946861	4148.675075	-0.45526	0.0014172	0.033076	sp|O81983|SYS HELAN Serine--tRNA ligase OS=Helianthus annuus PE=2 SV=1//1.37664e-163
TGGT1_314400-t26_1	2123.387669	2886.432828	-0.44292	0.0021828	0.044087	sp|P21839|ODBB_BOVIN 2-oxoisovalerate dehydrogenase subunit beta, mitochondrial OS=Bos taurus GN=BCKDHB PE=1 SV=2//1.15377e-160
TGGT1_226960-t26_1	9838.558483	13352.70477	-0.44061	0.0018425	0.039787	sp|F4JGR5|PFPB2 ARATH Pyrophosphate--fructose 6-phosphate 1-phosphotransferase subunit beta 2 OS=Arabidopsis thaliana GN=PFP-BETA2 PE=3 SV=1//6.68423e-26
TGGT1_318310-t26_1	1879.042679	2549.543125	-0.44024	0.0019844	0.041475	sp|P23254|TKT1_YEAST Transketolase 1 OS=Saccharomyces cerevisiae (strain ATCC 204508 / S288c) GN=TKL1 PE=1 SV=4//0
TGGT1_311240-t26_1	3819.110786	5143.509539	-0.42952	0.002413	0.046821	sp|P42825|DNAJ2_ARATH Chaperone protein dnaJ 2 OS=Arabidopsis thaliana GN=ATJ2 PE=1 SV=2//3.27851e-127
TGGT1_225050-t26_1	4222.05482	5661.093664	-0.42314	0.0021116	0.043217	sp|O23255|SAHH1_ARATH Adenosylhomocysteinase 1 OS=Arabidopsis thaliana GN=SAHH1 PE=1 SV=1//0
TGGT1_261950-t26_1	5913.201426	7794.848041	-0.39858	0.0022382	0.045097	sp|P56480|ATPB_MOUSE ATP synthase subunit beta, mitochondrial OS=Mus musculus GN=Atp5b PE=1 SV=2//0
TGGT1 290200-t26 1	5634.519024	7396.337087	-0.39252	0.0022736	0.045267	sp|Q8T882|TADH ARAIR Tauropine dehydrogenase OS=Arabella iricolor GN=tadh PE=1 SV=1//1.61004e-56
TGGT1 240210-t26 1	0		#NAME?	3.27E-45	3.05E-42	sp|Q61609|S20A1 MOUSE Sodium-dependent phosphate transporter 1 OS=Mus musculus GN=Slc20a1 PE=1 SV=1//8.52597e-36

S3 Table. SwissProt
KOPiTvsPar.DEG_down sort
