# Supplementary material for: A single Na+-Pi cotransporter in Toxoplasma plays key roles in phosphate import and control of parasite osmoregulation
Source: PLoS Pathog. 2020 Dec 31;16(12):e1009067. doi: 10.1371/journal.ppat.1009067 (PMC7817038; doi:10.1371/journal.ppat.1009067)
Supplement: S4 Table — (PDF) [file ppat.1009067.s010.pdf]

Gene_id	readcount_DeltaTgPiT	readcount_Parental	logFC	pval	padj	Annotation ToxoDB
TGGT1_216290A-t26_1	336.3203738	0.972197767	8.4344	3.76E-56	5.27E-53	hypothetical protein
TGGT1_216290B-t26_1	616.7199557	6.840451961	6.4944	1.14E-80	4.78E-77	hypothetical protein
TGGT1_216335-t26_1	3376.925715	49.15317091	6.1023	6.36E-96	5.34E-92	hypothetical protein
TGGT1_221840-t26_1	45.13253831	1.046650121	5.4303	0.000447	0.014072	hypothetical protein
TGGT1_221310-t26_1	852.0608753	27.13845078	4.9725	4.01E-80	1.12E-76	membrane alanylaminopeptidase N/metallopeptidase
TGGT1_243720-t26_1	43.08944634	1.45829665	4.885	3.10E-07	3.35E-05	peroxisomal biogenesis factor PEX11
TGGT1_237585-t26_1	19.18173244	1.046650121	4.1959	0.001324	0.031256	hypothetical protein
TGGT1_233153-t26_1	68.03330962	3.774953953	4.1717	0.000621	0.018314	hypothetical protein
TGGT1_245530-t26_1	759.2211462	47.36199725	4.0027	2.69E-51	2.83E-48	hypothetical protein
TGGT1_257720-t26_1	66.99716024	4.186600483	4.0002	5.44E-09	9.32E-07	putative proton ATPase
TGGT1_305100-t26_1	22.55596087	1.45829665	3.9512	0.001151	0.028616	hypothetical protein
TGGT1_304920-t26_1	40.07720883	2.728303832	3.8767	0.000438	0.013835	hypothetical protein
TGGT1_215900-t26_1	35.68572953	2.504946771	3.8325	3.94E-05	0.002068	hypothetical protein
TGGT1_220560-t26_1	37.38401105	2.728303832	3.7763	0.001258	0.030295	hypothetical protein
TGGT1_280570-t26_1	440.518231	34.75849388	3.6638	0.000349	0.011444	SAG4
TGGT1_204300-t26_1	25.92331711	2.093300241	3.6304	0.000685	0.019783	hypothetical protein
TGGT1_223258-t26_1	51.52759448	4.186600483	3.6215	0.000496	0.01527	hypothetical protein
TGGT1_239365-t26_1	32.9976859	2.728303832	3.5963	0.000128	0.005236	hypothetical protein
TGGT1_203135-t26_1	36.36332409	3.065498008	3.5683	7.53E-05	0.003488	cytoplasmic dynein heavy chain family protein
TGGT1_295995-t26_1	46.45164844	4.037695775	3.5241	5.17E-06	0.000402	hypothetical protein
TGGT1_293790-t26_1	186.5374478	16.3347554	3.5134	1.62E-14	5.93E-12	hypothetical protein
TGGT1_280500-t26_1	36.7029804	3.214402716	3.5133	7.49E-05	0.003488	inorganic anion transporter, sulfate permease (SulP) family protein
TGGT1_268800-t26_1	94.915464	8.561490433	3.4707	1.10E-10	2.58E-08	hypothetical protein
TGGT1_248430-t26_1	54.19845765	5.307702957	3.3521	1.51E-06	0.000136	hypothetical protein
TGGT1_210478-t26_1	56.88478323	5.644897133	3.333	8.78E-07	8.38E-05	hypothetical protein
TGGT1_219610-t26_1	34.67363281	3.477144538	3.3179	0.00019	0.007013	hypothetical protein
TGGT1_284615-t26_1	24.57328213	2.653851479	3.2109	0.001755	0.038806	hypothetical protein
TGGT1_256700-t26_1	28.62338707	3.139950362	3.1884	0.001245	0.030112	hypothetical protein
TGGT1_252640-t26_1	328.515151	37.1539207	3.1444	4.09E-16	2.02E-13	P-type ATPase Plasma Membrane A1
TGGT1_225290-t26_1	229.587379	26.57358238	3.111	2.03E-19	1.22E-16	nucleotide triphosphate (NTPase)
TGGT1_268970-t26_1	70.03516846	8.522105673	3.0388	4.96E-07	5.03E-05	hypothetical protein
TGGT1_233610-t26_1	48.1585202	5.868254194	3.0368	0.000823	0.022221	hypothetical protein
TGGT1_207210-t26_1	104.0294887	12.78315851	3.0247	8.28E-10	1.58E-07	hypothetical protein
TGGT1_208020-t26_1	175.4267184	22.01903729	2.994	2.35E-11	6.32E-09	AP2 domain transcription factor AP2Ib-1
TGGT1_295945-t26_1	69.02822588	8.859299848	2.9619	1.28E-06	0.000119	hypothetical protein
TGGT1_216140-t26_1	1482.689559	192.3036117	2.9468	1.16E-51	1.39E-48	tetratricopeptide repeat-containing protein
TGGT1_207130-t26_1	105.0518937	13.79474104	2.9289	4.53E-09	7.94E-07	SAG2Y-related sequence
TGGT1_235950-t26_1	36.35129776	4.821604074	2.9144	0.000237	0.008491	subtilisin SUB8 (serine proteinase)
TGGT1_202020-t26_1	484.5922853	64.66031609	2.9058	8.43E-05	0.003768	tetratricopeptide repeat-containing protein DnaK
TGGT1_274170-t26_1	42.76525246	5.719349487	2.9025	0.000181	0.006736	Predicted rhoptry-kinase-like (ROPKL) kinase
TGGT1_280580-t26_1	367.4347461	49.1882385	2.9011	0.002288	0.045303	SAG4.2
TGGT1_219218-t26_1	120.5214594	16.63256482	2.8572	1.83E-10	3.88E-08	hypothetical protein
TGGT1_220025-t26_1	58.92272106	8.224296257	2.8409	1.27E-05	0.000832	hypothetical protein
TGGT1_223262-t26_1	123.9077142	17.49524264	2.8242	6.30E-10	1.23E-07	WD domain, G-beta repeat-containing protein
TGGT1_207160-t26_1	186.1273516	26.57358238	2.8082	2.71E-08	3.92E-06	SAG2Y
TGGT1_248425-t26_1	66.31956568	9.608140554	2.7871	3.76E-06	0.00031	hypothetical protein
TGGT1_240200-t26_1	77.11640943	11.54821892	2.7394	0.000353	0.011529	hypothetical protein
TGGT1_268860-t26_1	106.3967745	16.03694599	2.73	1.56E-08	2.47E-06	enolase
TGGT1_262970-t26_1	2103.927612	317.3410749	2.729	2.43E-56	4.08E-53	hypothetical protein
TGGT1_305280-t26_1	48.48958626	7.326550844	2.7265	0.000127	0.005236	hypothetical protein
TGGT1_253760-t26_1	89.89105937	13.79474104	2.7041	1.82E-07	2.18E-05	hypothetical protein
TGGT1_222340-t26_1	30.98036464	4.896056428	2.6617	0.002423	0.0469	NOL1/NOP2/sun family protein
TGGT1_313050-t26_1	59.60718781	9.419851086	2.6617	0.000747	0.020865	hypothetical protein
TGGT1_312435-t26_1	41.07040703	6.542642546	2.6502	0.000437	0.013835	hypothetical protein
TGGT1_243470-t26_1	50.50690753	8.224296257	2.6185	0.000144	0.005668	hypothetical protein
TGGT1_232420-t26_1	53.19323311	8.710395141	2.6104	7.53E-05	0.003488	hypothetical protein
TGGT1_245770B-t26_1	48.82924257	8.224296257	2.5698	0.000399	0.012851	hypothetical protein
TGGT1_204290-t26_1	33.66669023	5.719349487	2.5574	0.001842	0.039787	hypothetical protein
TGGT1_224170-t26_1	92.24975498	15.7348194	2.5516	2.05E-07	2.33E-05	SAG-related sequence
TGGT1_291040-t26_1	47.13611519	8.149843903	2.532	0.00031	0.010384	lactate dehydrogenase LDH2
TGGT1_238915-t26_1	28.2699864	4.896056428	2.5296	0.002611	0.049514	hypothetical protein
TGGT1_207150-t26_1	217.1180245	38.45899548	2.4971	2.02E-14	7.06E-12	SAG2Y
TGGT1_316635-t26_1	125.9301896	22.76356083	2.4678	1.13E-08	1.83E-06	DNA-directed RNA polymerase II subunit rpb1
TGGT1_203800-t26_1	33.99260215	6.20544837	2.4536	0.001573	0.035339	hypothetical protein
TGGT1_290970-t26_1	2576.472666	477.1197634	2.433	8.00E-38	6.72E-35	8-amino-7-oxononanoate synthase
TGGT1_245980-t26_1	391.7245504	72.78372673	2.4282	0.000492	0.015239	hypothetical protein
TGGT1_365480-t26_1	171.9683057	32.40676898	2.4078	0.000583	0.017444	hypothetical protein
TGGT1_293780-t26_1	1252.091801	238.5666226	2.3919	1.80E-15	7.97E-13	hypothetical protein
TGGT1_290645-t26_1	39.05823991	7.475455552	2.3854	0.001449	0.033734	hypothetical protein
TGGT1_245770A-t26_1	258.9192855	50.64653515	2.354	3.34E-12	9.68E-10	hypothetical protein
TGGT1_250000-t26_1	75.40266549	14.84139116	2.345	9.65E-06	0.000676	hypothetical protein
TGGT1_232170-t26_1	37.36511254	7.401003198	2.3359	0.001543	0.035027	hypothetical protein
TGGT1_227630-t26_1	871.105164	173.0430984	2.3317	2.94E-12	8.81E-10	hypothetical protein
TGGT1_238440-t26_1	79.11826827	15.73913657	2.3297	1.16E-05	0.000774	SAG-related sequence
TGGT1_286460-t26_1	161.9143423	32.37601855	2.3222	6.10E-10	1.22E-07	hypothetical protein
TGGT1_285445-t26_1	60.92286185	12.18753968	2.3216	6.53E-05	0.003099	hypothetical protein
TGGT1_236880-t26_1	58.555576	11.77589315	2.314	0.002026	0.042134	hypothetical protein
TGGT1_462970-t26_1	36.35301581	7.326550844	2.3109	0.001876	0.039909	hypothetical protein
TGGT1_304930-t26_1	202.7086608	41.00764417	2.3054	1.64E-06	0.000145	hypothetical protein
TGGT1_265320-t26_1	114.1229671	23.5517863	2.2767	2.03E-07	2.33E-05	hypothetical protein
TGGT1_202430-t26_1	329.5976876	68.1067102	2.2748	4.50E-16	2.10E-13	hypothetical protein
TGGT1_265255-t26_1	58.24856259	12.22260727	2.2527	0.000183	0.006756	hypothetical protein

TGGT1_295720-t26_1	95.63257364	20.37245118	2.2309	3.66E-05	0.001957	putative sulfite oxidase
TGGT1_316560-t26_1	93.25669756	20.07464176	2.2158	4.01E-06	0.000324	D protein family
TGGT1_215955-t26_1	45.78264412	9.905949969	2.2084	0.000877	0.023436	hypothetical protein
TGGT1_219450-t26_1	106.3658497	23.02630265	2.2077	3.88E-07	4.12E-05	hypothetical protein
TGGT1_212275-t26_1	147.4259484	32.03882438	2.2021	5.85E-08	7.80E-06	hypothetical protein
TGGT1_411080-t26_1	317.7200253	69.22781267	2.1983	3.09E-06	0.00026	hypothetical protein
TGGT1_213700-t26_1	89.89449546	19.62361047	2.1956	5.51E-06	0.000421	hypothetical protein
TGGT1_295960-t26_1	1315.41631	289.532553	2.1837	1.13E-19	7.27E-17	hypothetical protein
TGGT1_259020-t26_1	113.790183	25.1196029	2.1795	4.18E-07	4.39E-05	BAG1
TGGT1_264150-t26_1	106.0347836	24.03788518	2.1412	1.21E-06	0.000113	hypothetical protein
TGGT1_268985-t26_1	74.39056877	16.96975899	2.1322	2.62E-05	0.001493	hypothetical protein
TGGT1_256800-t26_1	43.09975462	9.905949969	2.1213	0.002571	0.048973	hypothetical protein
TGGT1_305610-t26_1	244.1066978	56.18622951	2.1192	2.41E-11	6.32E-09	hypothetical protein
TGGT1_209755A-t26_1	93.58776363	21.63814119	2.1127	1.93E-05	0.001191	hypothetical protein
TGGT1_209755B-t26_1	156.5434092	37.937829	2.0449	1.68E-08	2.60E-06	hypothetical protein
TGGT1_295020-t26_1	120.8679879	29.41572333	2.0388	7.07E-07	6.91E-05	Sterol-sensing domain of SREBP cleavage-activation domain-containing protein
TGGT1_207470-t26_1	111.779734	27.28303832	2.0346	5.24E-06	0.000404	hypothetical protein
TGGT1_293560-t26_1	68.33688694	16.70701717	2.0322	9.93E-05	0.004302	hypothetical protein
TGGT1_269680-t26_1	53.18464288	13.01083274	2.0313	0.000887	0.023575	putative acyl-CoA carboxyltransferase beta chain
TGGT1_208380-t26_1	160.2349593	39.36537522	2.0252	2.62E-08	3.87E-06	hypothetical protein
TGGT1_270670-t26_1	324.5698469	80.10596041	2.0185	3.62E-13	1.17E-10	hypothetical protein
TGGT1_295950-t26_1	2018.726973	500.1369015	2.0131	9.98E-25	6.99E-22	KRUF family protein
TGGT1_207350-t26_1	49.47934838	12.44596433	1.9911	0.000818	0.022184	hypothetical protein
TGGT1_293480-t26_1	2781.526278	701.3300332	1.9877	7.79E-35	5.95E-32	MoeA N-terminal region (domain I and II) domain-containing protein
TGGT1_364790-t26_1	43.0842922	10.87814774	1.9857	0.002434	0.04702	hypothetical protein
TGGT1_268190-t26_1	48.47927798	12.26199203	1.9832	0.002054	0.042502	hypothetical protein
TGGT1_269075-t26_1	104.7191095	26.91077655	1.9603	8.33E-05	0.003743	hypothetical protein
TGGT1_301000-t26_1	75.76981055	19.47470576	1.96	0.002646	0.049964	Carnosine-methyltransferase
TGGT1_267130-t26_1	158.5521402	41.15654888	1.9458	5.44E-08	7.50E-06	SAG-related sequence
TGGT1_225105-t26_1	354.4212876	92.43808763	1.9389	4.97E-07	5.03E-05	hypothetical protein
TGGT1_270260-t26_1	253.4847847	66.20601659	1.9369	8.40E-11	2.14E-08	hypothetical protein
TGGT1_219110-t26_1	98.94151629	25.8684436	1.9354	0.002046	0.042439	hypothetical protein
TGGT1_258910-t26_1	53.5139909	14.20638757	1.9134	0.001015	0.02613	hypothetical protein
TGGT1_321490-t26_1	215.7353466	57.6051414	1.905	8.77E-05	0.00387	SAG-related sequence
TGGT1_252395-t26_1	81.13215344	21.71691071	1.9015	7.75E-05	0.003539	hypothetical protein
TGGT1_205050-t26_1	91.88432797	25.1196029	1.871	2.05E-05	0.001232	hypothetical protein
TGGT1_267875-t26_1	77.76823329	21.37971654	1.8629	0.000136	0.005436	Dynein regulatory complex protein 1
TGGT1_321480-t26_1	164.6607995	45.26869701	1.8629	8.78E-05	0.00387	SAG-related sequence
TGGT1_266035-t26_1	76.41476222	21.08622429	1.8576	0.000243	0.008547	hypothetical protein
TGGT1_309030-t26_1	76.74926438	21.19574423	1.8564	0.000162	0.006228	hypothetical protein
TGGT1_208730-t26_1	6062.769763	1683.378082	1.8486	1.62E-14	5.93E-12	putative microneme protein
TGGT1_268220-t26_1	175.4146921	48.96919861	1.8408	1.39E-07	1.74E-05	hypothetical protein
TGGT1_288520-t26_1	104.3760172	29.30620338	1.8325	3.07E-05	0.001663	ATPase, AAA family protein
TGGT1_244590-t26_1	54.54498614	15.47639475	1.8174	0.002624	0.049646	katanin-like family protein
TGGT1_200340-t26_1	114.8039978	32.7045784	1.8116	8.19E-06	0.000593	hypothetical protein
TGGT1_233210-t26_1	74.72850702	21.34464894	1.8078	0.000236	0.008491	hypothetical protein
TGGT1_409260-t26_1	366.0159892	105.1861785	1.799	2.15E-05	0.001283	hypothetical protein
TGGT1_292935-t26_1	160.5986682	47.58967148	1.7547	1.44E-06	0.000132	hypothetical protein
TGGT1_225070-t26_1	51.16044943	15.21365293	1.7497	0.002248	0.045146	hypothetical protein
TGGT1_220420-t26_1	91.24109435	27.13845078	1.7493	0.000147	0.005738	3'5'-cyclic nucleotide phosphodiesterase domain-containing protein
TGGT1_203720-t26_1	456.5107671	137.8600065	1.7274	2.73E-13	9.18E-11	vitamin k epoxide reductase family protein
TGGT1_297350-t26_1	103.6829602	31.51334073	1.7181	5.07E-05	0.00255	Exosome complex component Rrp41
TGGT1_220460-t26_1	73.70610202	22.57958853	1.7068	0.000818	0.022184	SNF7 family protein
TGGT1_228175-t26_1	114.1384296	35.10000522	1.7012	4.08E-05	0.002114	hypothetical protein
TGGT1_201840-t26_1	1276.135241	393.8652183	1.696	0.000518	0.015708	aspartyl protease ASP1
TGGT1_235650-t26_1	56.89165542	17.8675044	1.6709	0.002528	0.048492	carrier superfamily protein
TGGT1_259270-t26_1	102.3535417	32.18772908	1.669	0.000164	0.006251	SAG-related sequence
TGGT1_310160-t26_1	122.8715648	38.80050682	1.663	1.63E-05	0.00103	argonaute AGO
TGGT1_221220-t26_1	104.0346428	32.85780027	1.6628	0.000109	0.004585	hypothetical protein
TGGT1_216710-t26_1	93.59119973	29.75723467	1.6531	0.000242	0.008542	transporter, major facilitator family protein
TGGT1_285940-t26_1	68.32142452	22.09348965	1.6287	0.000967	0.025152	hypothetical protein
TGGT1_266670-t26_1	61.59358422	20.00018941	1.6228	0.001849	0.039787	hypothetical protein
TGGT1_281400-t26_1	766.2577179	248.9759401	1.6218	5.93E-13	1.85E-10	phosphofructokinase domain-containing protein
TGGT1_293258-t26_1	112.7901126	36.82104369	1.615	0.000101	0.004364	hypothetical protein
TGGT1_293290-t26_1	202.6141682	66.28046894	1.6121	0.000263	0.009167	hypothetical protein
TGGT1_218500-t26_1	105.0055064	34.35116451	1.612	0.001124	0.028019	hypothetical protein
TGGT1_257670-t26_1	106.7312767	35.69562405	1.5802	0.000144	0.005668	hypothetical protein
TGGT1_264485-t26_1	384.7618547	130.9715355	1.5547	3.55E-09	6.48E-07	AP2 domain transcription factor AP2IX-3
TGGT1_213020-t26_1	734.8414864	250.2281484	1.5542	3.84E-15	1.61E-12	hypothetical protein
TGGT1_312930-t26_1	170.0334507	58.05617268	1.5503	1.02E-05	0.00069	cystathionine beta-lyase
TGGT1_233920-t26_1	421.1354871	144.0740892	1.5475	9.27E-11	2.23E-08	zinc finger, C3HC4 type (RING finger) domain-containing protein
TGGT1_223480-t26_1	223.197477	76.55868068	1.5437	3.05E-07	3.35E-05	sushi domain (scr repeat) domain-containing protein
TGGT1_227370-t26_1	115.8143765	39.73763699	1.5432	0.000137	0.005436	hydrolase CocE/NonD family protein
TGGT1_210400-t26_1	236.3135013	81.27076481	1.5399	1.43E-07	1.77E-05	hypothetical protein
TGGT1_288640-t26_1	2346.647679	812.0467415	1.531	9.22E-06	0.00066	radical SAM domain-containing protein
TGGT1_269050-t26_1	471.7145526	163.7548834	1.5264	3.01E-06	0.000255	putative adenylate kinase
TGGT1_208740-t26_1	6201.903431	2153.627173	1.5259	4.43E-08	6.20E-06	putative microneme protein
TGGT1_253460-t26_1	154.529524	53.8695722	1.5203	2.04E-05	0.001232	hypothetical protein
TGGT1_215180-t26_1	60.91598966	21.27019659	1.518	0.002581	0.049067	hypothetical protein
TGGT1_228160-t26_1	145.0620987	51.06681602	1.5062	0.000892	0.023651	acid phosphatase
TGGT1_254430-t26_1	89.23236332	32.14834432	1.4728	0.001696	0.037903	putative microneme protein
TGGT1_293280-t26_1	2513.65343	908.2905335	1.4686	6.99E-07	6.91E-05	cyclin protein-U4
TGGT1_223725-t26_1	323.5800848	117.1066592	1.4663	0.000167	0.006302	hypothetical protein

TGGT1_213255-t26_1	467.9422543	172.0579491	1.4434	1.45E-10	3.29E-08	hypothetical protein
TGGT1_307590-t26_1	99.6465996	36.71152374	1.4406	0.000718	0.020477	hypothetical protein
TGGT1_281460-t26_1	124.887168	46.23657761	1.4335	8.33E-05	0.003743	hypothetical protein
TGGT1_310480-t26_1	228.3026298	84.55098554	1.4331	0.002131	0.043274	PACRG family protein
TGGT1_215970-t26_1	798.4317753	298.6454301	1.4187	1.69E-10	3.73E-08	hypothetical protein
TGGT1_220585-t26_1	88.52556197	33.19499444	1.4151	0.000936	0.024492	hypothetical protein
TGGT1_287960-t26_1	221.2110806	83.43420024	1.4067	4.55E-06	0.00036	hypothetical protein
TGGT1_203290-t26_1	2497.79662	952.9555076	1.3902	2.31E-19	1.29E-16	hypothetical protein
TGGT1_249840-t26_1	340.6998267	131.3259983	1.3753	7.96E-08	1.03E-05	putative dynein heavy chain 1
TGGT1_294400-t26_1	392.4330699	154.2648971	1.347	0.001489	0.03428	Rhoptry neck protein 2
TGGT1_253330-t26_1	697.3870354	275.628292	1.3392	0.000712	0.020415	Predicted rhoptry-kinase-like (ROPKL) pseudokinase
TGGT1_205658-t26_1	82.12878774	32.78334792	1.3249	0.002253	0.045146	F5/8 type C domain-containing protein
TGGT1_294805-t26_1	87.85483959	35.21384233	1.319	0.001992	0.041537	hypothetical protein
TGGT1_206550-t26_1	391.1792455	156.8529306	1.3184	3.00E-08	4.27E-06	hypothetical protein
TGGT1_319520-t26_1	310.0934793	125.3352727	1.3069	7.88E-07	7.61E-05	hypothetical protein
TGGT1_248240-t26_1	169.6697417	68.70664619	1.3042	6.26E-05	0.003025	hypothetical protein
TGGT1_281930-t26_1	205.9987049	84.29256089	1.2892	7.90E-06	0.000577	SAG-related sequence
TGGT1_312330-t26_1	2124.730831	874.7809912	1.2803	1.85E-10	3.88E-08	DNA-directed RNA polymerase II subunit RPB1
TGGT1_272730-t26_1	183.4685147	77.30752139	1.2469	7.66E-05	0.003515	hypothetical protein
TGGT1_203890-t26_1	92.56192253	39.17708575	1.2404	0.002359	0.046093	hypothetical protein
TGGT1_308965-t26_1	166.2869231	70.72981125	1.2333	0.000136	0.005436	hypothetical protein
TGGT1_272370-t26_1	6010.120117	2569.366916	1.226	3.94E-09	7.04E-07	hypothetical protein
TGGT1_275870-t26_1	102.3380793	43.81040036	1.224	0.002132	0.043274	tubulin/FtsZ family, GTPase domain-containing protein
TGGT1_220720-t26_1	269.6731781	116.213231	1.2144	7.16E-06	0.000532	hypothetical protein
TGGT1_300130-t26_1	282.1322244	121.9805996	1.2097	9.81E-06	0.000681	apical membrane antigen 1 domain-containing protein
TGGT1_209040-t26_1	549.9524968	245.5640833	1.1632	0.002366	0.046122	hypothetical protein
TGGT1_215340-t26_1	112.7609058	50.69023708	1.1535	0.001819	0.039599	AP2 domain transcription factor AP2X-10
TGGT1_236670A-t26_1	281.4666562	126.7585017	1.1509	2.04E-05	0.001232	hypothetical protein
TGGT1_205265-t26_1	532.5343316	242.3847482	1.1356	5.58E-08	7.56E-06	transporter, cation channel family protein
TGGT1_293500-t26_1	249.774336	114.1943831	1.1291	2.00E-05	0.001226	hypothetical protein
TGGT1_219650-t26_1	498.6654288	230.8235778	1.1113	0.000282	0.009635	transporter, small conductance mechanosensitive ion channel (MscS) protein
TGGT1_294785-t26_1	151.5104143	70.12987525	1.1113	0.001598	0.035813	zinc finger (CCCH type) motif-containing protein
TGGT1_306620-t26_1	1229.966036	569.7321285	1.1103	8.38E-09	1.38E-06	AP2 domain transcription factor AP2IX-9
TGGT1_291170-t26_1	175.4026658	82.6896767	1.0849	0.00075	0.020865	hypothetical protein
TGGT1_212160-t26_1	205.0037886	98.35004375	1.0597	0.000226	0.008213	hypothetical protein
TGGT1_286740-t26_1	183.4960035	88.48347854	1.0523	0.001096	0.027746	microneme-like protein
TGGT1_255290-t26_1	221.8388518	106.9859865	1.0521	0.000176	0.006587	hypothetical protein
TGGT1_245440-t26_1	277.7647978	134.1244373	1.0503	0.000107	0.004557	hypothetical protein
TGGT1_267460-t26_1	142.7205835	69.15336032	1.0453	0.00127	0.030479	AP2 domain transcription factor AP2IX-1
TGGT1_297910-t26_1	127.2321192	62.27352359	1.0308	0.001968	0.041335	hypothetical protein
TGGT1_315123-t26_1	247.4396931	121.6083378	1.0248	0.000239	0.008517	hypothetical protein
TGGT1_254640-t26_1	183.1305765	90.05129513	1.0241	0.000719	0.020477	hypothetical protein
TGGT1_233490-t26_1	3188.210651	1589.295074	1.0044	2.62E-08	3.87E-06	hypothetical protein
TGGT1_250100-t26_1	2119.712782	1058.373147	1.002	8.93E-11	2.21E-08	hypothetical protein
TGGT1_363340-t26_1	306.0141676	153.108727	0.99904	5.82E-05	0.002861	hypothetical protein
TGGT1_250220-t26_1	3793.505992	1903.646694	0.99477	6.93E-12	1.94E-09	hypothetical protein
TGGT1_215500-t26_1	258.5263698	130.6866775	0.9842	0.000166	0.006292	TPR repeat-containing protein
TGGT1_222160-t26_1	845.7929546	430.0113434	0.97593	0.001283	0.030704	aldehyde dehydrogenase
TGGT1_200350-t26_1	1347.962666	685.731167	0.97507	5.98E-09	1.00E-06	subtilisin SUB3
TGGT1_272440-t26_1	200.3207583	102.5673947	0.96574	0.001017	0.02613	RNA recognition motif-containing protein
TGGT1_223750-t26_1	460.231524	235.8728561	0.96435	1.56E-05	0.001	hypothetical protein
TGGT1_212810-t26_1	181.0995108	94.16344326	0.94354	0.00138	0.032386	hypothetical protein
TGGT1_245428-t26_1	942.253794	492.0172777	0.93741	1.94E-07	2.26E-05	hypothetical protein
TGGT1_232590-t26_1	1107.302355	583.846795	0.92339	1.92E-07	2.26E-05	glutamate-cysteine ligase, catalytic subunit domain-containing protein
TGGT1_304730-t26_1	402.9194641	214.9177376	0.90671	0.001469	0.034002	mob1 phocein family protein
TGGT1_200320-t26_1	500.27609	267.2416093	0.90458	2.75E-05	0.001528	hypoxanthine-xanthine-guanine phosphoribosyl transferase
TGGT1_239810-t26_1	723.7805804	388.201992	0.89874	2.27E-06	0.000195	hypothetical protein
TGGT1_218740-t26_1	488.8291404	263.2303468	0.893	2.78E-05	0.001539	hypothetical protein
TGGT1_235460-t26_1	200.963992	108.5581203	0.88847	0.001867	0.039854	hypothetical protein
TGGT1_266870-t26_1	630.5479355	341.1815061	0.88607	9.26E-06	0.00066	transporter, major facilitator family protein
TGGT1_224710-t26_1	210.7057878	114.5402116	0.87938	0.001903	0.040173	hypothetical protein
TGGT1_215910-t26_1	7953.027456	4337.198355	0.87474	2.71E-10	5.55E-08	hypothetical protein
TGGT1_257360-t26_1	369.9372406	202.2354647	0.87124	0.000106	0.004532	hypothetical protein
TGGT1_273350-t26_1	398.6585564	218.3031611	0.86882	0.001295	0.03083	molybdopterin converting factor, subunit 2 protein
TGGT1_239090-t26_1	597.527915	330.3427431	0.85504	1.76E-05	0.001103	SAG-related sequence
TGGT1_254960-t26_1	290.8378709	160.8248083	0.85473	0.002559	0.04887	hypothetical protein
TGGT1_214220-t26_1	14512.67401	8060.70308	0.84834	1.64E-07	2.00E-05	hypothetical protein
TGGT1_269417-t26_1	813.2443631	453.6505335	0.84211	4.25E-06	0.00034	hypothetical protein
TGGT1_248140-t26_1	240.3687604	136.0645157	0.82096	0.002114	0.043217	hypothetical protein
TGGT1_244710-t26_1	671.2202726	382.9115577	0.80978	5.21E-05	0.002604	hypothetical protein
TGGT1_214575-t26_1	541.9862945	309.81707	0.80684	7.17E-05	0.003383	hypothetical protein
TGGT1_266910-t26_1	740.5812826	424.274195	0.80366	1.29E-05	0.00084	putative cell-cycle-associated protein kinase GSK
TGGT1_289050-t26_1	5560.864114	3191.086253	0.80126	7.64E-08	1.00E-05	putative FIKK kinase
TGGT1_233925-t26_1	403.9934105	234.8040899	0.78287	0.000795	0.0219	hypothetical protein
TGGT1_234180-t26_1	732.8310373	427.8694938	0.77631	2.63E-05	0.001493	hypothetical protein
TGGT1_225790-t26_1	669.5958671	395.06456	0.7612	5.96E-05	0.002912	Protein disulfide isomerase (PDI) family protein
TGGT1_225540-t26_1	2505.026814	1486.776759	0.75264	3.18E-05	0.001712	hypothetical protein
TGGT1_237510-t26_1	756.6631571	449.2055084	0.75228	0.000876	0.023436	hypothetical protein
TGGT1_236620-t26_1	820.6704145	505.2557847	0.69979	8.17E-05	0.003713	protein kinase
TGGT1_239020-t26_1	1638.979214	1020.811897	0.68308	6.32E-05	0.003034	ABC transporter transmembrane region domain-containing protein
TGGT1_220230A-t26_1	853.4246547	536.0947371	0.67078	0.000304	0.010229	hypothetical protein
TGGT1_236890-t26_1	2101.693817	1321.533359	0.66934	0.002082	0.042938	hypothetical protein
TGGT1_266880-t26_1	1139.956949	718.9126797	0.66509	0.000154	0.005959	putative tRNA-dihydrouridine synthase

TGGT1_208450-t26_1	4005.96624	2535.22237	0.66004	7.56E-05	0.003488	kazal-type serine protease inhibitor domain-containing protein
TGGT1_236010-t26_1	2955.645395	1884.530238	0.64927	1.02E-05	0.00069	prenylcysteine oxidase
TGGT1_283810-t26_1	1166.101377	749.9005368	0.63692	0.000125	0.005191	putative raffinose synthase 1
TGGT1_293810-t26_1	636.9464278	413.1419397	0.62453	0.001469	0.034002	carboxyvinyl-carboxyphosphonate phosphorylmutase
TGGT1_230170-t26_1	4119.849197	2693.786645	0.61296	0.00013	0.005291	hypothetical protein
TGGT1_273320-t26_1	2009.605559	1316.685321	0.61	4.65E-05	0.002392	hypothetical protein
TGGT1_213445-t26_1	3766.730356	2478.891553	0.60362	3.81E-05	0.002012	hypothetical protein
TGGT1_278940-t26_1	1446.650429	952.7925909	0.60248	0.000416	0.013241	HECT-domain (ubiquitin-transferase) domain-containing protein
TGGT1_263220-t26_1	614.359542	404.996413	0.60117	0.001896	0.040117	rhoptry kinase family protein ROP21
TGGT1_263270-t26_1	1324.888372	873.4138853	0.60113	0.000223	0.00815	glycerophosphodiester phosphodiesterase family protein
TGGT1_219230-t26_1	1144.926376	761.1158787	0.58907	0.000475	0.014833	AMP-binding enzyme domain-containing protein
TGGT1_203060-t26_1	3012.364045	2006.83455	0.58597	0.000117	0.004884	TrmH family RNA methyltransferase
TGGT1_262630-t26_1	604.2385748	402.8944784	0.58472	0.00185	0.039787	hypothetical protein
TGGT1_311100-t26_1	5409.314184	3610.907318	0.58308	0.000237	0.008491	zinc finger (CCCH type) motif-containing protein
TGGT1_269425-t26_1	1103.732269	738.9436195	0.57885	0.00059	0.017579	hypothetical protein
TGGT1_203870-t26_1	797.2020036	534.5619881	0.57659	0.00215	0.043537	RAB43, member RAS oncogene family protein
TGGT1_269430-t26_1	1115.635702	751.2315145	0.57054	0.000916	0.024052	polyprenyl synthetase superfamily protein
TGGT1_316430-t26_1	1144.015644	772.8308012	0.56588	0.00112	0.027997	putative target of rapamycin (TOR)
TGGT1_213280-t26_1	3688.333151	2507.024318	0.55699	0.000332	0.01103	SAG-related sequence
TGGT1_204540-t26_1	1423.399693	968.5101416	0.5555	0.000901	0.023785	DUF367 domain-containing protein
TGGT1_263740-t26_1	1367.380972	943.7978681	0.53487	0.00073	0.020588	ABC transporter transmembrane region domain-containing protein
TGGT1_208570-t26_1	3363.038805	2328.664352	0.53026	0.00029	0.00987	putative ubiquitin conjugating enzyme E2
TGGT1_203790-t26_1	3172.121904	2199.050758	0.52857	0.000274	0.00943	hypothetical protein
TGGT1_237550-t26_1	1539.153938	1068.256981	0.52688	0.001229	0.030098	hypothetical protein
TGGT1_205680-t26_1	993.6662793	690.2107295	0.52572	0.001234	0.030112	hypothetical protein
TGGT1_304670-t26_1	1343.411409	933.9836391	0.52443	0.001339	0.03152	leucine rich repeat-containing protein
TGGT1_216770-t26_1	2549.801314	1803.014	0.49997	0.001108	0.027964	hypothetical protein
TGGT1_300055-t26_1	1707.763477	1222.964275	0.48173	0.001976	0.041404	hypothetical protein
TGGT1_204050-t26_1	12721.38737	9124.079152	0.47951	0.000413	0.013204	subtilisin SUB1
TGGT1_208370-t26_1	5762.545732	4169.813635	0.46672	0.000798	0.021914	putative myosin heavy chain
TGGT1_286090-t26_1	9065.382738	6574.291614	0.46353	0.001119	0.027997	putative translation initiation factor SUI1
TGGT1_268980-t26_1	2197.846442	1606.169325	0.45247	0.002308	0.045418	hypothetical protein
TGGT1_239420-t26_1	11916.54748	8847.030888	0.4297	0.002281	0.045297	calcium-dependent protein kinase

S4 Table. ToxoDB
KOPiTvsPar.DEG_up sort
