# Supplementary material for: A single Na+-Pi cotransporter in Toxoplasma plays key roles in phosphate import and control of parasite osmoregulation
Source: PLoS Pathog. 2020 Dec 31;16(12):e1009067. doi: 10.1371/journal.ppat.1009067 (PMC7817038; doi:10.1371/journal.ppat.1009067)
Supplement: S5 Table — (PDF) [file ppat.1009067.s011.pdf]

Gene_id	readcount_DeltaTgPiT	readcount_Parental	logFC	pval	padj	Annotation ToxoDB
TGGT1_226755-t26_1	96.27237117	1315.37593	-3.7722	3.16E-70	6.63E-67	3'5'-cyclic nucleotide phosphodiesterase domain-containing protein
TGGT1_226450-t26_1	16.49884295	59.51015217	-1.8508	0.00074724	0.020865	hypothetical protein
TGGT1_256650-t26_1	73.38706228	210.525579	-1.5204	1.14E-06	0.00010806	hypothetical protein
TGGT1_220330-t26_1	133.3081357	375.8655476	-1.4955	2.24E-09	4.19E-07	hypothetical protein
TGGT1_239350-t26_1	529.5805077	1471.387768	-1.4743	5.57E-18	2.92E-15	RAP domain-containing protein
TGGT1_254890-t26_1	69.02135369	181.6660897	-1.3962	1.05E-05	0.00070616	hypothetical protein
TGGT1_411400-t26_1	99.9604852	238.9469884	-1.2573	2.92E-05	0.0016033	hypothetical protein
TGGT1_329800-t26_1	242.108275	548.4710965	-1.1798	0.00025114	0.0087918	hypothetical protein
TGGT1_308090-t26_1	12770.00014	27390.95681	-1.1009	1.51E-14	5.93E-12	ROP5
TGGT1_261022-t26_1	2909.590616	6027.365613	-1.0507	5.06E-06	0.00039697	cytoplasmic dynein heavy chain family protein
TGGT1_261075-t26_1	227.8513005	464.410527	-1.0273	0.00090305	0.023785	hypothetical protein
TGGT1_276910-t26_1	180.1320833	357.770369	-0.98998	1.60E-05	0.0010196	ER lumen protein retaining receptor (ERD2) family protein
TGGT1_310740-t26_1	434.8174329	855.9186427	-0.97706	0.00033893	0.011167	hypothetical protein
TGGT1_255245-t26_1	211.7866064	415.8222245	-0.97336	6.74E-06	0.00050551	hypothetical protein
TGGT1_269930-t26_1	482.025706	940.4259263	-0.9642	4.56E-07	4.73E-05	calcium binding EGF domain-containing protein
TGGT1_238040B-t26_1	93.92913798	180.5449872	-0.94271	0.0020893	0.042938	protein disulfide-isomerase domain-containing
TGGT1_267140-t26_1	221.5232481	425.6537221	-0.94222	2.43E-05	0.0014094	SAG-related sequence
TGGT1_275650-t26_1	1767.331667	3390.169876	-0.93978	3.09E-07	3.35E-05	hypothetical protein
TGGT1_273970-t26_1	184.7807527	340.4369825	-0.88157	0.00065581	0.019237	CorA family Mg2+ transporter protein
TGGT1_225990-t26_1	615.1058585	1128.704794	-0.87576	3.11E-07	3.35E-05	putative malonyl CoA-acyl carrier protein transacylase
TGGT1_313520-t26_1	132.6305411	241.2550114	-0.86315	0.0020902	0.042938	hypothetical protein
TGGT1_411390-t26_1	227.1994766	411.1187747	-0.8556	0.00038582	0.012516	hypothetical protein
TGGT1_209090-t26_1	596.8262678	1077.125446	-0.8518	3.51E-06	0.00029213	proteasome maturation factor ump1 protein
TGGT1_262590-t26_1	297.2758782	536.3445274	-0.85136	3.05E-05	0.0016629	Nucleolar protein 58
TGGT1_262040-t26_1	662.1990225	1190.58447	-0.84633	0.00033831	0.011167	SAC3/GANP family protein
TGGT1_217182-t26_1	1956.384837	3513.354665	-0.84466	1.09E-07	1.38E-05	RNA-dependent RNA polymerase RDP
TGGT1_293840-t26_1	3718.789511	6638.524076	-0.83603	1.70E-08	2.60E-06	hypothetical protein
TGGT1_217700-t26_1	767.7326534	1366.876508	-0.83221	2.72E-05	0.0015282	AP2 domain transcription factor AP2XII-2
TGGT1_219240-t26_1	220.8164468	392.53318	-0.82997	0.00057851	0.017359	Peptidyl-tRNA hydrolase PTH2 domain-containing protein
TGGT1_244180-t26_1	159.2297348	282.2712899	-0.82597	0.0012472	0.030112	microneme-like protein
TGGT1_295080-t26_1	195.8983542	344.7201515	-0.81532	0.0015627	0.03523	hypothetical protein
TGGT1_261070-t26_1	949.8769038	1663.096291	-0.80806	1.34E-05	0.00086921	apicoplast triosephosphate translocator APT1
TGGT1_320110-t26_1	691.8035814	1193.500533	-0.78676	5.88E-06	0.00044526	proliferating cell nuclear antigen PCNA2
TGGT1_309265-t26_1	547.0330338	940.2198379	-0.78137	1.86E-05	0.0011594	oxidoreductase, short chain dehydrogenase/reductase family protein
TGGT1_203930-t26_1	1690.077297	2896.325296	-0.77713	1.77E-06	0.00015474	hypothetical protein
TGGT1_201670-t26_1	361.2504927	618.9991365	-0.77694	5.24E-05	0.0026042	DnaJ domain-containing protein
TGGT1_255310-t26_1	723.9059978	1235.76955	-0.77154	1.63E-06	0.00014525	zinc finger (CCCH type) motif-containing protein
TGGT1_272290-t26_1	880.960351	1502.017375	-0.76975	7.33E-06	0.00054011	pyruvate dehydrogenase complex subunit PD-HE1Beta
TGGT1_309160-t26_1	464.83037	791.5297028	-0.76794	0.0017841	0.039242	IgA-specific metalloendopeptidase
TGGT1_251500-t26_1	1959.893073	3336.181089	-0.76742	6.01E-07	6.02E-05	putative eukaryotic initiation factor-3
TGGT1_224800-t26_1	204.6830308	348.1228436	-0.7662	0.0013088	0.031065	ER lumen protein retaining receptor
TGGT1_409990-t26_1	1009.180514	1705.81634	-0.75728	9.65E-06	0.00067587	hypothetical protein
TGGT1_202030-t26_1	744.0792104	1253.895479	-0.75289	3.90E-06	0.00031851	poly(ADP-ribose) polymerase catalytic domain protein
TGGT1_266320-t26_1	821.9431373	1371.246551	-0.73838	0.0018051	0.039396	glycosyltransferase family protein
TGGT1_232390-t26_1	216.4558923	358.3659878	-0.72736	0.0024488	0.047189	18S rRNA biogenesis protein RCL1 protein
TGGT1_245670-t26_1	1086.336439	1793.879537	-0.72361	1.18E-05	0.00077892	pyruvate dehydrogenase complex subunit PDH-E1Alpha
TGGT1_253880-t26_1	998.0783752	1644.335359	-0.72028	2.54E-05	0.0014636	GNS1/SUR4 family protein
TGGT1_254900-t26_1	757.0526368	1242.12822	-0.71435	6.47E-05	0.0030905	proteasome subunit beta type 2
TGGT1_263470-t26_1	351.4399749	576.5116098	-0.71407	0.00074958	0.020865	ubiquitin carboxyl-terminal hydrolase UCHL3
TGGT1_239010-t26_1	947.9008157	1547.67992	-0.7073	4.70E-05	0.0023957	hypothetical protein
TGGT1_315620-t26_1	374.6683762	610.6043497	-0.70462	0.00067868	0.019663	putative vacuolar ATP synthase subunit C
TGGT1_210420-t26_1	616.6254631	1001.810339	-0.70014	0.0003217	0.010726	hypothetical protein
TGGT1_239530-t26_1	920.2075761	1491.389018	-0.69663	0.0015348	0.035027	alanine-glyoxylate aminotransferase
TGGT1_247460-t26_1	1953.714491	3160.273733	-0.69383	2.01E-06	0.00017441	proliferating cell nuclear antigen PCNA1
TGGT1_273370-t26_1	713.6269703	1153.16296	-0.69235	0.00013498	0.0054356	putative coatomer gamma 2-subunit protein
TGGT1_222270-t26_1	610.0058598	985.0331865	-0.69135	9.83E-05	0.0042812	hypothetical protein
TGGT1_313080-t26_1	1474.069298	2370.626925	-0.68546	2.31E-05	0.0013567	hypothetical protein
TGGT1_217750-t26_1	592.8637832	951.3391416	-0.68226	0.00010718	0.0045565	hypothetical protein
TGGT1_203370-t26_1	951.0247262	1515.111295	-0.67187	5.47E-05	0.0027027	hypothetical protein
TGGT1_252280-t26_1	614.6562472	978.3594381	-0.67058	0.0003982	0.012851	hypothetical protein
TGGT1_287230-t26_1	315.1333464	500.0360158	-0.66607	0.00096215	0.025105	inorganic anion transporter, sulfate permease (SulP) family protein
TGGT1_232520-t26_1	449.8018253	713.1846958	-0.66499	0.00029278	0.0098792	brix domain-containing protein
TGGT1_319720-t26_1	298.9415168	473.8039448	-0.66443	0.0018689	0.039854	hypothetical protein
TGGT1_266300-t26_1	1290.246181	2044.720573	-0.66426	2.34E-05	0.0013663	hypothetical protein
TGGT1_228190-t26_1	1413.436269	2236.103793	-0.66178	6.08E-05	0.002952	putative eukaryotic initiation factor-3
TGGT1_294640-t26_1	3736.567949	5907.676903	-0.66088	9.95E-06	0.00068499	ribonucleoside-diphosphate reductase large chain
TGGT1_289800-t26_1	542.6157838	857.0483795	-0.65945	0.00059227	0.017584	emp24/gp25L/p24 family protein
TGGT1_258800-t26_1	322.5490895	509.1186728	-0.65848	0.00087863	0.023436	ROP31
TGGT1_301480-t26_1	221.8903932	350.0672392	-0.65778	0.0022916	0.045303	hypothetical protein
TGGT1_258060-t26_1	800.1644178	1260.569227	-0.65571	0.00017235	0.0064645	hypothetical protein
TGGT1_411270-t26_1	413.3714974	650.8453542	-0.65488	0.0012069	0.029778	hypothetical protein
TGGT1_231890-t26_1	657.3957289	1031.208263	-0.6495	0.00046568	0.014599	putative beta-ketoacyl-acyl carrier protein synthase III
TGGT1_214770-t26_1	752.0746194	1178.510237	-0.64802	0.00015668	0.0060388	putative small GTP binding protein rab1a
TGGT1_228290-t26_1	1713.486093	2678.666304	-0.64458	3.96E-05	0.0020685	phospholipase/carboxylesterase
TGGT1_283710-t26_1	1112.609721	1737.456999	-0.64303	0.0017622	0.03886	longevity-assurance protein (LAG1) domain-containing protein
TGGT1_217740-t26_1	1646.807973	2571.118705	-0.64272	3.77E-05	0.0020034	3-ketoacyl-(acyl-carrier-protein) reductase
TGGT1_224880-t26_1	1261.70818	1968.355029	-0.64161	0.0017517	0.038806	kinesin motor domain-containing protein
TGGT1_362090-t26_1	371.3044561	579.0381424	-0.64106	0.001744	0.038765	hypothetical protein
TGGT1_238040A-t26_1	537.2757755	836.6365436	-0.63894	0.00053863	0.016279	protein disulfide-isomerase domain-containing protein
TGGT1_218760-t26_1	498.8624872	775.5585748	-0.63659	0.0010767	0.027413	putative SNAP protein
TGGT1_312530-t26_1	766.51319	1189.213047	-0.63362	0.00028048	0.0096189	splicing factor, CC1 family protein
TGGT1_310240-t26_1	928.0569511	1438.928663	-0.63271	0.00024158	0.0085419	hypothetical protein
TGGT1_299210-t26_1	1649.778977	2550.396639	-0.62845	9.60E-05	0.0041991	CTP synthase
TGGT1_240280-t26_1	1093.145027	1687.129859	-0.62609	8.80E-05	0.0038697	S1/P1nuclease
TGGT1_280600-t26_1	2259.81748	3484.481163	-0.62474	4.67E-05	0.0023918	histidyl-tRNA synthetase
TGGT1_218362-t26_1	701.5470953	1081.005072	-0.62376	0.00038526	0.012516	zinc finger protein ZFP1
TGGT1_249480-t26_1	1086.643452	1674.258236	-0.62364	0.00014948	0.0058144	tetratricopeptide repeat-containing protein
TGGT1_201700-t26_1	1002.39426	1540.502274	-0.61995	0.00050718	0.01544	WD domain, G-beta repeat-containing protein
TGGT1_314750-t26_1	485.7825419	746.554498	-0.61994	0.00082666	0.022262	ribosomal L1p/L10e family protein
TGGT1_279420-t26_1	2336.995739	3574.449902	-0.61307	5.00E-05	0.0025317	precursor of Tsp1 domain TSP12
TGGT1_237250-t26_1	1160.251626	1772.683263	-0.6115	0.00047754	0.01486	emp24/gp25L/p24 family protein
TGGT1_224850-t26_1	4463.342385	6819.156997	-0.61147	2.19E-05	0.0012952	polyadenylate binding protein
TGGT1_300285-t26_1	375.3768956	573.2794082	-0.6109	0.0018052	0.039396	hypothetical protein
TGGT1_308050-t26_1	440.3051932	672.3653411	-0.61074	0.0024826	0.047732	thioredoxin domain-containing protein
TGGT1_253730-t26_1	1197.148745	1827.004397	-0.60988	0.0001354	0.0054356	importin-beta N-terminal domain-containing protein
TGGT1_229200-t26_1	460.4955863	702.4640872	-0.60924	0.0023248	0.045532	carboxy-terminal domain cyclin
TGGT1_270550-t26_1	510.6714277	778.8738631	-0.60899	0.0013982	0.032723	gamma-glutamyl phosphate reductase
TGGT1_290660-t26_1	3622.182121	5505.964498	-0.60414	0.0001124	0.0047219	RNA recognition motif-containing protein
TGGT1_263260-t26_1	2184.075675	3308.285693	-0.59906	2.75E-05	0.0015282	transporter, major facilitator family protein
TGGT1_209140-t26_1	572.9305802	867.0417333	-0.59774	0.0013144	0.031109	anti-silencing protein, ASF1 family protein
TGGT1_247470-t26_1	1391.803583	2090.532553	-0.58691	0.00012306	0.0051186	putative nucleolar protein
TGGT1_235920-t26_1	1269.602741	1904.163013	-0.58478	0.0001365	0.0054356	dynein, axonemal, heavy chain 2 family protein
TGGT1_210960-t26_1	519.1456549	776.5879563	-0.58101	0.0012001	0.029745	putative replication factor C subunit 2
TGGT1_217820-t26_1	1526.068838	2281.898504	-0.58041	0.00017086	0.0064375	Proteasome PCI domain-containing protein
TGGT1_269650-t26_1	1326.406776	1977.590378	-0.57622	0.00027117	0.0093761	FFD and TFG box motifs protein
TGGT1_300280-t26_1	593.4623476	881.9095577	-0.57147	0.0022701	0.045267	LSM domain-containing protein
TGGT1_305590-t26_1	1816.964606	2689.01412	-0.56555	0.00069487	0.019994	ABC transporter transmembrane region domain-containing protein member 7/9
TGGT1_282210-t26_1	846.9282338	1247.326403	-0.55853	0.0018515	0.039787	AP2 domain transcription factor AP2VIIa-8
TGGT1_203830-t26_1	1114.597835	1641.094523	-0.55814	0.00080714	0.022074	FHA domain-containing protein
TGGT1_309200-t26_1	1623.313791	2389.365212	-0.55769	0.00026771	0.0092947	zinc finger (CCCH type) motif-containing protein
TGGT1_253380-t26_1	3473.26062	5101.442823	-0.55461	0.00072744	0.020579	AP2 domain transcription factor AP2III-2
TGGT1_226830-t26_1	4129.832721	6062.056168	-0.55372	0.00040666	0.013041	DnaK family protein
TGGT1_209160-t26_1	1124.30527	1649.962457	-0.5534	0.0012093	0.029778	myristoyl CoA:protein N-myristoyltransferase
TGGT1_246500-t26_1	746.2867179	1093.455354	-0.55109	0.0021226	0.043274	SURP module domain-containing protein
TGGT1_293730-t26_1	1018.946363	1492.391436	-0.55055	0.001564	0.03523	zinc finger (CCCH type) motif-containing protein
TGGT1_269190-t26_1	6340.665133	9281.191038	-0.54968	0.00020482	0.0075148	glyceraldehyde-3-phosphate dehydrogenase GAPDH2
TGGT1_238180-t26_1	1148.672903	1672.970961	-0.54244	0.00057739	0.017359	putative 26s proteasome regulatory complex subunit
TGGT1_250955-t26_1	1971.614393	2868.170946	-0.54075	0.0006571	0.019237	KRUF family protein
TGGT1_297870-t26_1	1528.211577	2221.57801	-0.53974	0.00022978	0.0083215	hypothetical protein
TGGT1_297245-t26_1	1065.413474	1547.78944	-0.5388	0.0017196	0.038323	transporter, major facilitator family protein
TGGT1_258150-t26_1	1262.152637	1832.241434	-0.53772	0.00049507	0.01527	putative proteasome subunit alpha type 7
TGGT1_203450-t26_1	1015.959896	1472.737075	-0.53566	0.0015406	0.035027	DUF3228 domain-containing protein
TGGT1_311680-t26_1	870.3679548	1260.770998	-0.53461	0.00076372	0.021177	FUN14 family protein
TGGT1_233100-t26_1	996.7489568	1443.693614	-0.53446	0.0018924	0.040117	SPFH domain / Band 7 family protein
TGGT1_243710-t26_1	2235.108989	3233.841899	-0.5329	0.00029155	0.0098775	t-complex protein beta subunit
TGGT1_272740-t26_1	1419.035702	2050.986992	-0.53141	0.00050592	0.01544	DNA-directed RNA polymerase III RPC2
TGGT1_226430-t26_1	1825.78416	2632.093062	-0.52769	0.00067115	0.019512	reticulon protein
TGGT1_215060-t26_1	1019.327252	1465.857238	-0.52413	0.0019646	0.041335	putative small GTP-binding protein sar1
TGGT1_287500-t26_1	1946.576554	2792.146914	-0.52044	0.00050407	0.01544	putative T complex chaperonin
TGGT1_315360-t26_1	1235.510492	1771.316687	-0.51971	0.0010058	0.026081	hypothetical protein
TGGT1_266366-t26_1	3688.54292	5286.010964	-0.51913	0.00101	0.026112	BT1 family protein
TGGT1_290600-t26_1	1381.534864	1977.975591	-0.51775	0.0015414	0.035027	succinyl-CoA-synthetase alpha
TGGT1_227650-t26_1	1086.36221	1554.384419	-0.51684	0.0014825	0.034221	putative microtubule-associated protein RP/EB family
TGGT1_219520-t26_1	1564.778832	2234.548928	-0.51402	0.00072215	0.020498	histone arginine methyltransferase PRMT1

TGGT1_269980-t26_1	4566.659402	6508.400146	-0.51116	0.00059695	0.01766	putative preprotein translocase Sec61
TGGT1_275610-t26_1	2387.34974	3394.214085	-0.50767	0.0015388	0.035027	protein kinase
TGGT1_256000-t26_1	1468.523641	2087.611642	-0.50749	0.0010485	0.026776	ER retention receptor ERD2
TGGT1_229180-t26_1	1968.036234	2792.63733	-0.50487	0.0010237	0.026224	HEAT repeat-containing protein
TGGT1_206610-t26_1	1614.841282	2286.272864	-0.50161	0.0015576	0.03523	pyruvate dehydrogenase complex subunit PDH-E2
TGGT1_310750-t26_1	2357.76408	3331.157714	-0.4986	0.0012396	0.030112	emp24/gp25L/p24 family protein
TGGT1_288920-t26_1	2134.928594	3016.120344	-0.49851	0.0012469	0.030112	phosphonate-transporting ATPase
TGGT1_262380-t26_1	1127.139348	1589.204414	-0.49564	0.0018677	0.039854	putative elongation factor Tu
TGGT1_257550-t26_1	1752.865091	2460.813643	-0.48942	0.0017938	0.039352	hypothetical protein
TGGT1_203600-t26_1	1464.754779	2048.902856	-0.48419	0.0022981	0.045325	hypothetical protein
TGGT1_254820-t26_1	1324.718803	1850.652221	-0.48235	0.0023918	0.046518	hypothetical protein
TGGT1_243920-t26_1	2316.187883	3226.773242	-0.47834	0.0012121	0.029778	putative DNA replication licensing factor MCM5
TGGT1_214970-t26_1	4346.055993	6049.141373	-0.47702	0.0011121	0.027975	putative DNA replication licensing factor MCM2
TGGT1_270510-t26_1	6619.576552	9195.966194	-0.47426	0.00077432	0.021401	asparaginyl-tRNA synthetase
TGGT1_363030-t26_1	13361.94874	18530.50723	-0.47177	0.00066371	0.019363	rhoptry protein
TGGT1_273460-t26_1	3643.800895	5049.942245	-0.47082	0.00080917	0.022074	eukaryotic translation initiation factor 3 subunit 6 interacting protein
TGGT1_243200-t26_1	6124.62741	8480.15355	-0.46947	0.0023144	0.045433	hypothetical protein
TGGT1_218240-t26_1	3878.562833	5349.939708	-0.464	0.0025364	0.048543	hypothetical protein
TGGT1_213570-t26_1	2179.030654	2996.12985	-0.45941	0.0010934	0.027746	hypothetical protein
TGGT1_411430-t26_1	3125.001252	4290.479996	-0.45728	0.0022568	0.045146	ROP5
TGGT1_251690-t26_1	3025.946861	4148.675075	-0.45526	0.0014172	0.033076	putative seryl-tRNA synthetase
TGGT1_314400-t26_1	2123.387669	2886.432828	-0.44292	0.0021828	0.044087	putative pyruvate dehydrogenase E1 component
TGGT1_226960-t26_1	9838.558483	13352.70477	-0.44061	0.0018425	0.039787	phosphofructokinase PFKII
TGGT1_318310-t26_1	1879.042679	2549.543125	-0.44024	0.0019844	0.041475	transketolase
TGGT1_311240-t26_1	3819.110786	5143.509539	-0.42952	0.002413	0.046821	putative DnaJ family chaperone
TGGT1_225050-t26_1	4222.05482	5661.093664	-0.42314	0.0021116	0.043217	putative adenosylhomocysteinase
TGGT1_261950-t26_1	5913.201426	7794.848041	-0.39858	0.0022382	0.045097	ATP synthase beta subunit ATP-B
TGGT1_290200-t26_1	5634.519024	7396.337087	-0.39252	0.0022736	0.045267	NAD/NADP octopine/nopaline dehydrogenase
TGGT1_240210-t26_1	0	743.3530468	#NAME?	3.27E-45	3.05E-42	putative phosphate transporter family protein

S5 Table. ToxoDB

KOPiTvsPar.DEG_down sort

ran
