# Supplementary material for: A single Na+-Pi cotransporter in Toxoplasma plays key roles in phosphate import and control of parasite osmoregulation
Source: PLoS Pathog. 2020 Dec 31;16(12):e1009067. doi: 10.1371/journal.ppat.1009067 (PMC7817038; doi:10.1371/journal.ppat.1009067)
Supplement: Attachment — Submitted filename: Rebuttal letter.docx [file ppat.1009067.s012.docx]

**Answers to Reviewer #1:**

Major comments:

*1 - Concerning the ion-dependence of TgPiT for P_i_ internalization into Toxoplasma:*

We agree that there is no evidence for the presence of Na^+^-K^+^-ATPases or H^+^-K^+^-ATPases expressed in *Toxoplasma* and that our previous model on P_i_ uptake by *Toxoplasma* presented in ex-Figure 4B was highly speculative. We deleted this Figure. We revised our assays to determine the contribution of an external Na^+^ gradient to drive P_i_ internalization into the parasite (new Fig. 1G). We monitored the effect of cipargamin, an inhibitor of Na^+^ efflux, based on the genetic evidence that *Toxoplasma* expresses a functional plasma membrane localized P-type Na^+^-H^+^-ATPase (ATP4; ref. 31). Data show a significant decrease in P_i_ uptake proportional to cipargamin concentration, indicating a synergistic role for Na^+^ in P_i_ uptake (P6L26).

*2 - Concerning the determination of the 2Na^+^:1H_2_PO_4_^-^ stoichiometry:*

We added to the Materials and Methods section the equations we used to calculate the parameters of P_i_- and Na^+^-dependence on P_i_ uptake by the parasite, including the Hill coefficient to determine the stoichiometry (P27L15). We also provided a better explanation of the sigmoidal Na^+^ concentration-dependence of Pi uptake in Fig. 1C, with a cooperativity index (Hill coefficient) higher than 1, and the P_i_ concentration-dependence of P_i_ uptake that exhibits Michaelian behavior (in Fig. 1D) in the Results section (P6L13) and Discussion section (P21L7).

*3-4 - Concerning the PCC calculation:*

We did not calculate PCC for intracellular *Toxoplasma* in Fig. 3B. We specified that the PCC values for extracellular parasites were calculated on the entire parasite body (Fig. 3G). We provided means ± SD for PCC values calculated from 50 (normal PO_4_) and 65 (low PO_4_) parasites, plus statistical analysis in the figure legend.

*5 - Concerning loading controls for our genetic strains:*

We performed new Western blots on parasite lysates from our 3 strains and used actin as a loading control (Fig. S5A).

*6 - Concerning the change of PAM by mutagenesis when doing the complementation:*

We did not change the PAM as the original plasmid pSAG1::CAS9-U6::sgUPRT used for the complementation of the knockout strain was already designed to generate a double-strand break at the UPRT gene.

*7 -* We performed statistical analysis for the *in vivo* virulence studies using the Log-rank (Mantel-Cox) test.

*8 - Concerning the increase in size of acidocalcisomes as a compensatory mechanism in the knockout to control osmolarity:*

We thank this reviewer for this pertinent comment. Indeed, the diversity in acidocalcisome size and electron density, as well as the associated loss in volume of ΔTgPiT parasites may certainly be attributed to various levels of activities for polyphosphate hydrolysis in the acidocalcisome population, leading to increased osmolarity and water intake in these organelles. This is interesting especially since polyphosphatase activity has only been observed in *Toxoplasma* but not attributed to any specific organelle. We discuss this point in the Discussion section at P24L30.

*9 - RNA seq data*

Although we did not validate our RNAseq data by qPCR, we performed RNAseq analysis on 3 independent parasite preparations, with high reproducibility of data from the replicate samples. In this case, we would prefer to keep the paragraphs of our gene analysis in the Results section. In the future, if we want to further investigate a specific gene with altered expression in the knockout, we will verify its expression profiles by qPCR.

Minor points

-We use singular for PP_i_.

-We deleted information on P_i_ concentrations based on absorbance values. We have provided more accurate P_i_ concentration measurements based on colorimetric assays using malachite green and ammonium molybdate in Fig.6.

-We replace ‘sulfur’ by ‘sulfate’; ‘energize’ by ‘drive’. We changed ‘intraparasitic’ to ‘intracellular’, and we corrected ‘vacuolar ATPase synthase’ to ‘vacuolar ATPase subunit c’.

-We replaced correctly the references in the text. We spelled out all abbreviations (PCC, TbHMIT. UPRT).

-We rectified our mistake regarding P_i_ concentration in panel b Figure 1J, panel b and provided explanations on pH assays and dependence in the Results section (P7L12) and legend of Figure 1J.

_________________________________________________________________________________________

**Answers to Reviewer #2:**

Major comments:

*1 - Concerning the ion-dependence of TgPiT for P_i_ internalization into Toxoplasma:*

We agree that our previous model on P_i_ uptake by *Toxoplasma* presented in ex-Figure 4B was highly speculative, especially in the absence of evidence of a defined drug target. We deleted this Figure. We revised our assays to determine the contribution of an external Na^+^ gradient to drive P_i_ internalization into the parasite (new Fig. 1G). We monitored the effect of cipargamin, an inhibitor of Na^+^ efflux, based on the genetic evidence that *Toxoplasma* expresses a functional plasma membrane localized P-type Na^+^-H^+^-ATPase (ATP4; ref. 31). Data show a significant decrease in P_i_ uptake proportional to cipargamin concentration, indicating a synergistic role for Na^+^ in P_i_ uptake (P6L26).

*2 - Concerning alterations in cell volume and ionic regulation for ΔTgPiT parasites*:

We provided new data comparing the cell volume of ΔTgPiT, parental and complemented parasites with volume gating using a Coulter counter (new Fig. 7B). Data show that the knockout parasites have a ~30% decrease in cell volume compared to control parasites (P12L12). Related to the reduced cell volume in ΔTgPiT parasites, we also provided new data showing that ΔTgPiT parasites display dysregulation of [Na^+^] and [Ca^2+^] stores, and alkalinization of the cytosol (new Fig. 12) (P15L13). We also draw a model describing ion homeostasis and osmoregulation in the parasite (new Fig. 14).

*3 - Concerning Pi measurement in Toxoplasma based on absorbance values:*

We deleted from the text absorbance values for P_i_ concentration determinations in the parasite and fibroblasts. We have provided in Fig. 6 more accurate measurements of total P_i_ and polyP concentrations in *Toxoplasma*, based on colorimetric assays using malachite green and ammonium molybdate. We rephrased our previous statement “*Toxoplasma* actively accumulates exogenous P_i_ against a concentration gradient” to “*Toxoplasma* internalizes inorganic phosphate by using a sodium gradient” to better reflect the data.

*4 - Concerning our assays of P_i_ uptake as a function of pH:*

We rephrased entirely this section for more clarity in the Results section (P7L12), Discussion section (P21L4) and legend of Figure 1J.

*5 - Concerning RNAseq data:*

We removed ex-Fig. 14A as these raw data were obtained before the application of statistics. Fig. 13A shows the Log_2_ (fold change) and the adjusted p-values for the altered genes. For the fold change, we specified that it refers to Log_2_ fold change in the text. We placed the sulfate permease in the paragraph discussing transporters. We changed ‘transmembrane movement of acid substances’ to ‘transmembrane movement of H^+^ ions’.

*5* - We replaced ‘a unique Na^+^-P_i_ cotransporter’ with ‘a single Na^+^-P_i_ cotransporter’ in the title; and ‘parasites were syringed’ by ‘parasites were syringe-released by repeated passage through needles’.

*6 -* We provided all the missing statistical analyses (Figures 1, 4, 5, 9, S4, S5 and Table 1), erroneous figure citations, and the added sulfate concentrations in the P_i_ uptake assays in Fig. 1F.

*7* *-* We rephrased our previous statements about TgPiT requirements for virulence and osmoregulation (P11L17 and P20L10).

*8 -* We specified that the solubilized fraction corresponds to the fraction of proteins that is soluble in the detergent.

*9 -* Concerning the comparison in replication rate between parental and ΔTgPiT parasites, under normal or low phosphate condition: we rephrased our paragraph in P13L26. Fig. 9A reveals a more dramatic replication delay between parental and ΔTgPiT parasites under the low phosphate condition.

*10* *-* Concerning VAC dysfunctions based on abnormal VAC morphology in ΔTgPiT parasites: we deleted from the Results section our interpretations about this phenotype and instead proposed in the Discussion section that the enlargement of VAC in the knockout could be due to compromised endoproteolytic activities as VAC is involved in protein degradation [72,74]. We provide a description of VAC in extracellular WT parasites for comparison with ΔTgPiT parasites in P15L3.

*11 -* Concerning our radiolabeled Pi uptake measurements: for each experiment involving ^32^P_i_ uptake, we used as a negative control paraformaldehyde-treated parasites and subtracted the radioactive values of killed parasites (corresponding to < 5% cpm) from radioactive values associated with live parasites. We added this information in the Materials and Methods section (P27L27).

_________________________________________________________________________________________

**Answers to Reviewer #3:**

Major comments:

*1-3 - Concerning the ion-dependence of TgPiT for P_i_ internalization into Toxoplasma:*

We agree that there is no genetic or physiological evidence that an extracellular gradient of Na^+^ is maintained by a Na^+^-K^+^-ATPase in *Toxoplasma*. We concur that our previous model on P_i_ uptake by *Toxoplasma* presented in ex-Figure 4B was highly speculative, in the absence of evidence of a defined drug target for ouabain and omeprozale. We deleted this Figure. We revised our assays to determine the contribution of an external Na^+^ gradient to drive P_i_ internalization into the parasite (new Fig. 1G). We monitored the effect of cipargamin, an inhibitor of Na^+^ efflux, based on the genetic evidence that *Toxoplasma* expresses a functional plasma membrane localized P-type Na^+^-H^+^-ATPase (ATP4; ref. 31). Data show a significant decrease in P_i_ uptake proportional to cipargamin concentration, indicating a synergistic role for Na^+^ in P_i_ uptake (P6L26).

*4 - Concerning TgPiT localization by IFA or transfection*

We agree that transient transfection of cells with a plasmid can lead to overexpression, and thus mislocalization of a protein. Only panel a in Fig. 3A shows transfected parasites expressing TgPiT-mCherry (clarification in the new figure legend). The mCherry pattern has been then verified using anti-TgPiT antibodies by IFA, as illustrated in parasites shown in the other panels of Fig. 3 and by immunoEM.

_________________________________________________________________________________________
