# Supplementary material for: A single Na+-Pi cotransporter in Toxoplasma plays key roles in phosphate import and control of parasite osmoregulation
Source: PLoS Pathog. 2020 Dec 31;16(12):e1009067. doi: 10.1371/journal.ppat.1009067 (PMC7817038; doi:10.1371/journal.ppat.1009067)
Supplement: Attachment — Submitted filename: Answers to Reviewer#2.docx [file ppat.1009067.s013.docx]

**Answers to Reviewer #2:**

*- Concerning the intracellular Na^+^ measurements comparing Fluorescence Ratios for different parasite lines*: we concur that data presented in ex-Fig. 12A were suboptimal, with an averaged Fluorescence Ratio of 1 for TgPiT-deficient *Toxoplasma*. In this case, we cannot exclude an issue of dye incorporation into the mutant. We removed these data from the revised manuscript. For the future, we will repeat these assays on intracellular Na^+^ measurements with full calibrations for the mutant, parental and complemented cell lines, comparing several strains of *Toxoplasma*.

We agree that it could be considered speculative to assume that removal of one Na^+^ intake pathway (eg., TgPiT gene deletion), would reduce basal Na^+^ concentration through impacting the activity of other Na^+^ pumps/transporters at the plasma membrane of the parasite; we have no experimental evidence for such compensatory activities. The rationale of our hypothesis was based on the fact that TgPiT-deficient *Toxoplasma* survive despite exhibiting growth defects and ionic imbalance, which leads to the assumption that some compensatory mechanisms, such as possible down-regulation of TgATP4, should be in place. To avoid confusion, we introduced interrogation marks in our model (Fig. 14) for TgATP4, TgNHE1 and V-H^+^-ATPase, reflective of potential changes in their activities.

*- Concerning the statement about the inability of TgPiT knockout parasites to regulate their cytosolic Na^+^ and H^+^ concentrations*: We thank this reviewer for this pertinent comment. In the revised version, we clarified that, although there are measurable ionic defects in the TgPiT knockout parasites, the mutant manages to maintain ion levels to almost normal values. This suggests that the compensatory mechanisms activated by TgPiT knockout parasites for correcting ion imbalance is sufficient for parasite survival and the preservation of ionic homeostasis, but insufficient in eliminating all ionic deficiencies arising from the elimination of TgPiT.

*- Concerning the pH experiments insufficiently described*: In the Materials and Methods section, we provide experimental details, including the composition of the solution to resuspend the parasites at resting pH.

*- Concerning the Ca^2+^ measurements using calibration procedure showing Fluorescence Ratios:* We appreciate the suggestion to preferably use established methods involving the use of ionophores on our future assays. We agree that upon parasite lysis, the localization and properties of a fluorescent dye loaded into a cell may be affected; however, although there may be disadvantages, the use of detergents instead of an ionophore is not an uncommon method for calibration of other ratiometric ionic indicators like Fura2-AM. Results were initially presented as a ratio to remain consistent with the presentation of SBFI-am ratiometric data, but upon removal of SBFI-AM data and at the recommendation of the reviewer, we have converted our ratios to cytosolic calcium concentrations using the Grynkiewicz, Poenie & Tsien formula and method to provide additional information to our results (new Fig. 12A). We confirm that the mutant has comparable resting cytosol [Ca^2+^] with parental and complemented parasites but it shows some dysfunctions in the release of stored calcium.

*- Concerning the complexity in the design of our Coulter Counter experiment*: As recommended, we have added a new Fig. S6 to include raw data from our 3 independent experiments presented in graphs with the y axis representing the raw parasite count and decline as gating volume increased. Our materials and methods were updated to provide details on how these 3 curves showing raw population decline were converted to percentage population decline and used to determine average parasite size.
